# The Intracavity Extension of 28-Hetero-2,7-naphthiporphyrins in Reactions with Alkylamines

**DOI:** 10.1021/acs.orglett.3c01715

**Published:** 2023-06-22

**Authors:** Katarzyna Ślusarek, Jędrzej P. Perdek, Agata Białońska, Rafał A. Grzelczak, Bartosz Szyszko

**Affiliations:** University of Wrocław, Faculty of Chemistry, 14 F. Joliot-Curie St., 50-383 Wrocław, Poland

## Abstract

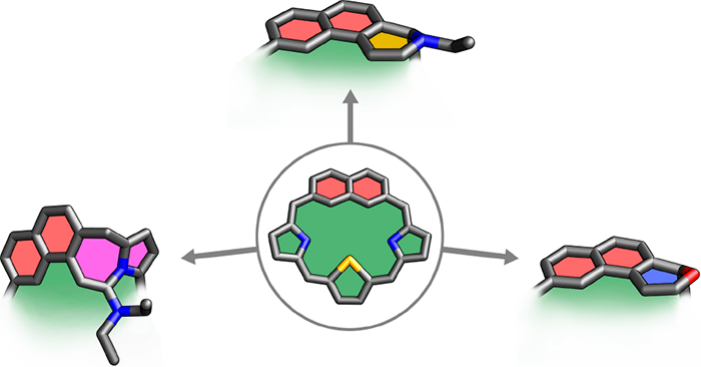

The 28-hetero-2,7-naphthiporphyrins reacted with triethylamine and diethylamine to form nonaromatic intracavity-extended macrocycles incorporating naphthodihydro-2*H*-pyran, naphthotetrahydropyridine, and naphthopyrrolotetrahydro-1*H*-azepine moieties. The new macrocycles were characterized in solution by means of NMR and UV–vis spectroscopy and in the solid state by XRD.

Carbaporphyrinoids, namely, porphyrin analogs incorporating carbon atoms within the cavity, have emerged as intriguing macrocyclic platforms demonstrating novel and exciting properties and unusual reactivity.^1,2^ They demonstrated the ability to form organometallic compounds and acted as aromaticity^3^ and conformation switches.^3^ Recently, they have been exploited as the intriguing building blocks of complex supramolecular systems such as molecular cages.^4^

Among the vast class of carbaporphyrinoids, those incorporating two and more carbon atoms within the cavity remain rare.^5−9^ Examples of such systems include naphthiporphyrins, namely, carbaporphyrinoids incorporating at least one naphthalene moiety replacing the pyrrole ring in the porphyrin macrocycle.^10−12^ Recently, we have demonstrated that the horizontal expansion of the *m*-benziporphyrin^13,14^ framework could provide the 28-hetero-2,7-naphthiporphyrins **1-X** (X = S, Se, Te), which act as macrocyclic ligands for phosphorus(V).^15,16^

The carbaporphyrinoid macrocycles, aside from their role as unusual ligands in coordination and organometallic chemistry, often enable peculiar reactivity within the macrocyclic cavity.^2^ Although the core chemistry of N-confused porphyrin,^17,18^ the archetypical carbaporphyrinoid, is very well documented,^19^ other carbaporphyrinoids are much less studied in this context despite the fact that they were shown to undergo remarkable transformations. An unusual oxidative acetoxylation^13^ and regioselective pyridination^20^ were reported for *m*-benziporphyrin, whereas both *m-* and *p-*benziporphyrins demonstrated peculiar phenylene contractions transforming benziporphyrins into 21-carbaporphyrins.^12,21,22^

Herein we report the unusual reactivity of 28-hetero-2,7-naphthiporphyrins with alkylamines that results in their intracavity extension, providing new carbaporphyrinoids incorporating naphthodihydro-2*H*-pyran, naphthotetrahydropyridine, and naphthopyrrolotetrahydro-1*H*-azepine moieties.

The reactions of 28-selena-2,7-naphthiporphyrin **1-Se** with triethylamine (TEA) and diethylamine (DEA) were carried out in neat purified alkylamines^23,24^ (see the SI for details) under aerobic conditions for 24 h at room temperature. Remarkably, the MS and NMR analyses of the mixture obtained from the reaction of **1-Se** with DEA revealed the formation of two products, **2-Se** and **3-Se** (Scheme 1). Under identical conditions, **1-Se** and triethylamine yielded **2-Se** and **4-Se**. When nonpurified TEA or DEA was exploited for the reactions, **2-Se**, **3-Se**, and **4-Se** were all isolated in both cases, accompanied by several other species not unambiguously identified due to their minute yields and decomposition during chromatographic purification (Figures S101–108, SI). This peculiar observation can be rationalized by considering TEA/DEA contamination with a small amount of other alkylamines.^25^ Carrying out the reactions at reflux resulted in mixtures with a similar composition but a slightly different product ratio. Depending on the purity/type of the alkylamine and reaction conditions, **2-Se**, **3-Se**, and **4-Se** were isolated in 2–45%, 3–49%, and 7–51% yields, respectively. In the case of the reaction of **1-S** with triethylamine, only **4-S** (43%) could be isolated.

**Scheme 1:**
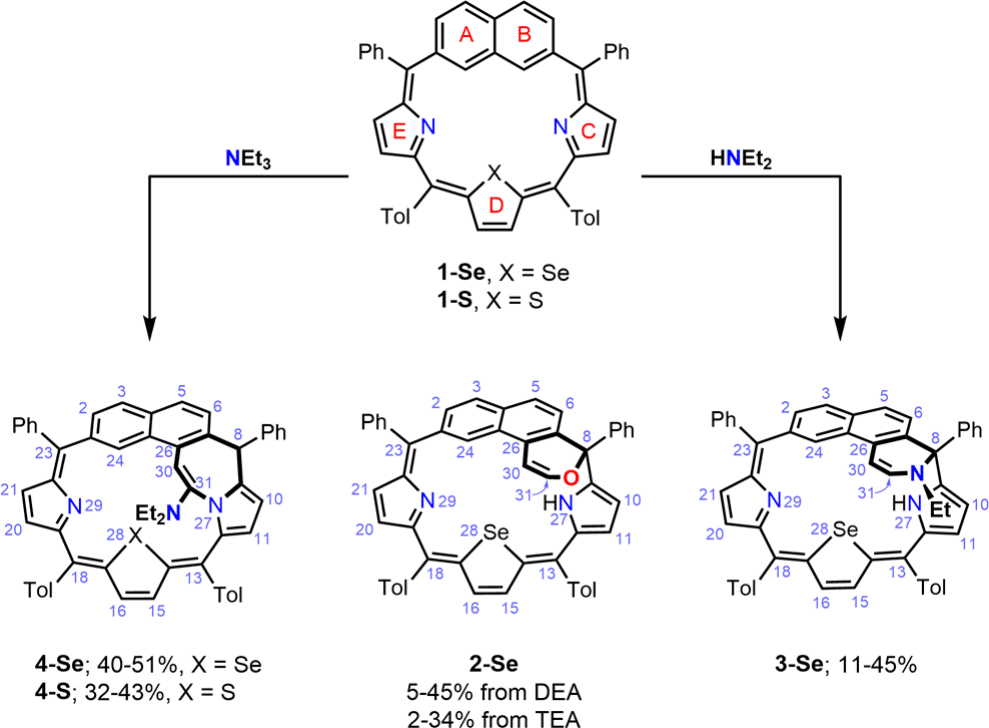
Synthesis of **2-Se**, **3-Se**, and **4-Se**

The elemental composition of **2-Se** was confirmed by high-resolution mass spectrometry; the signal at *m*/*z* = 811.2205 corresponded well to the calculated [M + H]^+^ (C_54_H_39_N_2_OSe^+^) value of 811.2228 (Figure S111, SI).

The unexpected reaction product incorporated the naphthodihydro-2*H*-pyran moiety, formally created by introducing the vinyl alkoxide bridge between C26 of the B ring of naphthalene and the C8 *meso*-carbon. Similarly, **3-Se** encompassed the N-ethylethenamine bridge, as demonstrated by the high-resolution mass spectrum showing the *m*/*z* signal at 838.2724, well-matching the 838.2702 value calculated for the C_56_H_44_N_3_Se^+^ formula of [M + H]^+^ (Figure S112, SI).

The transformation of the carbocyclic unit of **1-Se** was apparent upon analysis of the ^1^H NMR spectrum of **2-Se** (Figure 1A). In particular, a single resonance corresponding to the C24–H of ring A occurred at 9.73 ppm. The NH group of pyrrole C gave a broad resonance at 9.69 ppm and, in addition, two doublets (^3^*J* = 5.8 Hz) corresponding to C30–H and C31–H were identified at 7.47 (overlapping with other signals) and 7.28 ppm, respectively. The evaluation of the ^1^H-^13^C HMQC allowed for the assignment of the corresponding ^13^C NMR resonances at 102.9 and 148.2 ppm, respectively (Figures S19 and S20, SI). Furthermore, the C8 *meso*-carbon sp^2^ (**1-Se**) → sp^3^ (**2-Se**) rehybridization was evident from the corresponding ^13^C NMR spectrum, demonstrating the resonance at 82.1 ppm (Figures S11, S12, S21, and S22, SI). Furthermore, the peculiar orientation of C8–Ph with respect to naphthalene ring B resulted in the location of C6–H in the shielding zone of the phenyl substituent, resulting in an unusual chemical shift of 6.72 ppm. The position of β-pyrrole and β-selenophene resonances in the 5.6–7.1 ppm range indicated the nonaromaticity of **2-Se**.

**Figure 1:**
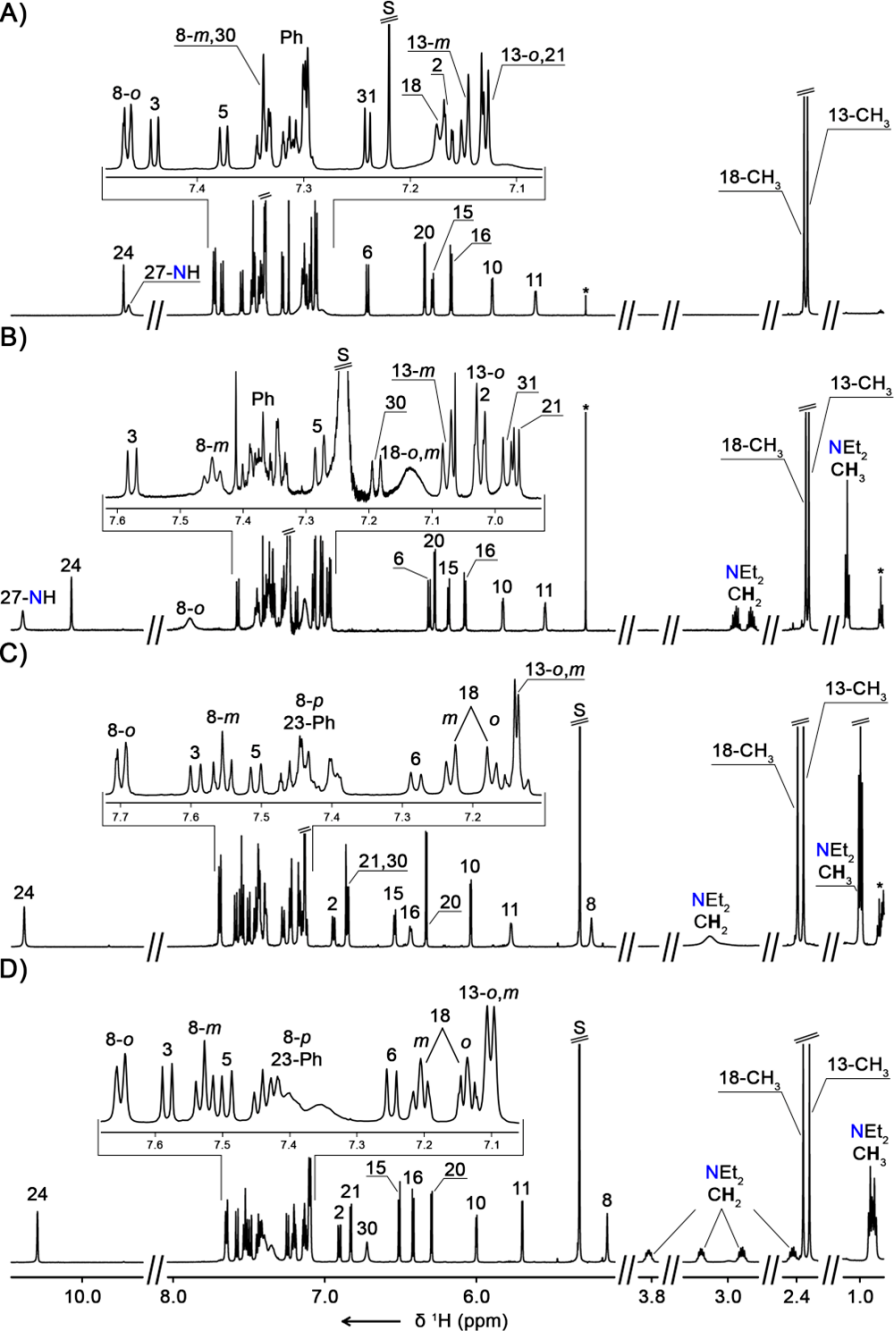
^1^H NMR spectra (600 MHz) of (A) **2-Se** (CDCl_3_, 300 K), (B) **3-Se** (CDCl_3_, 300 K), (C) **4-Se** (CD_2_Cl_2_, 300 K), and (D) **4-Se** (CD_2_Cl_2_, 220 K). Signals corresponding to impurities are marked with asterisks.

The ^1^H NMR spectrum of **3-Se** exhibited features very similar to those of **2-Se**, yet the critical differences between the two macrocycles were revealed upon analyzing the alkyl region (Figure 1B). In particular, two diastereotopically split CH_2_ resonances in the 2.8–3.0 ppm range and the corresponding triplet at 1.09 ppm unambiguously indicated the incorporation of the ethyl group attached to the amine nitrogen of the bridge. The position of the *N*-ethyl unit within the macrocyclic cavity was confirmed through the analysis of the NOE (nuclear Overhauser effect) map (Figures S41, S42, S53, and S54, SI). In particular, the presence of the −N–**CH**_**2**_···C31–H, −N–CH_2_–**CH**_**3**_···C31–H, and C30–H···C24–H NOE contacts was consistent with the proposed structure of **3-Se**. In addition, the ^13^C resonances of C30 and C31 were found at 95.6 and 138.1 ppm, respectively (Figures S43 and S44, SI).

The elemental composition of **4-Se** was established through high-resolution mass spectrometry (Figure S113, SI). The [M + H]^+^ molecular ion demonstrated an *m*/*z* of 866.3059, corresponding well with the value of 866.3015 simulated for the formula C_58_H_48_N_3_Se^+^ of **4-Se**.

**4-Se** structurally differed from the previous macrocycles, embedding a naphthopyrrolotetrahydro-1*H*-azepine moiety as a result of the incorporation of the *N*,*N*-diethylethenamine into the cavity in a way enforcing the formation of the *N*,*N*-diethylaminoethene bridge joining the C26 of naphthalene ring B and N27 of the pyrrole C. Similarly to **2-Se** and **3-Se**, the C8 carbon underwent rehybridization from sp^2^ to sp^3^, but in this case the hydrogen atom was attached to the *meso*-position.

Although the ^1^H NMR spectrum of **4-Se** demonstrated considerable similarity to **2-Se** and **3-Se**, careful analysis has revealed the differences in accordance with the proposed structure of the macrocycle (Figure 1C). In particular, the presence of a N-substituted ethene bridge linking C26 and N27 was evident upon identifying the C30–H singlet at 6.87 ppm, correlating to the ^13^C resonance at 98.7 ppm in the ^1^H-^13^C HMQC spectrum (Figure 1C and Figures S69 and S70, SI). The unresolved broad C8–H signal at 5.24 ppm was assigned based on the C8–*ortho*-Ph···C8H and C6–H···C8–H NOE cross-peaks in the ROESY (rotating frame nuclear Overhauser effect spectroscopy) map as well as through four-bond scalar coupling to pyrrolic C10–H observed in the ^1^H-^1^H COSY (correlation spectroscopy) spectrum (Figures S73–76, SI). In addition, the chemical shift of 49.0 ppm corresponding to C8 in the ^13^C NMR spectrum indicated the sp^3^ hybridization of this carbon atom (Figures S63 and S69, SI). Peculiarly, at 300 K, only a single broad resonance at 3.13 ppm was present in the spectral region expected for the methylene group of NEt_2_ substituents (Figure 1C). However, upon lowering the temperature to 220 K, four multiplets at 3.82, 3.18, 2.91, and 2.43 ppm corresponding to the CH_2_ protons of the two *N*-ethyl groups appeared (Figures 1D, S79, SI). This indicated that the rotation around the C31–NEt_2_ single bond at room temperature is not completely limited despite considerable crowding within the cavity of the **4-Se** macrocycle. Adversely for **2-Se** and **3-Se**, the *N*,*N*-diethylaminoethene bridge connected the naphthalene ring B with pyrrole C, not the C8 *meso*-carbon.

Eventually, the identities of **2-Se**, **3-Se**, and **4-Se** were confirmed in the solid state. The X-ray molecular structure of **2-Se** corroborated the proposed molecular formula depicted in Scheme 1 (Figure 2A). The macrocyclic framework incorporated the naphthodihydro-2*H*-pyran formed through the attachment of a vinyl alcohol-derived bridge linking C26 and *meso*-C8 through the carbon and oxygen atoms, respectively. The macrocycle adopted a folded conformation with a 62.3° dihedral angle between naphthodihydro-2*H*-pyran and the plane of the *meso*-carbon atoms. The C7–C8 and C8–C9 bond lengths equal to 1.545(8) and 1.508(7) Å indicated that the C8 carbon underwent rehybridization to a tetrahedral geometry connecting the naphthalene ring B with pyrrole C through the C(sp^2^)–C(sp^3^) and C(sp^3^)–C(sp^2^) single bonds, respectively.^26^ The C23–C1 distance of 1.461(8) Å suggested that the C(sp^2^)–C(sp^2^) single bond remained unaltered. The C26–C30, C30–C31, and C31–O bond lengths equal to 1.457(7), 1.352(7), and 1.349(6) Å, respectively, were in the range reported for *iso*-chromene derivatives, confirming the double-bond character of the C30–C31 bridge.^27−29^

**Figure 2:**
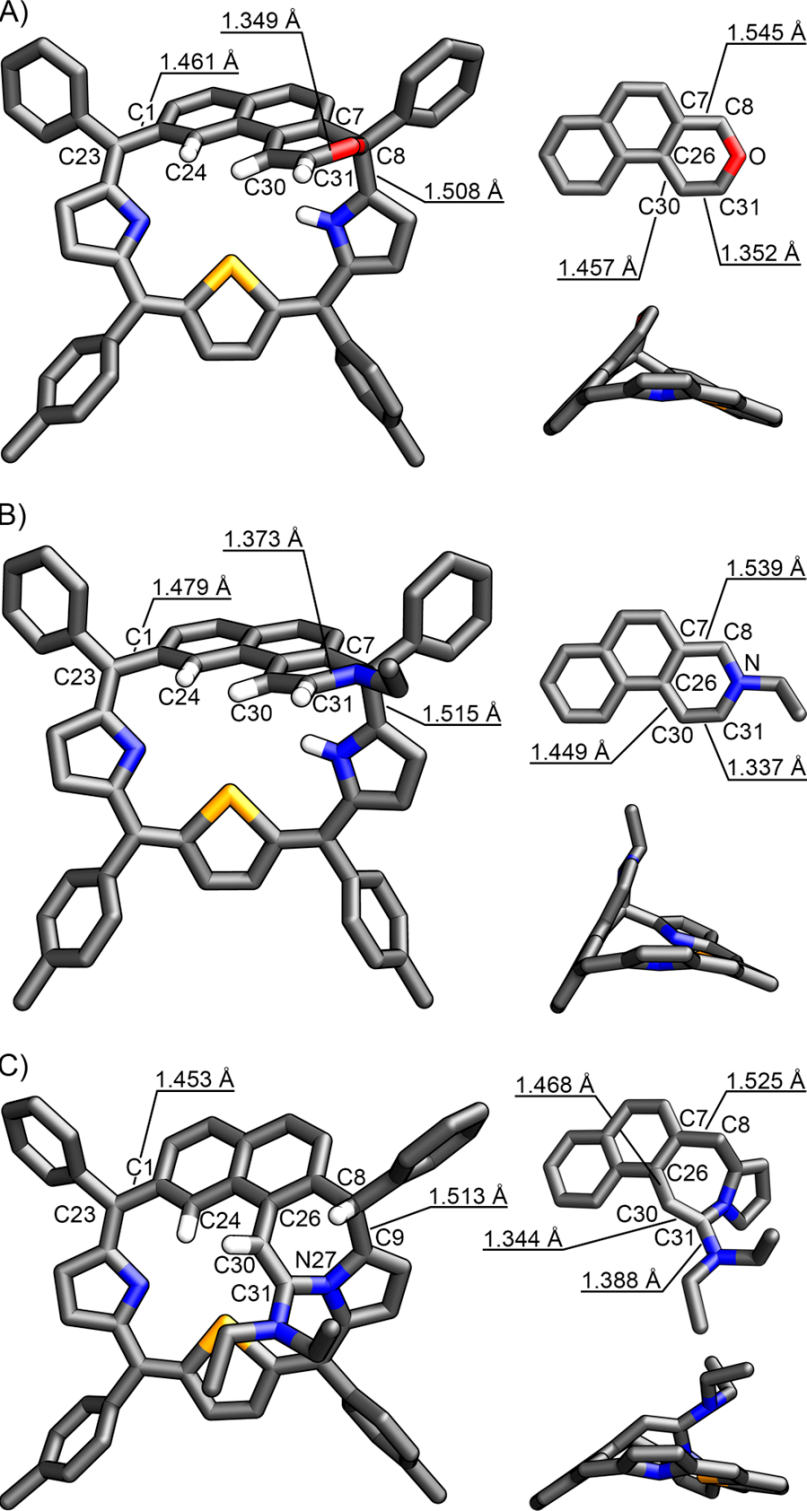
X-ray molecular structure (top, front view; bottom, side view) of (A) **2-Se**, (B) **3-Se**, and (C) **4-Se**. Insets present the heterocyclic moiety incorporated within the macrocyclic framework. Only selected protons are shown for clarity.

The molecular structure of **3-Se** demonstrated several similarities to that of **2-Se**, with the differences resulting from the presence of the ethyl-substituted nitrogen atom in place of oxygen in **2-Se** (Figure 2B). The C7–C8 and C8–C9 bond lengths equal 1.539(4) and 1.515(4) Å, respectively, indicated single-bond character, implying the tetrahedral geometry of the C8 *meso*-bridge. The interatomic distances within the newly formed heterocyclic ring corresponded well to the crystal data reported for dihydropyridine derivatives.^30,31^ In particular, the C30–C31 bond length of 1.337(4) Å indicated double-bond character.

The nature of the heterocycle incorporated within the **4-Se** cavity strongly differed from those of **2-Se** and **3-Se** (Figure 2C**)**. In particular, the *N*,*N*-diethylethenamine bridge connected naphthalene ring B with pyrrole C, forming the unsaturated heterocycle composed of seven atoms, i.e., 2,3,4,5-tetrahydro-1*H*-azepine. In **4-Se**, the pyrrole C nitrogen atom was involved in forming a bridge. Similar reactivity of the pyrrole of the macrocycle was previously demonstrated for N-fused porphyrins and carba- and heteroporphyrinoids.^32−37^ The tetrahydroazepine ring adopted a boatlike conformation enforcing the *endo*-position of a hydrogen atom at the tetrahedral C8. The C30–C31 distance of 1.344(5) Å indicated a double-bond character, consistent with the valence structure depicted in Scheme 1. The nitrogen atom of the *N*,*N*-diethylamine group was located ca. 2.3 Å above the mean plane of pyrrole B and selenophene rings and was connected to C31 through a single bond of 1.388(4) Å length.

The macrocycles **2-Se**, **3-Se**, and **4-Se**, dissolved in chlorinated solvents formed blue-green/brownish solutions showing the electronic absorption expected for the nonaromatic carbaporphyrinoids (Figure 3). The UV–vis spectrum of **2-Se** consists of intense bands with maxima at 309 (log ε = 4.3) and 378 nm (log ε = 4.6) and broad absorption in the 550–750 nm range centered at 661 nm (log ε = 4.0). The **3-Se** spectrum consists of intense absorption at 373 (log ε = 4.5) nm (sh. 388 nm) and lower intensity bands at 308 (log ε = 4.3), 523 (log ε = 3.8), and 682 (log ε = 3.8) nm. Similarly, **4-Se** showed strong absorption with maxima at 317 (log ε = 4.4), 374 (log ε = 4.6), and 391 (log ε = 4.5) nm, aside from the broad band in the visible range of 570–850 nm centered at 702 nm (log ε = 4.1).

**Figure 3:**
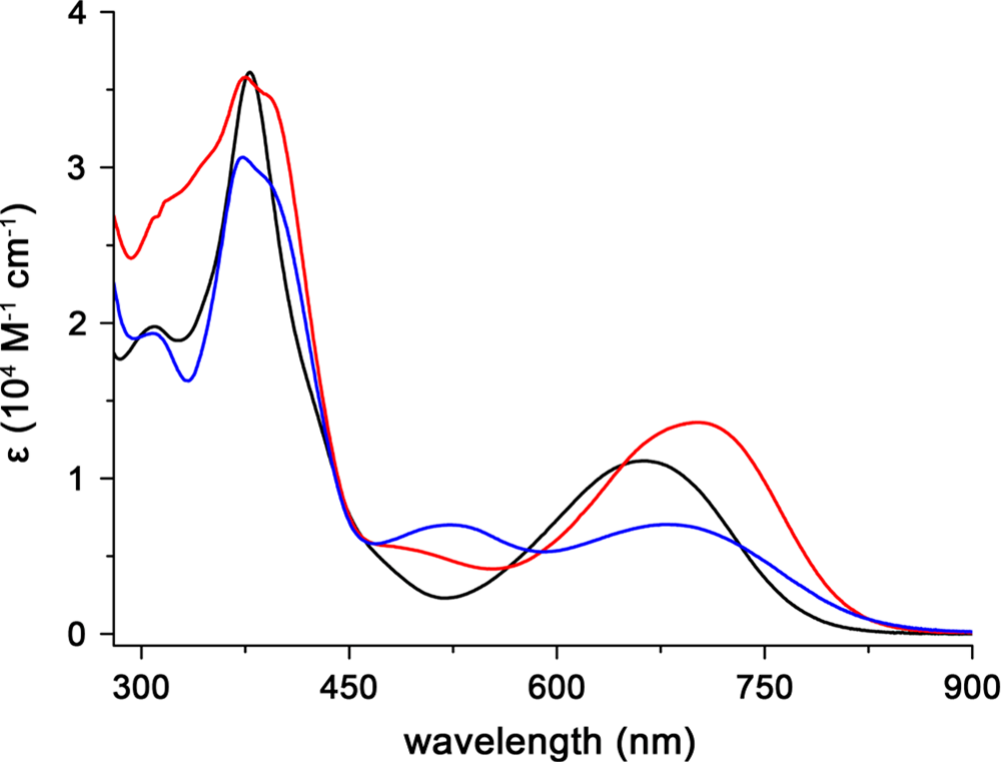
UV–vis electronic absorption spectra (298 K) of **2-Se** (black, CHCl_3_), **3-Se** (blue, DCM), and **4-Se** (red, CHCl_3_).

Although various amination reactions were previously reported for carbaporphyrinoids, they typically required the intermediacy of the transition metal coordination, activating the respective C–H bond toward the active agent.^20,38,39^ The plausible mechanistic pathways explaining the formation of **2-Se** and **3-Se** are similar, involving the nucleophilic attack of the enolate formed from acetaldehyde (**2-Se**) or diethylamine (**3-Se**) on the C7=C8 double bond followed by a single or a double dehydrogenation yielding the target products (Scheme S1, SI). In fact, acetaldehyde is formed from alkylamines under light/heat through a radical pathway.^40,41^ The mechanism of **4-Se** formation in the reaction of **1-Se** with TEA seems to be more elaborate. Although some of the individual steps, e.g., the formation of C_ethyl_–N27 and C_ethyl_–C26 bonds, seem similar to these occurring for **2-Se** and **3-Se**, the overall process is more complex. The observed selectivity might also suggest the intermediacy of amine radical cations in the reaction.^40^

The reactions of 28-thia- and 28-selena-2,7-naphthiporphyrins with triethylamine and diethylamine resulted in an unusual intracavity extension of the carbaporphyrinoid framework. The resultant macrocycles incorporating naphthodihydro-2*H*-pyran, naphthotetrahydropyridine, and naphthopyrrolotetrahydro-1*H*-azepine moieties demonstrated highly folded conformations, as determined in the solid state. The observed transformations of naphthiporphyrinoids carried out in the presence of simple alkylamines indicated that the choice of a base, typically required for preparation of carbaporphyrinoids coordination compounds or introduced as a proton scavenger, should be carefully evaluated, as in some instances its role in the observed transformations might be more elaborate than anticipated.

## Data Availability

The data underlying this study are available in the published article and its Supporting Information.

## Supporting Information Available

Experimental and spectroscopic details (PDF)

## References

[ref1] Lash T. D. Carbaporphyrinoid Systems. Chem. Rev. 2017, 117 (4), 2313–2446. 10.1021/acs.chemrev.6b00326. 27657332

[ref2] Szyszko B.; Latos-Grażyński L. Core Chemistry and Skeletal Rearrangements of Porphyrinoids and Metalloporphyrinoids. Chem. Soc. Rev. 2015, 44 (11), 3588–3616. 10.1039/C4CS00398E. 25875184

[ref3] Stępień M.; Sprutta N.; Latos-Grażyński L. Figure Eights, Möbius Bands, and More: Conformation and Aromaticity of Porphyrinoids. Angew. Chem., Int. Ed. 2011, 50 (19), 4288–4340. 10.1002/anie.201003353. 21495122

[ref4] Ke X.-S.; Kim T.; He Q.; Lynch V. M.; Kim D.; Sessler J. L. Three-Dimensional Fully Conjugated Carbaporphyrin Cage. J. Am. Chem. Soc. 2018, 140 (48), 16455–16459. 10.1021/jacs.8b11158. 30452259

[ref5] Berlicka A.; Stanowska J.; Białek M. J.; Ślepokura K.; Latos-Grażyński L. Dicarba[26]Hexaporphyrinoids(1.1.1.1.1.1) with an Embedded Cyclopentene Moiety—Conformational Switching. Chem. Eur. J. 2020, 26 (54), 12322–12327. 10.1002/chem.202002603. 32633431

[ref6] AbuSalim D. I.; Ferrence G. M.; Lash T. D. Synthesis of an *Adj* -Dicarbaporphyrin and the Formation of an Unprecedented Tripalladium Sandwich Complex. J. Am. Chem. Soc. 2014, 136 (18), 6763–6772. 10.1021/ja502795x. 24738618

[ref7] Furuta H.; Maeda H.; Osuka A. Doubly *N*-Confused Porphyrin: A New Complexing Agent Capable of Stabilizing Higher Oxidation States. J. Am. Chem. Soc. 2000, 122 (5), 803–807. 10.1021/ja992679g.

[ref8] Ke X.-S.; Hong Y.; Tu P.; He Q.; Lynch V. M.; Kim D.; Sessler J. L. Hetero Cu(III)–Pd(II) Complex of a Dibenzo[*g, p*]Chrysene-Fused Bis-Dicarbacorrole with Stable Organic Radical Character. J. Am. Chem. Soc. 2017, 139 (42), 15232–15238. 10.1021/jacs.7b09167. 28965390

[ref9] Ke X.-S.; Hong Y.; Lynch V. M.; Kim D.; Sessler J. L. Metal-Stabilized Quinoidal Dibenzo[*g, p*]Chrysene-Fused Bis-Dicarbacorrole System. J. Am. Chem. Soc. 2018, 140 (24), 7579–7586. 10.1021/jacs.8b02718. 29787675

[ref10] Lash T. D.; Young A. M.; Rasmussen J. M.; Ferrence G. M. Naphthiporphyrins. J. Org. Chem. 2011, 76 (14), 5636–5651. 10.1021/jo200622s. 21604773

[ref11] Szyszko B.; Pacholska-Dudziak E.; Latos-Grażyński L. Incorporation of the 1,5-Naphthalene Subunit into Heteroporphyrin Structure: Toward Helical Aceneporphyrinoids. J. Org. Chem. 2013, 78 (10), 5090–5095. 10.1021/jo4006624. 23611447

[ref12] Szyszko B.; Latos-Grażyński L. Conformational Flexibility of 1,4-Naphthiporphyrin Promotes a Palladium-Mediated Contraction of Naphthalene to Isoindene. Organometallics 2011, 30 (16), 4354–4363. 10.1021/om2004139.

[ref13] Stępień M.; Latos-Grażyński L. Tetraphenylbenziporphyrin—A Ligand for Organometallic Chemistry. Chem. - Eur. J. 2001, 7 (23), 5113–5117. 10.1002/1521-3765(20011203)7:23<5113::AID-CHEM5113>3.0.CO;2-V. 11775684

[ref14] Stȩpień M.; Latos-Grażyński L. Benziporphyrins: Exploring Arene Chemistry in a Macrocyclic Environment. Acc. Chem. Res. 2005, 38 (2), 88–98. 10.1021/ar040189+. 15709728

[ref15] Szyszko B.; Matviyishyn M.; Hirka S.; Pacholska-Dudziak E.; Białońska A.; Latos-Grażyński L. 28-Hetero-2,7-Naphthiporphyrins: Horizontal Expansion of the *m*-Benziporphyrin Macrocycle. Org. Lett. 2019, 21 (17), 7009–7014. 10.1021/acs.orglett.9b02587. 31423794

[ref16] Szyszko B.; Rymut P.; Matviyishyn M.; Białońska A.; Latos-Grażyński L. Kinetic versus Thermodynamic Control Over Multiple Conformations of Di-2,7-naphthihexaphyrin(1.1.1.1.1.1). Angew. Chem., Int. Ed. 2020, 59 (45), 20137–20146. 10.1002/anie.202008518. 33462869

[ref17] Chmielewski P. J.; Latos-Grażyński L.; Rachlewicz K.; Glowiak T. Tetra-*p*-Tolylporphyrin with an Inverted Pyrrole Ring: A Novel Isomer of Porphyrin. Angew. Chem., Int. Ed. Engl. 1994, 33 (7), 779–781. 10.1002/anie.199407791.

[ref18] Furuta H.; Asano T.; Ogawa T. “N-Confused Porphyrin”: A New Isomer of Tetraphenylporphyrin. J. Am. Chem. Soc. 1994, 116 (2), 767–768. 10.1021/ja00081a047.

[ref19] Białek M. J.; Hurej K.; Furuta H.; Latos-Grażyński L. Organometallic Chemistry Confined within a Porphyrin-like Framework. Chem. Soc. Rev. 2023, 52 (6), 2082–2144. 10.1039/D2CS00784C. 36852929

[ref20] Stȩpień M.; Latos-Grażyński L. Regioselective Pyridination of *m*-Benziporphyrin. Org. Lett. 2003, 5 (19), 3379–3381. 10.1021/ol035232s. 12967279

[ref21] Szyszko B.; Kupietz K.; Szterenberg L.; Latos-Grażyński L. Gold(III)-Mediated Contraction of Benzene to Cyclopentadiene: From *p*-Benziporphyrin to Gold(III) True Tetraarylcarbaporphyrin. Chem. - Eur. J. 2014, 20 (5), 1376–1382. 10.1002/chem.201304162. 24382653

[ref22] Hurej K.; Pawlicki M.; Szterenberg L.; Latos-Grażyński L. A Rhodium-Mediated Contraction of Benzene to Cyclopentadiene: Transformations of Rhodium(III) *m*-Benziporphyrin. Angew. Chem., Int. Ed. 2016, 55 (4), 1427–1431. 10.1002/anie.201508033. 26643286

[ref23] Swift E. The Densities of Some Aliphatic Amines. J. Am. Chem. Soc. 1942, 64 (1), 115–116. 10.1021/ja01253a030.

[ref24] Armarego W. L. F.; Chai C. L. L. Purification of Laboratory Chemicals, 6th ed.; Elsevier: Oxford, UK, 2009.

[ref25] Roose P.; Eller K.; Henkes E.; Rossbacher R.; Höke H. Amines, Aliphatic. In Ullmann’s Encyclopedia of Industrial Chemistry; Wiley, 2015; pp 1–55.

[ref26] Orpen A. G.; Brammer L.; Allen F. H.; Kennard O.; Watson D. G.; Taylor R. Appendix A: Typical Interatomic Distances in Organic Compounds and Organometallic Compounds and Coordination Complexes of the D- and F-block Metals. In Structure Correlation; Bürgi H., Dunitz J. D., Eds.; Wiley, 1994; pp 752–858.

[ref27] Bacchi A.; Costa M.; Della Cà N.; Fabbricatore M.; Fazio A.; Gabriele B.; Nasi C.; Salerno G. Synthesis of 1-(Alkoxycarbonyl)Methylene-1,3-Dihydroisobenzofurans and 4-(Alkoxycarbonyl)Benzo[c]Pyrans by Palladium-Catalysed Oxidative Carbonylation of 2-Alkynylbenzyl Alcohols, 2-Alkynylbenzaldehydes and 2-Alkynylphenyl Ketones. Eur. J. Org. Chem. 2004, 2004 (3), 574–585. 10.1002/ejoc.200300577.

[ref28] Terada M.; Li F.; Toda Y. Chiral Silver Phosphate Catalyzed Transformation of *Ortho* -Alkynylaryl Ketones into 1 *H* -Isochromene Derivatives through an Intramolecular-Cyclization/Enantioselective-Reduction Sequence. Angew. Chem., Int. Ed. 2014, 53 (1), 235–239. 10.1002/anie.201307371. 24273203

[ref29] Ackermann M.; Bucher J.; Rappold M.; Graf K.; Rominger F.; Hashmi A. S. K. [3,3]-Sigmatropic Rearrangement Step in the Gold-Catalyzed Cyclization of Allyl-(*Ortho* -Alkinylphenyl)Methyl Ethers. Chem. - Asian J. 2013, 8 (8), 1786–1794. 10.1002/asia.201300324. 23729410

[ref30] Nishinaga A.; Shimizu T.; Toyoda Y.; Matsuura T.; Hirotsu K. Oxygenation of 2,6-Di-Tert-Butylphenols Bearing an Electron-Withdrawing Group in the 4-Position. J. Org. Chem. 1982, 47 (12), 2278–2285. 10.1021/jo00133a009.

[ref31] Chen S.-J.; Zhu H.; Zhang M.-M.; Xu W.-W.; Wang Y.-C.; Zhang Z.-F. CCDC 1876613: Experimental Crystal Structure Determination. Access Structures. Cambridge Crystallographic Data Centre, deposited January 14, 2019. 10.5517/ccdc.csd.cc20zrxp.

[ref32] Furuta H.; Ishizuka T.; Osuka A.; Ogawa T. N-Fused Porphyrin” from *N*-Confused Porphyrin. J. Am. Chem. Soc. 1999, 121 (12), 2945–2946. 10.1021/ja9902672.

[ref33] Abraham J. A.; Mori S.; Ishida M.; Furuta H. Synthesis and Characterization of N-Fused Porphyrin Rhodium Complex with an Isomerized Cyclooctadiene Ligand. Chem. Lett. 2021, 50 (9), 1707–1709. 10.1246/cl.210381.

[ref34] Ikeda S.; Toganoh M.; Furuta H. Synthesis, Reactivity, and Properties of *N*-Fused Porphyrin Manganese(I) Tricarbonyl Complexes. Inorg. Chem. 2011, 50 (13), 6029–6043. 10.1021/ic2000393. 21657208

[ref35] Kumar S.; Rajeswara Rao M.; Ravikanth M. Stable Core-Modified Doubly *N*-Fused Expanded Dibenziporphyrinoids. J. Org. Chem. 2018, 83 (3), 1584–1590. 10.1021/acs.joc.7b02851. 29297224

[ref36] Młodzianowska A.; Latos-Grażyński L.; Szterenberg L. Phosphorus Complexes of *N*-Fused Porphyrin and Its Reduced Derivatives: New Isomers of Porphyrin Stabilized via Coordination. Inorg. Chem. 2008, 47 (14), 6364–6374. 10.1021/ic800437y. 18576597

[ref37] Sengupta R.; Isar P.; Ravikanth M. Synthesis of *N*-Fused Dithia and Dibenzi Homoporphyrins. Org. Chem. Front. 2022, 9 (6), 1580–1588. 10.1039/D1QO01946E.

[ref38] Ren D.; Liu B.; Li X.; Koniarz S.; Pawlicki M.; Chmielewski P. J. Reactions of 2-Aza-21-Carbaporphyrin with Aniline Derivatives. Org. Chem. Front. 2019, 6 (7), 908–918. 10.1039/C9QO00024K.

[ref39] Grzegorzek N.; Pawlicki M.; Latos-Grażyński L. Regioselective Amination of Carbaporpholactone and *N*-Confused Porphyrin. J. Org. Chem. 2009, 74 (22), 8547–8553. 10.1021/jo901508v. 19860402

[ref40] Hu J.; Wang J.; Nguyen T. H.; Zheng N. The Chemistry of Amine Radical Cations Produced by Visible Light Photoredox Catalysis. Beilstein J. Org. Chem. 2013, 9, 1977–2001. 10.3762/bjoc.9.234. 24204409 PMC3817571

[ref41] Cullis C. F.; Waddington D. J. The Gaseous Oxidation of Tertiary Aliphatic Amines - I. Triethylamine. Proc. R. Soc. Lond. A 1958, 244 (1236), 110–123. 10.1098/rspa.1958.0029.

