# Supplementary material for: The Intracavity Extension of 28-Hetero-2,7-naphthiporphyrins in Reactions with Alkylamines
Source: Org Lett. 2023 Jun 22;25(25):4735–9. doi: 10.1021/acs.orglett.3c01715 (PMC10324391; doi:10.1021/acs.orglett.3c01715)
Supplement: Supplementary file 1 — ol3c01715_si_001.pdf [file ol3c01715_si_001.pdf]

The Intracavity Extension of 28-Hetero-2,7-naphthiporphyrins in the Reactions with Alkylamines

by Katarzyna Ślusarek‡, Jędrzej P. Perdek‡, Agata Białońska, Rafał A. Grzelczak, and Bartosz Szyszko*

University of Wrocław, Faculty of Chemistry, 14 F. Joliot-Curie St., 50-383 Wrocław, Poland

Supporting Information

Table of Contents

Table of Contents 2

Instrumentation 3

Synthesis 5

NMR spectra 12

NMR spectra of 2,5-bis(tolylhydroxymethyl)thiophene **S1** 12

NMR spectra of 8,23-diphenyl-13,18-ditolyl-28-thia-2,7-naphthiporphyrin **1-S** 13

NMR spectra of macrocycle **2-Se** 15

NMR spectra of macrocycle **3-Se** 29

NMR spectra of macrocycle **4-Se** 43

NMR spectra of macrocycle **4-S** 59

NMR spectra of crude reaction mixtures 65

High-resolution mass spectra 69

UV-Vis absorption spectra 72

References 75

Instrumentation

NMR Spectroscopy

^1H and ^{13}C NMR spectra were recorded on a high-field Bruker spectrometers (^1H 600.15 MHz and 500.13 MHz), equipped with a broadband inverse gradient probe head, and a high-field- JEOL (^1H 500 MHz) spectrometer, equipped with a 5 mm wide broadband probe. Spectra were referenced to the residual solvent signal (chloroform-*d* – 7.24 ppm, dichloromethane-d_2 – 5.32 ppm). Two-dimensional NMR spectra were recorded with 2048 data points in the t_2 domain and up to 1024 points in the t_1 domain, with a 1 s recovery delay. Structural assignments were made with additional information from gCOSY, gHSQC, and gHMBC experiments.

Mass Spectrometry

The ESI mass spectra were recorded on Bruker qTOF compact and Bruker Q-TOF-MS/MS maXis impact spectrometers.

UV-Vis spectroscopy

Electronic spectra were recorded on a Varian Cary-50 Bio, and Varian Cary-60 spectrophotometers.

The X-ray diffraction data.

Monocrystals suitable for the XRD analysis were obtained by slow evaporation of dichloromethane/*n*-hexane solution **2-Se**, chloroform/*n*-hexane solution of **3-Se**, and chloroform/methanol solution of **4-Se**. Single-crystal X-ray diffraction data for **2-Se**, **3-Se,** and **4-Se** were collected at 100 K on XtaLAB Synergy R, DW system (HyPix-Arc 150) κ-geometry diffractometer using *Cu* K*α* radiation. Data reduction and analysis were carried out with CrysAlis Pro.[1] The structures were solved by direct methods and refined with the full-matrix least-squares technique using the *SHELXS*[2] and *Shelxl-2018/3*[3] programs. Hydrogen atom bonded to the nitrogen atom in **2-Se** and in **3-Se** was found in the Dr map, and hydrogen atoms bonded to the carbon atoms in **2-Se**, **3-Se**, and **4-Se** were placed at calculated positions. Before the last refinement cycle, all H atoms were fixed and allowed to ride on their parent atoms. Anisotropic displacement parameters were refined for all non-hydrogen atoms.

Crystal data for compound **2-Se**: $C_{54}H_{38}N_2OSe$, C_6H_{14}, M = 895.99, monoclinic, $P2_1/c$, a = 19.8162(8) Å, b = 15.7450(4) Å, c = 15.3860(5) Å, β = 107.718(4)°, V = 4572.8(3) Å^3, Z = 4, D_c = 1.301 Mg m^{-3}, T = 100.00(10) K, R = 0.0769, wR = 0.2011 (6093 reflections with $I > 2\sigma(I)$) for 577 variables, CCDC 2263149.

Crystal data for compound **3-Se**: $C_{56}H_{43}N_3Se$, M = 836.89, triclinic, P-1, a = 8.6811(5) Å, b = 14.9849(8) Å, c = 16.6190(8) Å, α = 85.640(4)°, β = 75.357(5)°, γ = 81.981(5)°, V = 2069.3(2) Å^3, Z = 2, D_c = 1.343 Mg m^{-3}, T = 100.00(10) K, R = 0.0531, wR = 0.1330 (7733 reflections with $I > 2\sigma(I)$) for 541 variables, CCDC 2263150.

Crystal data for compound **4-Se**: $C_{58}H_{47}N_3Se$, M = 864.94, monoclinic, Cc, a = 14.607(3) Å, b = 18.898(3) Å, c = 16.084(3) Å, β = 93.74(2)°, V = 4430.4(14) Å^3, Z = 4, D_c = 1.297 Mg m^{-3}, T = 100.00(10) K, R = 0.0337, wR = 0.0947 (6656 reflections with $I > 2\sigma(I)$) for 560 variables, CCDC 2263151.

Synthesis

Solvents and reagents

Dichloromethane, chloroform, chloroform-*d*, and dichloromethane-d_2 were passed through a column of basic alumina directly before use. Pyrrole was distilled from calcium hydride and passed through a column of basic alumina directly before use. Basic alumina was deactivated by mixing with demineralized water (4 g H_2O per 100 g of basic alumina). Diethylamine and triethylamine were purified according to the procedures described below. Reagents not listed here were used without purification.

Purified triethylamine (TEA)[4]

In a 250 mL round-bottomed flask equipped with a magnetic stirrer, benzoic anhydride (1 g, 4 mmol), phthalic anhydride (1 g, 7 mmol), and acetic anhydride (1 mL, 10 mmol) were placed, and 150 mL of triethylamine (≥99.5%) was introduced. The mixture was refluxed for 1 hour using a heating mantle. The solution was distilled off and subsequently refluxed with calcium hydride (1 g, 24 mmol) for 1 hour. After this time, triethylamine was separated by fractional distillation. The fraction boiling at 89.0 °C ± 0.5 °C was collected and passed through a column of basic alumina. The reagent was used directly after purification.

Purified diethylamine (DEA)[5,6]

In a 500 mL two-neck round-bottomed flask, 4-methylbenzenesulfonyl chloride (95 g, 0.5 mol) and triethylamine (52 mL, 0.5 mol) were dissolved in 200 mL of dichloromethane. The solution was cooled down to 0˚C in an ice bath, and diethylamine (≥99.5%, 100 mL, 1 mol) was added dropwise from the addition funnel. The solution was stirred at room temperature for 2 hours. The solvents were removed on a rotary evaporator under reduced pressure. The off-white crystalline solid has been recrystallized three times from the EtOH/*n*-hexane mixture. The solid has been placed in a 500 mL round-bottomed flask with 250 mL of concentrated hydrochloric acid. The mixture was refluxed for 96 hours using an oil bath. After this time, the solution was concentrated by distilling off the solvent, and sodium hydroxide solution was slowly added until the solution was alkalized. Diethylamine was separated by fractional distillation. The fraction boiling at 55.5 ± 0.5 ˚C was collected. It was next

distilled from KOH pellets and passed through a column of basic alumina. The reagent was used directly after purification.

2,5-Bis(tolylhydroxymethyl)thiophene S1 was synthesized as described in the literature.[7] The compound was obtained as a white amorphous powder, as described in the original procedure.

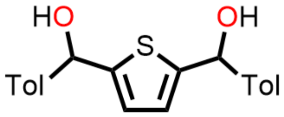

S1

The previously not-reported analytical data are provided below.
^{1}H NMR (chloroform-*d*, 300 K, 500 MHz, mixture of diastereoisomers): δ 7.29 (d, 4H, ^3J = 8.0 Hz, *o*-Tol), 7.14 (d, 4H, ^3J = 7.9 Hz, *m*-Tol), 6.68 (and 6.67, s, 2H, *β*-thiophene), 5.92 (s, 2H, *meso*), 2.33 (s, 6H, CH_3-Tol).
^{13}C NMR (chloroform-*d*, 300 K, 126 MHz, mixture of diastereoisomers): δ 148.3 (and 148.2), 140.2, 137.67 (and 137.65), 129.2, 126.4 (and 126.3), 124.4 (and 124.3), 72.4 (and 72.3), 21.2.
HRMS (ESI+, MS): *m/z* $[M-OH]^+$: 307.1161, calcd. for $C_{20}H_{19}OS^+$: 307.1151.

Naphthalene-2,7-dicarbaldehyde was synthesized as described in the literature.[8]

2,5-Bis(tolylhydroxymethyl)selenophene was synthesized as described in the literature.[9]

2,7-Bis(phenylhydroxymethyl)naphthalene was synthesized as described in the literature.[10]

2,7-Bis(phenyl(2-pyrrolyl)methyl)naphthalene was synthesized as described in the literature.[10]

8,23-Diphenyl-13,18-ditolyl-28-selena-2,7-naphthiporphyrin 1-Se was synthesized as described in the literature.[10]

8,23-Diphenyl-13,18-ditolyl-28-thia-2,7-naphthiporphyrin 1-S.

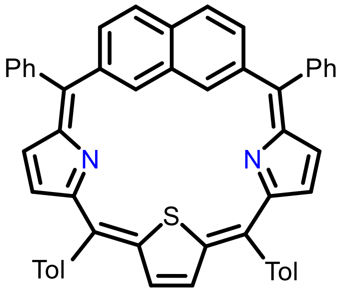

1-S

In a 250 mL round-bottomed flask equipped with a magnetic stirrer, 2,7-bis(phenyl(2-pyrrolyl)methyl)naphthalene **S5** (110 mg, 0.25 mmol) and 2,5-bis(tolylhydroxymethyl)-thiophene **S1** (82 mg, 0.25 mmol) were introduced and dissolved in 150 mL of dichloromethane which was passed through a column of basic alumina directly before reaction. The solution was purged with nitrogen gas for 20 minutes and 26 µL of boron trifluoride diethyl etherate (BF_3:Et_2O) was added and the flask was covered from light. The solution was stirred under nitrogen for 2 hours. 2,3-Dichloro-5,6-dicyano-1,4-benzoquinone (DDQ, 142 mg, 0.65 mmol) was added and the solution was stirred for another 15 minutes. The solvent was removed on a rotary evaporator under reduced pressure. The initial chromatography was performed on deactivated basic alumina (eluent DCM). The product eluted in the first fraction, which was consequently purified by flash chromatography on a silica gel column (Büchi Flash Pure Silica 12 g), with 5% ethyl acetate in DCM as the eluent with the flow rate of 30 mL per minute. The product **1-S** eluted as the second dark green fraction. Yield 27 mg (12%).

^{1}H NMR (*chloroform-d*, 300 K, 500 MHz): δ 9.79 (m, 2H, C24-H, C26-H), 7.65 (d, 2H, ^3J = 8.7 Hz, C3-H, C5-H), 7.51 – 7.48 (m, 4H, 8-*o*-Ph, 23-*o*-Ph), 7.48 – 7.39 (m, 6H, 8-*m*,*p*-Ph, 23-*m*,*p*-Ph), 7.34 (dd, 2H, ^3J = 8.6 Hz, ^4J = 1.7 Hz, C2-H, C6-H), 7.19 (s, 8H, 13-Tol, 18-Tol), 6.92 (d, 2H, ^3J = 4.7 Hz, C10-H, C21-H), 6.34 (s, 2H, C15-H, C16-H), 6.30 (d, 2H, ^3J = 4.7 Hz, C11-H, C20-H), 2.39 (s, 6H, 13-CH_3-Tol, 18-CH_3-Tol).

^{13}C NMR (*chloroform-d*, 300 K, 151 MHz): δ 170.9, 157.5, 150.8, 150.2, 142.2, 140.5, 138.4, 137.6, 137.1, 136.9, 135.9, 133.6, 132.5, 132.1, 131.1, 131.0, 130.6, 129.4, 129.1, 128.4, 126.0, 21.4.

HRMS (ESI+, MS): *m/z* $[M+H]^+$: 721.2708, *m/z* calcd. for $C_{52}H_{37}N_2S^+$: 721.2672.

UV-vis (DCM, 298 K): λ_{max} (log (ε)) 365 (4.6), 440 (4.4), 661 (3.9).

Synthesis of 2-Se, 3-Se, and 4-Se

To exclude the metal catalyst involvement in the formation of **2-Se**, **3-Se**, **4-Se**, and **4-S**, a set of new magnetic stirring bars were exploited for the reactions, which were additionally treated with *aqua regia* prior to the reaction. The procedures described below have also been successfully carried out using glass stirring bars.

Macrocycle 2-Se.

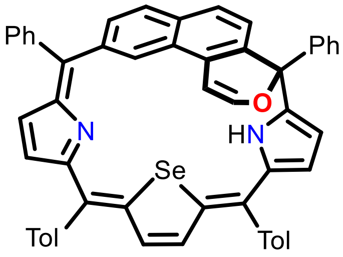

2-Se

In a 100 mL round-bottomed flask equipped with a magnetic stirrer, 8,23-diphenyl-13,18-ditolyl-28-selena-2,7-naphthiporphyrin **1-Se** (30 mg, 0.04 mmol) was dissolved in 40 mL of commercially available diethylamine or triethylamine. The solution was refluxed for 24 hours using an oil bath. Then, the solvent was removed on a rotary evaporator under reduced pressure. The initial column chromatography was performed on a silica gel column using 2% EtOAc in DCM as eluent. The product eluted in the fourth brown fraction and was subsequently chromatographed on a silica gel column using 5% EtOAc in *n*-hexane as eluent. The obtained **2-Se** was then recrystallized from MeCN:DCM (1:1) mixture. Yield 14 mg (45%) from diethylamine; 11 mg (34%) from triethylamine.

^{1}H NMR (*chloroform-d*, 300 K, 600 MHz): δ 9.73 (s, 1H, C24-H), 9.69 (s, 1H, N27-H), 7.73 (m, 2H, 8-*o*-Ph), 7.68 (d, 1H, ^{3}J = 8.3 Hz, C3-H), 7.55 (d, 1H, ^{3}J = 8.7 Hz, C5-H), 7.50-7.45 (m, 3H, C30-H, 8-*m*-Ph), 7.45-7.37 (m, 6H, 8-*p*-Ph, 23-Ph), 7.28 (d, 1H, ^{3}J = 5.8 Hz, C31-H), 7.18-7.11 (m, 5H, C2-H, 18-Tol), 7.11 – 7.08 (m, 2H, 13-*m*-Tol), 7.08-7.04 (m, 3H, 13-*o*-Tol, C21-H), 6.72 (d, 1H, ^{3}J = 8.5 Hz, C6-H), 6.34 (d, 1H, ^{3}J = 4.8 Hz, C20-H), 6.29 (d, 1H, ^{3}J = 6.4 Hz, C15-H), 6.16 (d, 1H, ^{3}J = 6.4 Hz, C16-H), 5.89 (d, 1H, ^{3}J = 3.9 Hz, C10-H), 5.61 (d, 1H, ^{3}J = 3.5 Hz, C11-H), 2.35 (s, 3H, CH_3-18-Tol), 2.33 (s, 3H, CH_3-13-Tol).

^{13}C NMR (chloroform-*d*, 300 K, 151 MHz): δ 172.6, 157.6, 150.3, 149.1, 148.2, 140.6, 140.5, 139.8, 138.6, 138.13, 138.10, 138.0, 137.5, 137.4, 137.3, 135.4, 134.0, 133.6, 132.6, 132.44, 132.38, 130.9, 130.5, 129.7, 129.4, 129.3, 129.2, 128.7, 128.5, 128.29, 128.26, 128.09, 128.05, 127.6, 126.2, 126.1, 125.1, 117.5, 110.8, 102.9, 82.1, 21.40, 21.38.

HRMS (ESI, TOF): *m/z* $[M+H]^+$: 811.2205, calcd. for $C_{54}H_{39}N_2OSe^+$: 811.2228.

UV-vis ($CHCl_3$, 298 K): λ_{max} (log (ε)) 309 (4.3), 378 (4.6), 661 (4.0).

Macrocycle 3-Se.

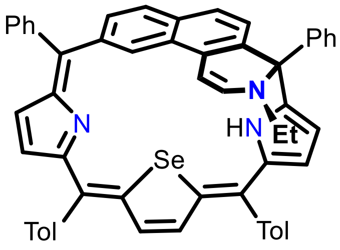

3-Se

In a 100 mL round-bottomed flask equipped with a magnetic stirrer, 8,23-diphenyl-13,18-ditolyl-28-selena-2,7-naphthiporphyrin **1-Se** (30 mg, 0.04 mmol) was dissolved in 40 mL of commercially available diethylamine. The solution was stirred at room temperature for 24 hours. Then, the solvent was removed on rotary evaporator under reduced pressure. The initial column chromatography was performed on silica gel column using 2% EtOAc in DCM as eluent. The product eluted in the fourth brown fraction and was subsequently chromatographed on silica gel column using 10% EtOAc in *n*-hexane as eluent. The obtained **3-Se** was then recrystallized from *n*-hexane:DCM (1:1) mixture. Yield 15 mg (45%).

^{1}H NMR (*chloroform-d*, 300 K, 600 MHz): δ 10.40 (s, 1H, N27-H), 10.07 (s, 1H, C24-H), 7.89 (s, 2H, 8-*o*-Ph), 7.58 (d, 1H, ^{3}J = 8.5 Hz, C3-H), 7.45 (t, 2H, 8-*m*-Ph), 7.40 – 7.32 (m, 6H, 8-*p*-Ph, 23-Ph), 7.28 (d, 1H, ^{3}J = 8.4 Hz, C5-H), 7.19 (m, 1H, ^{3}J = 7.7 Hz, C30-H), 7.14 (b, 4H, 18-*o,m*-Tol), 7.08 (d, 2H, ^{3}J = 7.8 Hz, 13-*m*-Tol), 7.02 (m, 3H, 2-H, 13-*o*-Tol), 6.98 (d, 1H, ^{3}J = 7.9 Hz, C31-H), 6.97 (d, 1H, ^{3}J = 4.7 Hz, C21-H), 6.31 (d, 1H, ^{3}J = 8.8 Hz, C6-H), 6.28 (d, 1H, ^{3}J = 4.7 Hz, C20-H), 6.19 (d, 1H, ^{3}J = 6.4 Hz, C15-H), 6.08 (d, 1H, ^{3}J = 6.4 Hz, C16-H), 5.83 (m, 1H, C10-H), 5.55 (m, 1H, C11-H), 3.00 – 2.90 (m, 1H, CH_2-NEt), 2.88 – 2.80 (m, 1H, CH_2-NEt), 2.34 (s, 3H, 18-CH_3-Tol), 2.32 (s, 3H, 13-CH_3-Tol), 1.09 (t, 3H, ^{3}J = 7.2 Hz, CH_3-NEt).

^{13}C NMR (chloroform-*d*, 300 K, 151 MHz) δ (ppm): 172.4, 157.7, 151.2, 149.3, 142.7, 140.8, 139.7, 138.5, 138.3, 138.1, 137.7, 137.3, 137.2, 137.1, 135.4, 134.0, 133.0, 132.7, 132.4, 132.2, 131.3, 130.9, 130.71, 130.69, 130.5, 130.3, 129.4, 129.3, 129.1, 128.9, 128.7, 128.2, 128.0, 127.8, 127.7, 126.4, 125.7, 125.3, 117.3, 108.0, 95.6, 69.6, 21.4, 14.4, 14.3.

HRMS (ESI, TOF): *m/z* $[M+H]^{+}$: 838.2725, calcd. for $C_{56}H_{44}N_3Se^{+}$: 838.2702.

UV-vis (DCM, 298 K): λ_{max} (log (ε)) 308 (4.3), 373 (4.5), 523 (3.8), 682 (3.8).

Macrocycle 4-Se

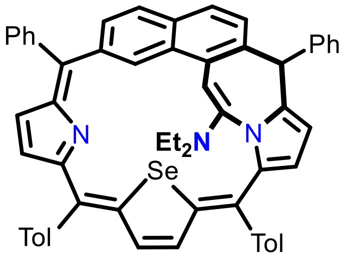

4-Se

In a 100 mL round-bottomed flask equipped with a magnetic stirrer, 8,23-diphenyl-13,18-ditolyl-28-selena-2,7-naphthiporphyrin **1-Se** (30 mg, 0.04 mmol) was dissolved in 40 mL of purified triethylamine. The solution was refluxed for 24 hours using an oil bath. Then, the solvent was removed on a rotary evaporator under reduced pressure. The initial column chromatography was performed on a silica gel column using 2% EtOAc in DCM as eluent. The product eluted in the second green fraction and was subsequently chromatographed on a silica gel column using 5% EtOAc in *n*-hexane as eluent. The crude **4-Se** was then recrystallized from MeCN:DCM (1:1) mixture. Yield 17 mg (51%).

^{1}H NMR (dichloromethane-d_2, 220 K, 600 MHz): δ 10.30 (s, 1H, C24-H), 7.66 (d, 2H, ^{3}J = 7.3 Hz, 8-*o*-Ph), 7.58 (d, 1H, ^{3}J = 8.6, C3-H), 7.53 (t, 2H, ^{3}J = 7.7 Hz, 8-*m*-Ph), 7.49 (d, 1H, ^{3}J = 8.7 Hz, C5-H), 7.46 – 7.28 (m, 6H, 8-*p*-Ph, 23-Ph), 7.25 (d, 1H, ^{3}J = 8.7 Hz, C6-H), 7.21 (m, 2H, 18-*m*-Tol), 7.14 (m, 2H, 18-*o*-Tol), 7.10 (m, 4H, 13-*o*,*m*-Tol), 6.91 (dd, 1H, ^{3}J = 8.5 Hz, ^{4}J = 1.8 Hz, C2-H), 6.83 (d, 1H, ^{3}J = 4.8 Hz, C21-H), 6.72 (b, 1H, C30-H), 6.51 (d, 1H, ^{3}J = 6.3 Hz, C15-H), 6.42 (d, 1H, ^{3}J = 6.3 Hz, C16-H), 6.30 (d, 1H, ^{3}J = 4.8 Hz, C20-H), 6.00 (d, 1H, ^{3}J = 3.9 Hz, C10-H), 5.70 (d, 1H, ^{3}J = 4.0, C11-H), 5.14 (b, 1H, C8-H), 3.82 (m, 1H, CH_2-NEt_2), 3.18 (m, 1H, CH_2-NEt_2), 2.91 (m, 1H, CH_2-NEt_2), 2.43 (m, 1H, CH_2-NEt_2), 2.36 (s, 3H, 18-CH_3-Tol), 2.32 (s, 3H, 13-CH_3-Tol), 0.96 – 0.88 (m, 6H, CH_3-NEt_2).

^{13}C NMR (chloroform-*d*, 300 K, 151 MHz) δ (ppm): 171.9, 156.7, 150.6, 149.4, 147.3, 146.1, 144.2, 142.4, 142.1, 141.2, 140.1, 137.8, 137.6, 137.4, 137.3, 136.9, 136.6, 134.9, 133.6, 132.21, 132.15, 132.1, 131.9, 131.8, 131.3, 130.7, 129.8, 129.2, 129.1, 128.7, 128.49, 128.46, 127.9, 127.6, 127.2, 126.9, 126.2, 122.5, 120.2, 105.9, 99.6, 49.4, 21.42, 21.39.

HRMS (ESI, TOF): *m/z* $[M+H]^+$: 866.3059, calcd. for $C_{58}H_{48}N_3Se^+$: 866.3015.

UV-vis ($CHCl_3$, 298 K): λ_{max} (log (ε)) 317 (4.4), 374 (4.6), 391 (4.5), 702 (4.1).

Macrocycle 4-S.

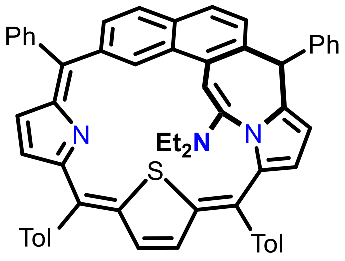

4-S

In a 100 mL round-bottomed flask equipped with a magnetic stirrer, 8,23-diphenyl-13,18-ditolyl-28-thia-2,7-naphthiporphyrin **1-S** (30 mg, 0.04 mmol) was dissolved in 40 mL of purified triethylamine. The solution was stirred at room temperature for 24 hours. Then, the solvent was removed on rotary evaporator under reduced pressure. The initial column chromatography was performed on silica gel column using 5% EtOAc in DCM as eluent. The product eluted in the fourth green fraction and was subsequently chromatographed on silica gel column using 10% EtOAc in *n*-hexane as eluent. The obtained **4-S** was then recrystallized from mixture of MeCN:DCM (1:1) mixture. Yield 15 mg (43%).

^{1}H NMR (chloroform-*d*, 300 K, 500 MHz): δ 10.34 (s, 1H, C24-H), 7.67 (m, 2H, 8-*o*-Ph), 7.56 – 7.49 (m, 3H, C3-H, 8-*m*-Ph), 7.47 (d, 1H, ^{3}J = 8.7 Hz, C5-H), 7.45 – 7.35 (m, 6H, 8-*p*-Ph, 23-Ph), 7.26 (d, 1H, ^{3}J = 8.6 Hz, C6-H), 7.21 – 7.09 (m, 6H, 13-*o*-Tol, 18-*o,m*-Tol), 7.06 (m, 2H, 13-*m*-Tol), 6.96 (dd, 1H, ^{3}J = 8.6 Hz, ^{4}J = 1.4 Hz, C2-H), 6.79 (d, 1H, ^{3}J = 4.7 Hz, C21-H), 6.72 (b, 1H, C30-H), 6.42 (d, 1H, ^{3}J = 5.9 Hz, C15-H), 6.32 (d, 1H, ^{3}J = 5.9 Hz, C16-H), 6.30 (d, 1H, ^{3}J = 4.7 Hz, C20-H), 6.02 (d, 1H, ^{3}J = 4.0 Hz, C10-H), 5.75 (d, 1H, ^{3}J = 4.0 Hz, C11-H), 5.24 (b, 1H, C8-H), 3.06 (b, 4H, CH_2-NEt_2), 2.38 (s, 3H, 18-CH_3-Tol), 2.34 (s, 3H, 13-CH_3-Tol), 0.97 (t, 6H, ^{3}J = 7.0 Hz, CH_3-NEt_2).

^{13}C NMR (chloroform-*d*, 300 K, 126 MHz): 171.0, 157.4, 150.2, 149.5, 146.9, 145.9, 143.9, 142.2, 141.9, 141.6, 138.4, 137.84, 137.80, 137.6, 136.9, 136.7, 136.5, 134.3, 133.7, 132.5, 132.4, 132.15, 132.07, 131.8, 131.2, 130.3, 129.9, 129.1, 129.0, 128.7, 128.5, 128.2, 127.9, 127.6, 126.9, 126.1, 123.9, 122.3, 120.5, 105.8, 99.2, 49.4, 29.8, 21.41, 21.39.

HRMS (ESI, TOF): *m/z* $[M+H]^+$: 818.3582, *m/z* calcd. for $C_{58}H_{48}N_3S^+$: 818.3569.

UV-vis (CH_2Cl_2, 298 K): λ_{max} (log (ε)) 321 (4.2), 373 (4.3), 391 (4.5), 695 (3.8).

NMR spectra

NMR spectra of 2,5-bis(tolylhydroxymethyl)thiophene S1

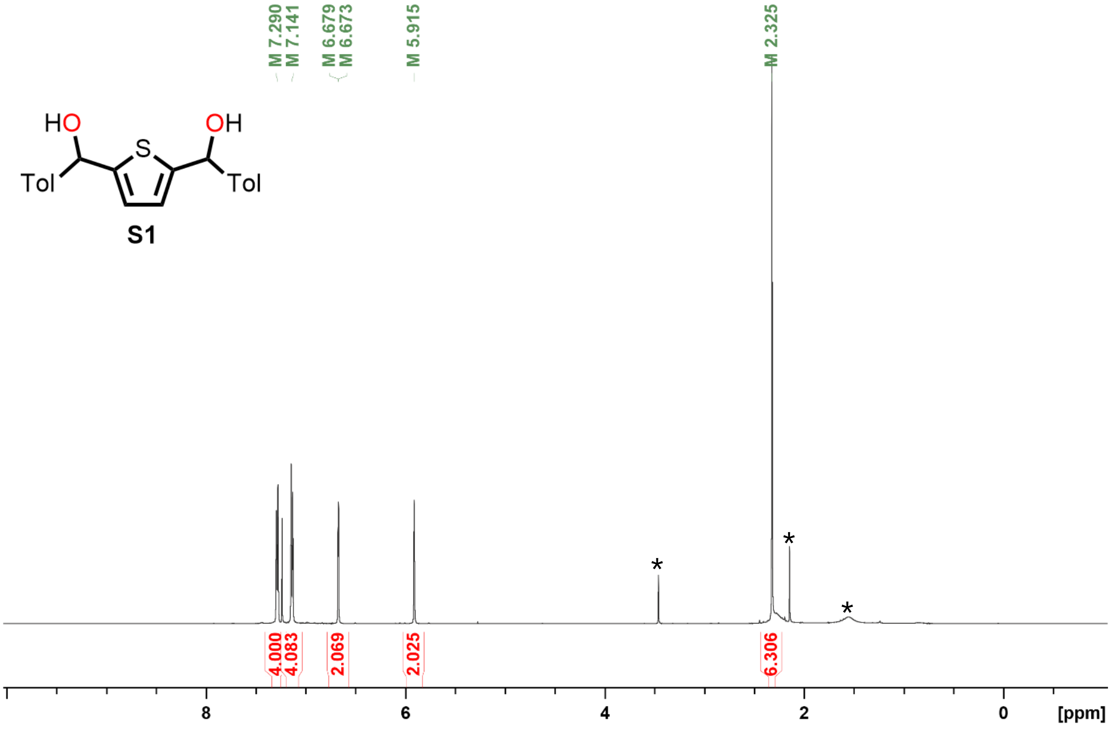

Figure S 1. The ^{1}H NMR spectrum of **S1** (500 MHz, chloroform-*d*, 300 K). Impurities were marked with asterisks.

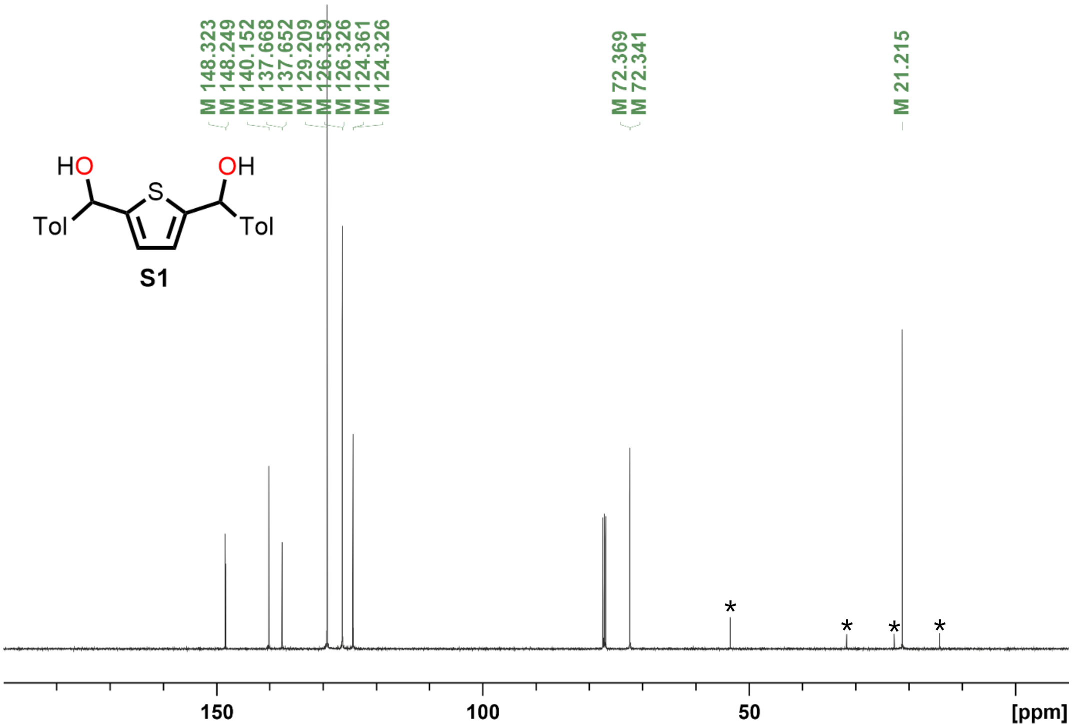

Figure S 2. The ^{13}C NMR spectrum of **S1** (126 MHz, chloroform-*d*, 300 K). Impurities were marked with asterisks.

NMR spectra of 8,23-diphenyl-13,18-ditolyl-28-thia-2,7-naphthiporphyrin 1-S

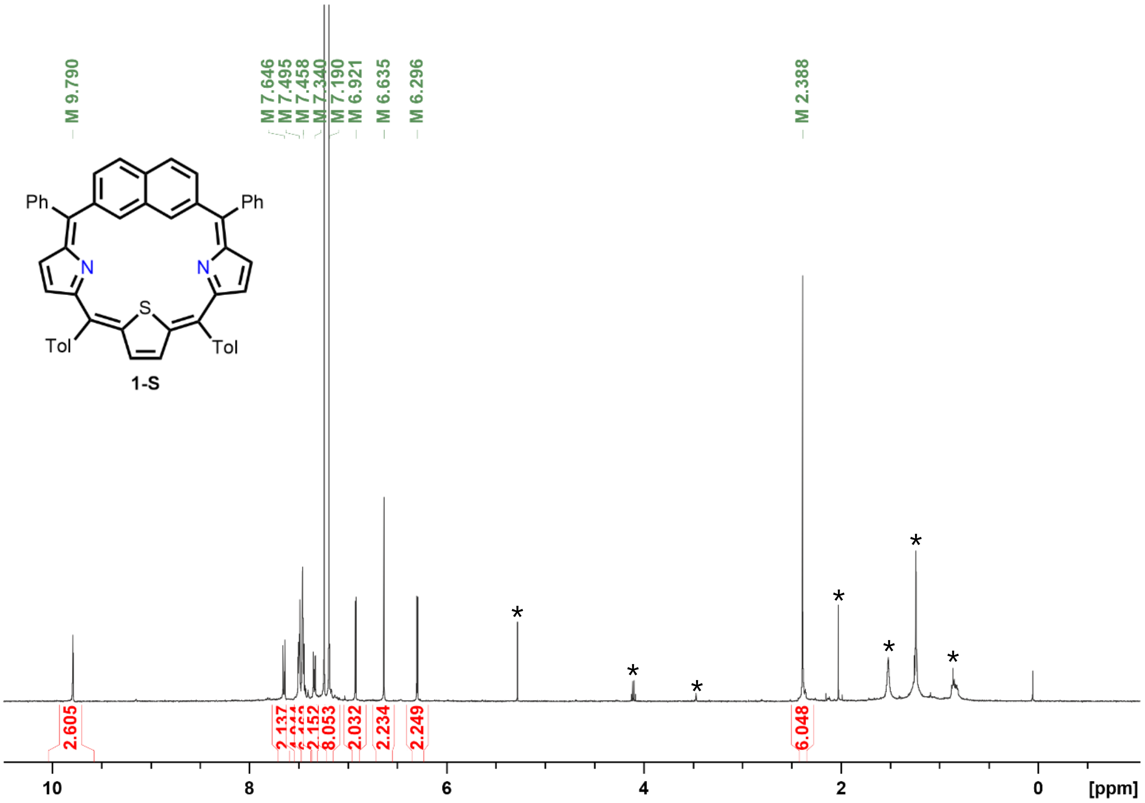

Figure S 3. The ^{1}H NMR spectrum of **1-S** (500 MHz, chloroform-*d*, 300 K). Impurities were marked with asterisks.

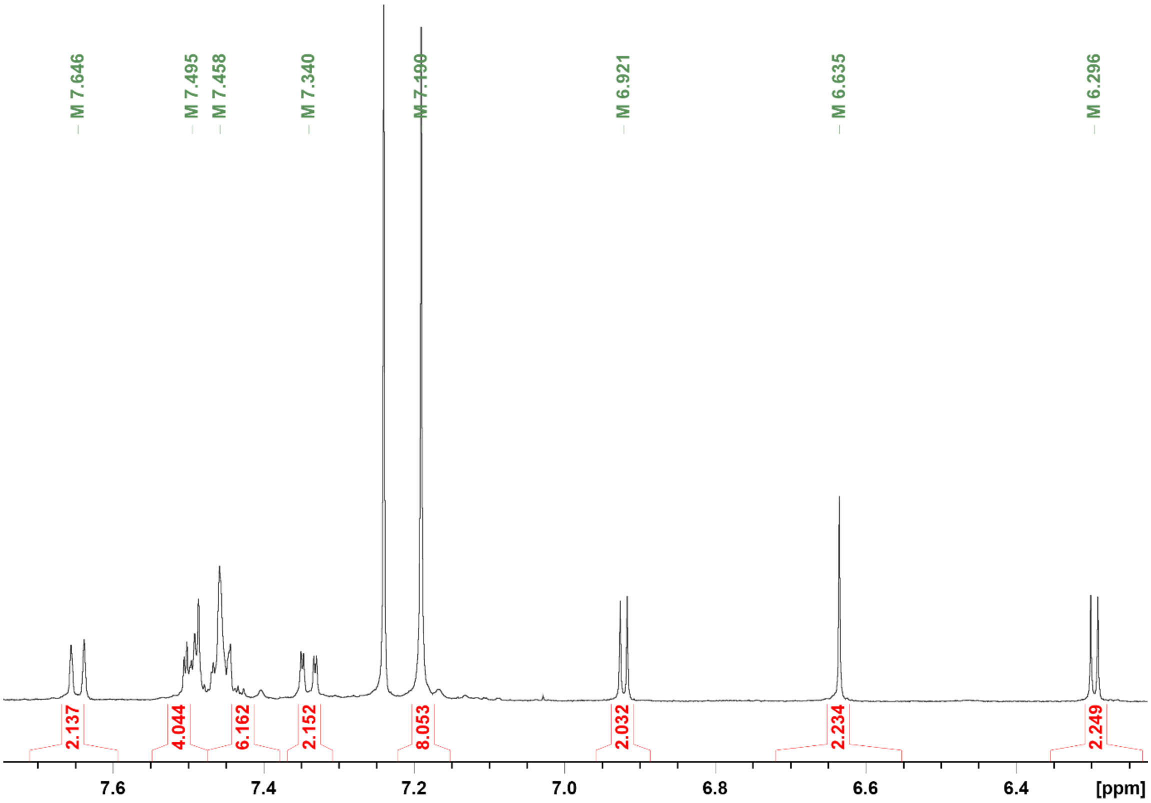

Figure S 4. Part of the ^{1}H NMR spectrum of **1-S** (500 MHz, chloroform-*d*, 300 K).

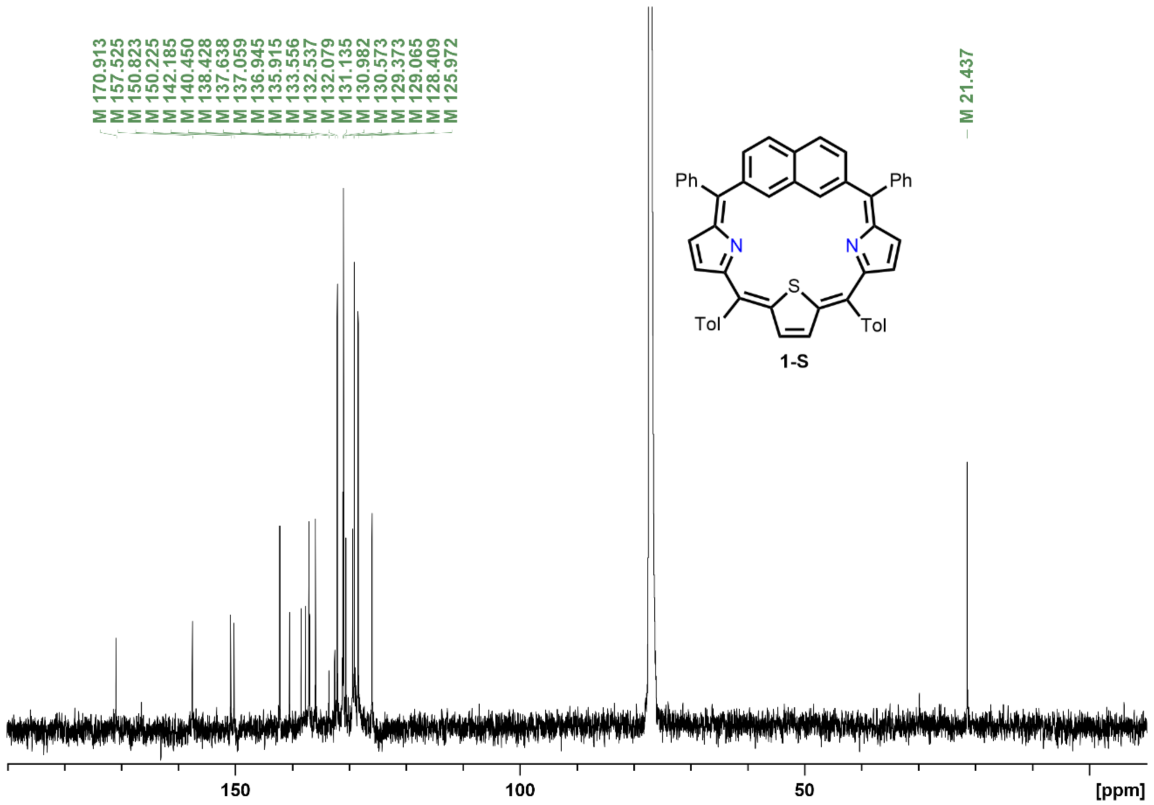

Figure S 5. The ^{13}C NMR spectrum of **1-S** (151 MHz, chloroform-*d*, 300 K).

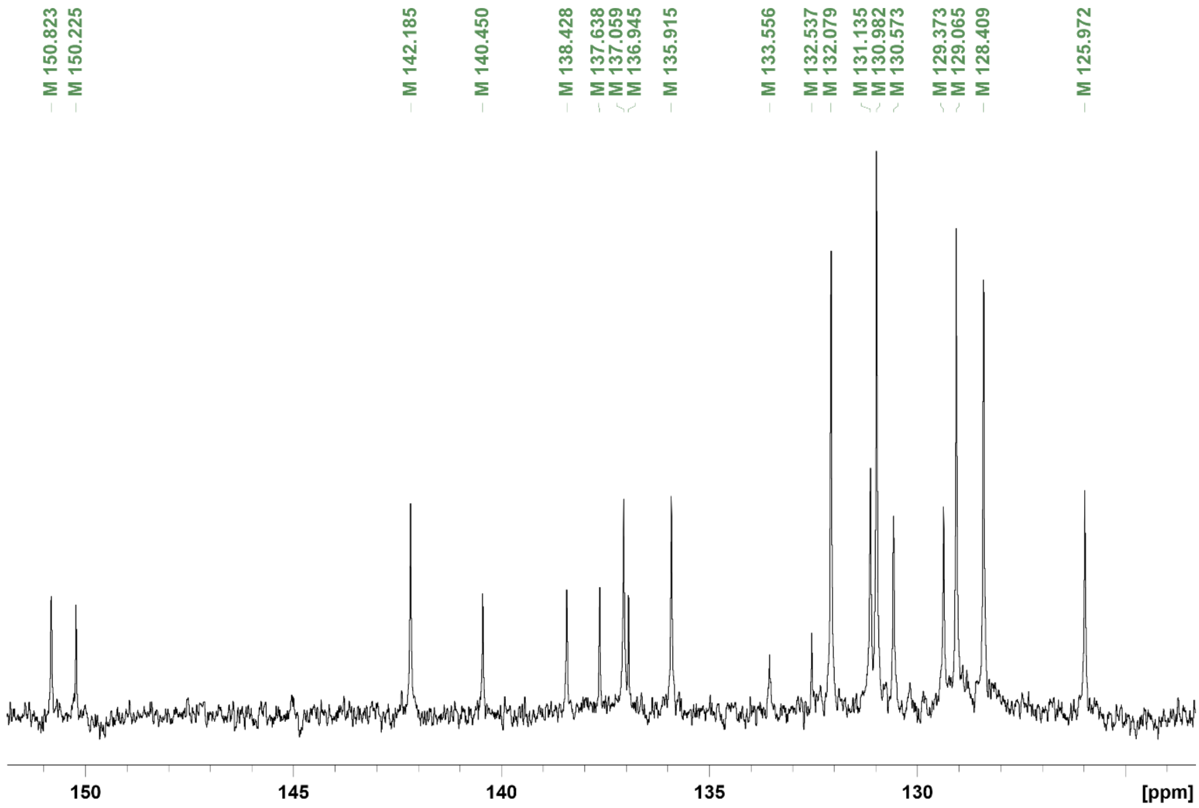

Figure S 6. Part of the ^{13}C NMR spectrum of **1-S** (151 MHz, chloroform-*d*, 300 K).

NMR spectra of macrocycle 2-Se

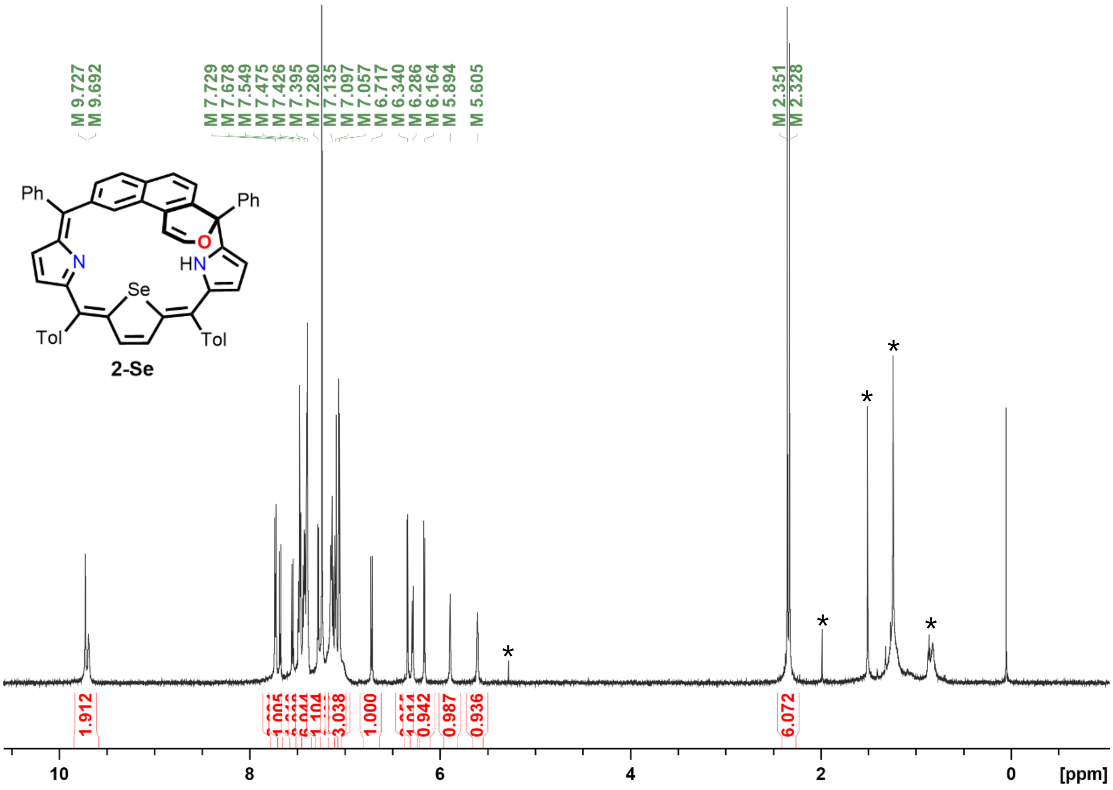

Figure S 7. The ^{1}H NMR spectrum of **2-Se** (500 MHz, chloroform-*d*, 300 K). Impurities were marked with asterisks.

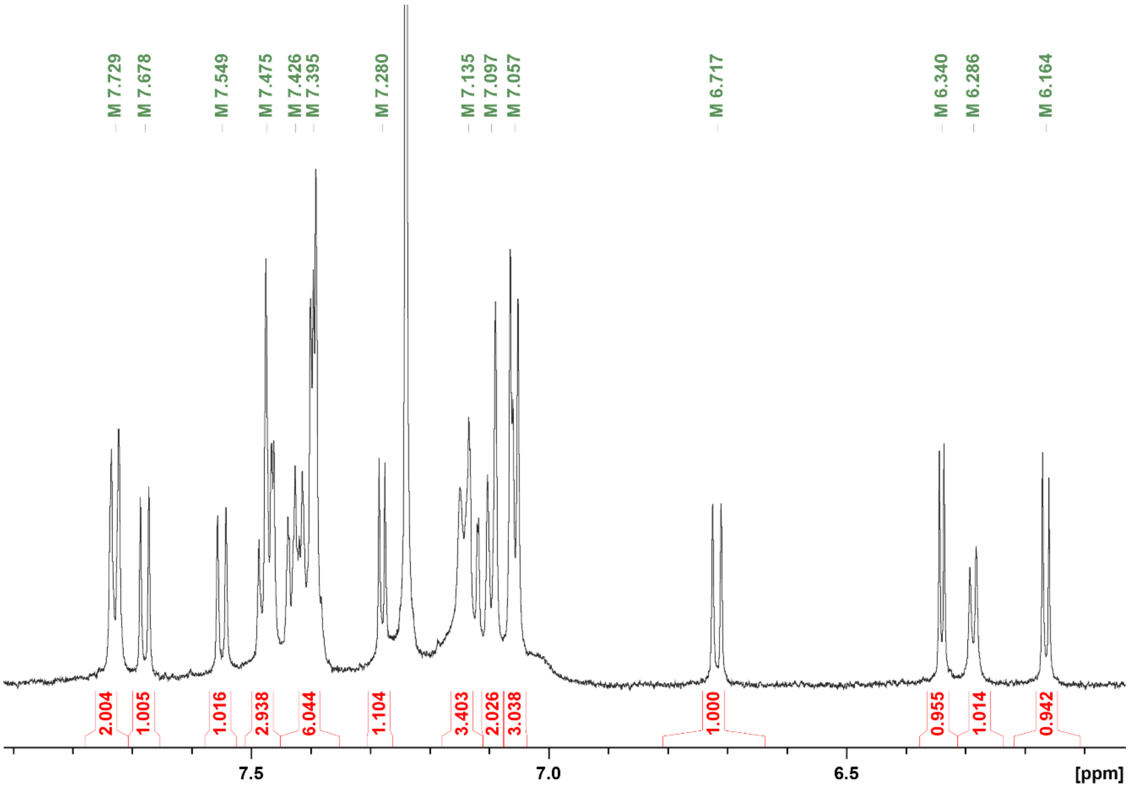

Figure S 8. Part of the ^{1}H NMR spectrum of **2-Se** (500 MHz, chloroform-*d*, 300 K).

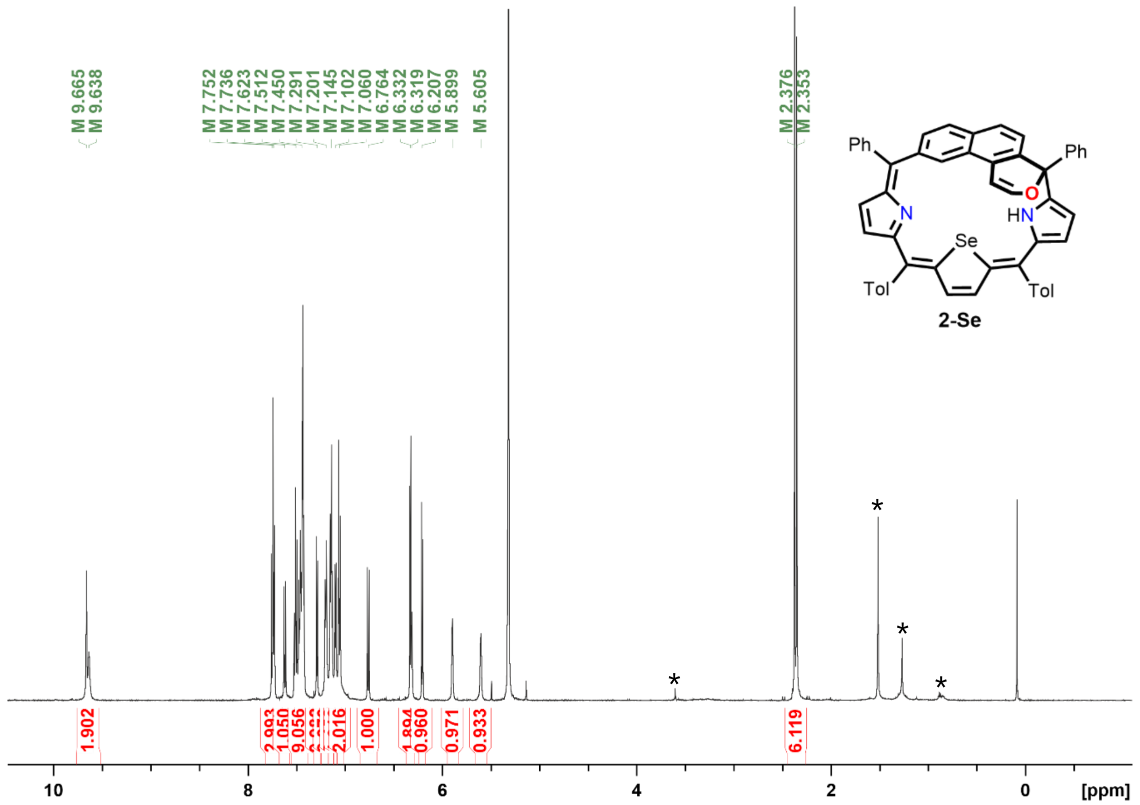

Figure S 9. The ^{1}H NMR spectrum of **2-Se** (500 MHz, dichloromethane-d_2, 300 K). Impurities were marked with asterisks.

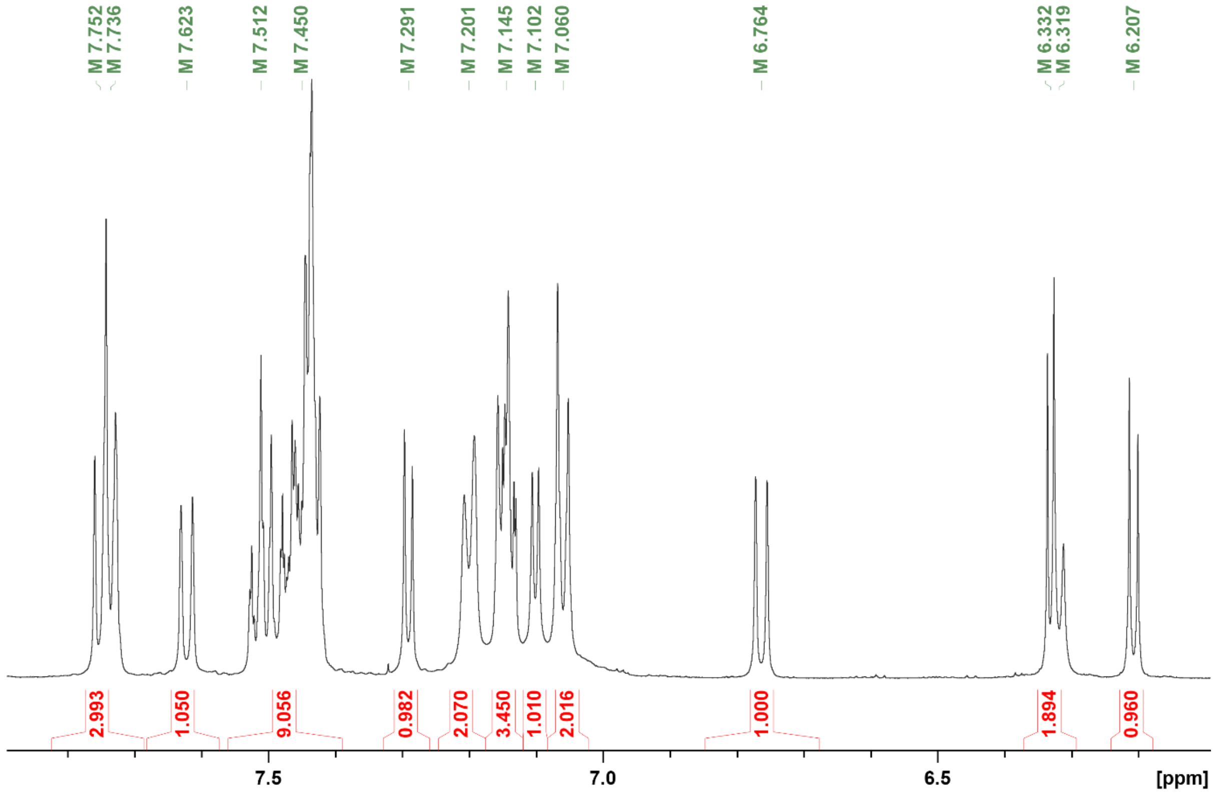

Figure S 10. Part of the ^{1}H NMR spectrum of **2-Se** (500 MHz, dichloromethane-d_2, 300 K).

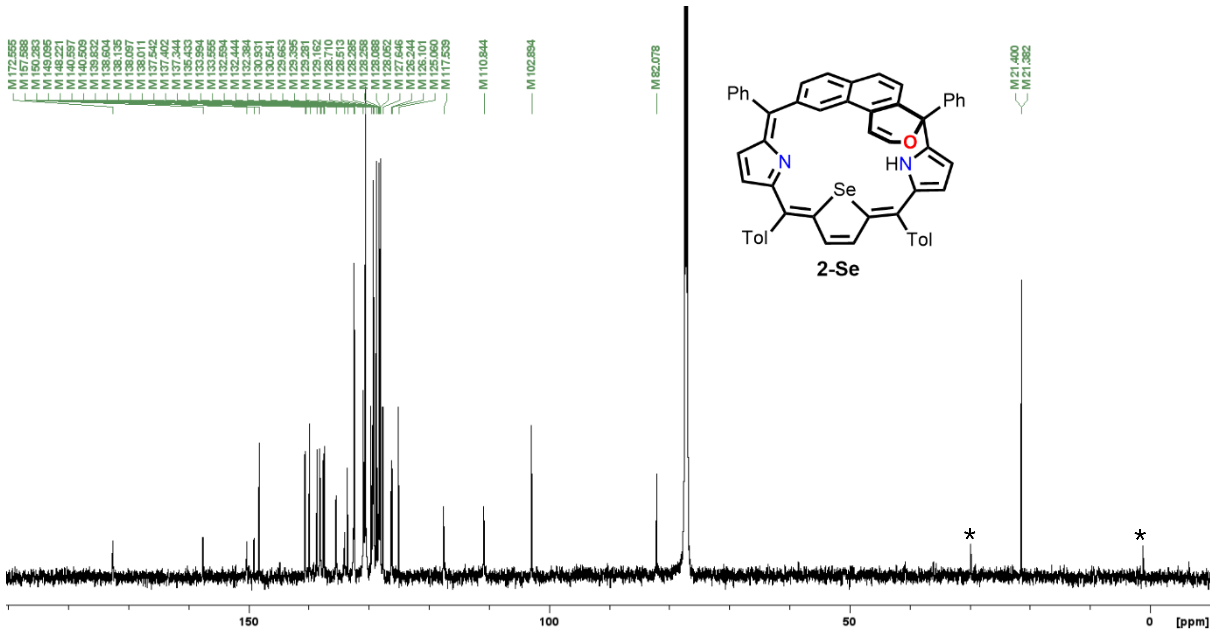

Figure S 11. The ^{13}C NMR spectrum of **2-Se** (151 MHz, chloroform-d, 300 K). Impurities were marked with asterisks.

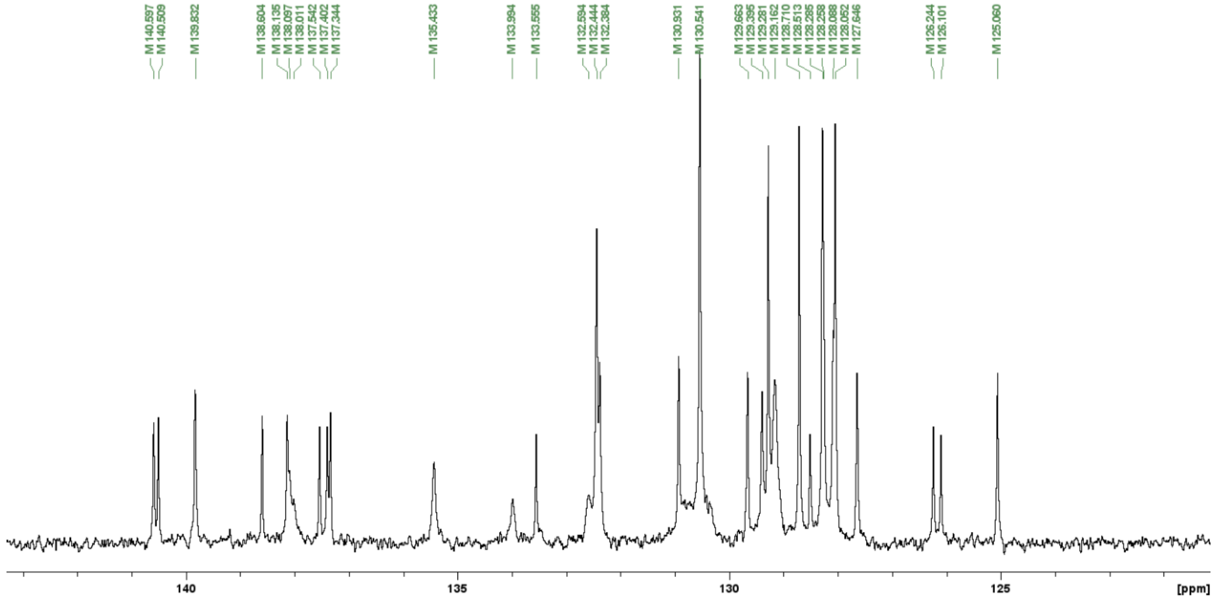

Figure S 12. Part of the ^{13}C NMR spectrum of **2-Se** (151 MHz, chloroform-*d*, 300 K).

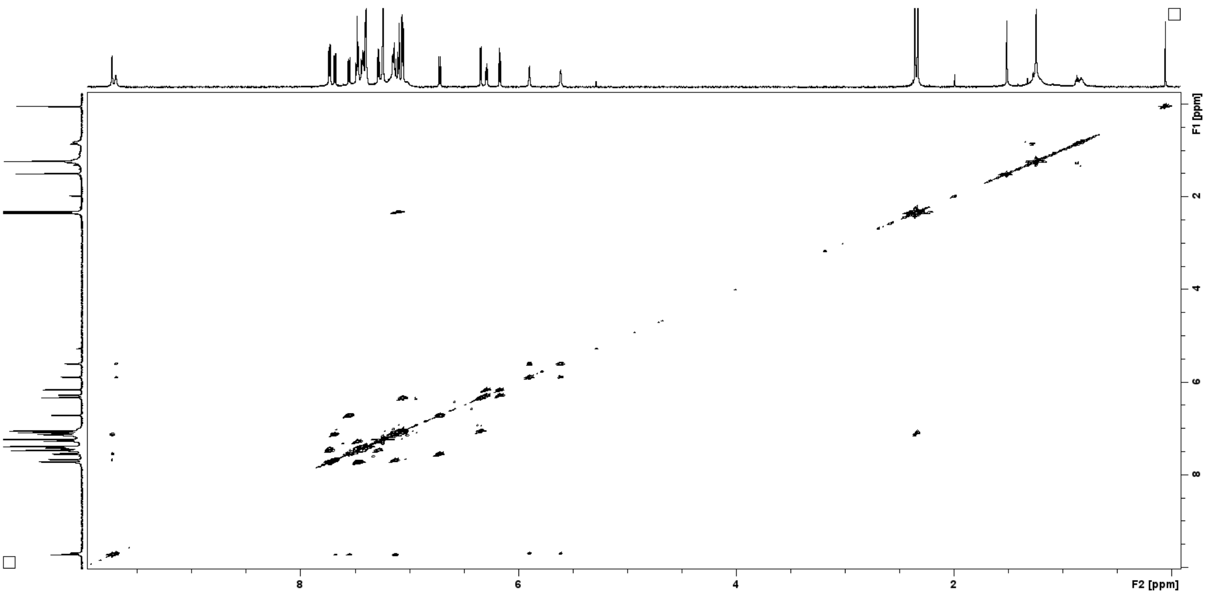

Figure S 13. The ^{1}H-^{1}H COSY NMR spectrum of **2-Se** (600 MHz, chloroform-*d*, 300 K).

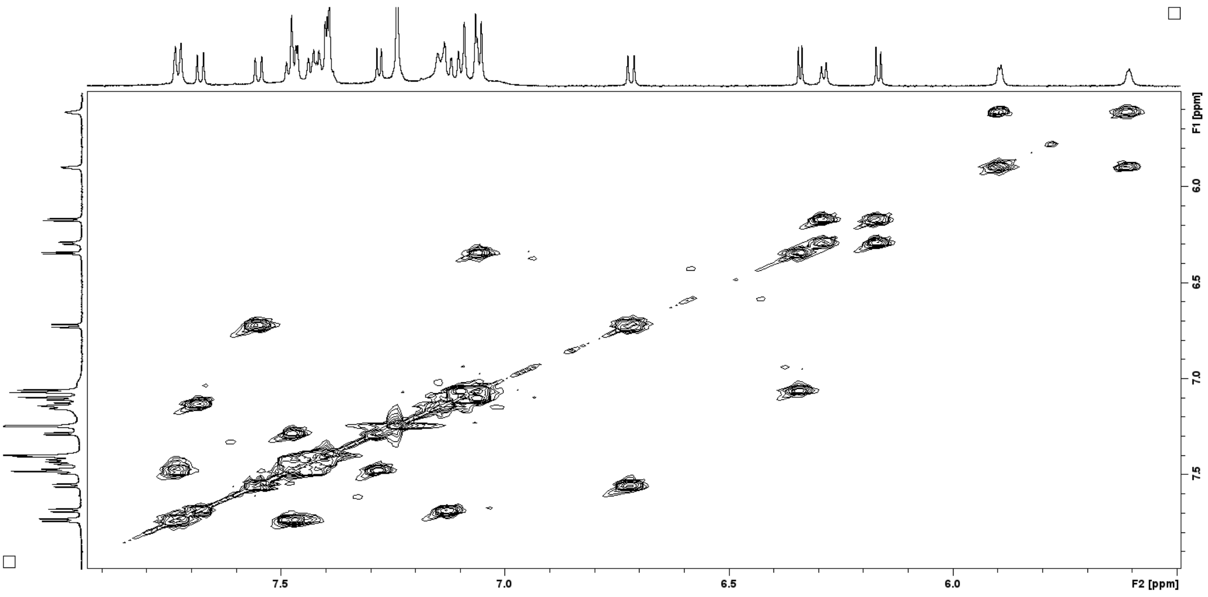

Figure S 14. Part of the ^{1}H-^{1}H COSY NMR spectrum of **2-Se** (600 MHz, chloroform-*d*, 300 K).

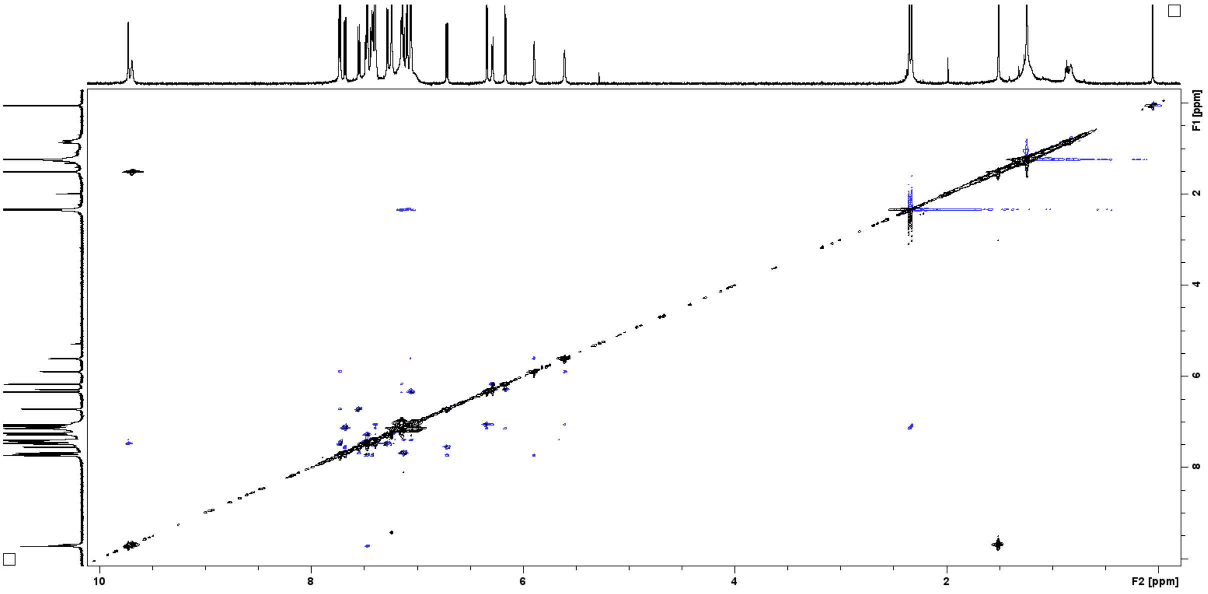

Figure S 15. The ^{1}H-^{1}H NOESY NMR spectrum of **2-Se** (600 MHz, chloroform-*d*, 300 K).

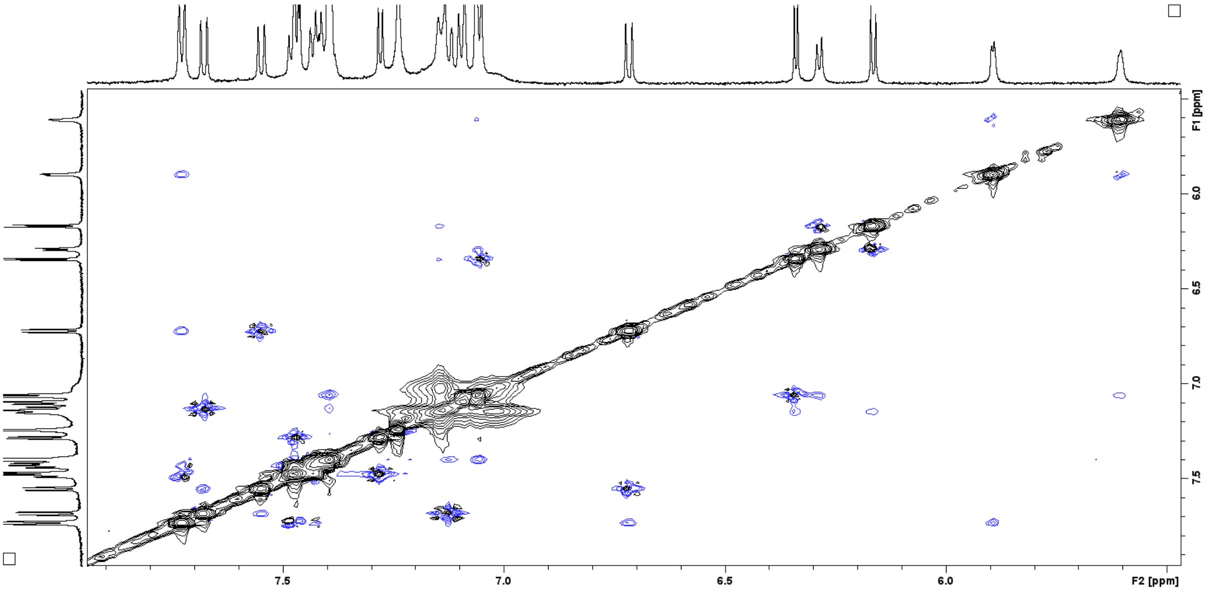

Figure S 16. Part of the ^{1}H-^{1}H NOESY NMR spectrum of **2-Se** (600 MHz, chloroform-*d*, 300 K).

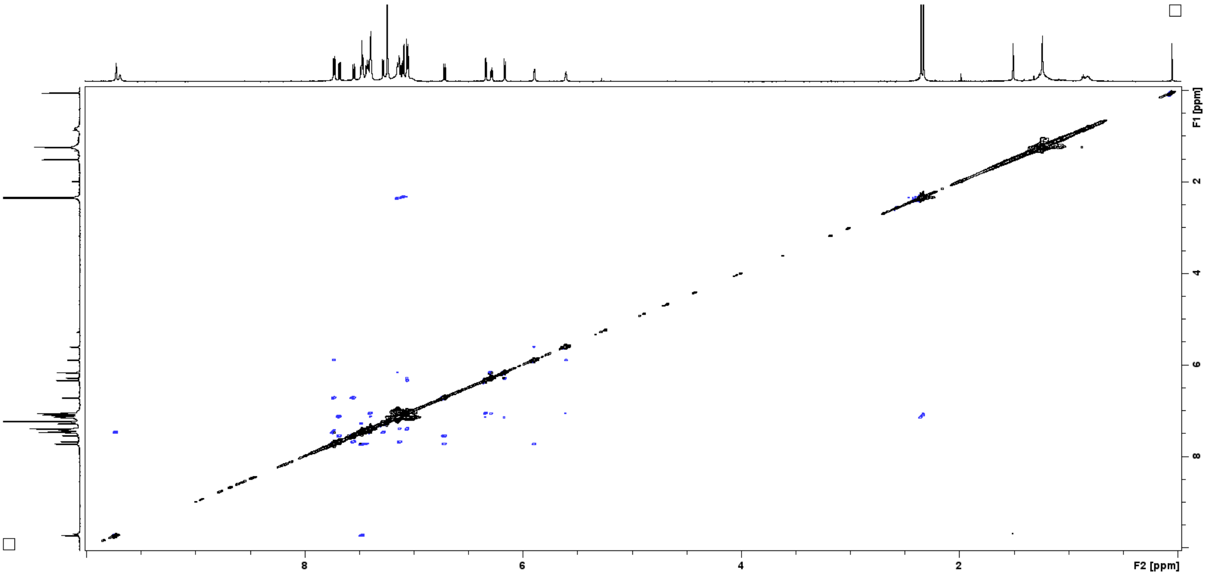

Figure S 17. The ^{1}H-^{1}H ROESY NMR spectrum of **2-Se** (600 MHz, chloroform-*d*, 300 K).

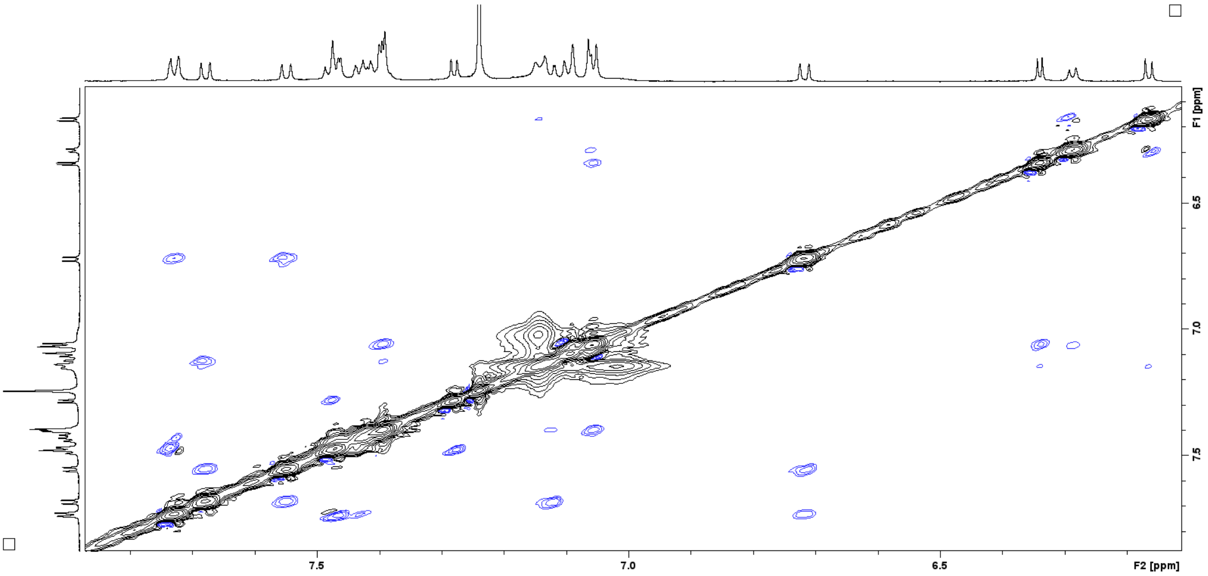

Figure S 18. Part of the ^{1}H-^{1}H ROESY NMR spectrum of **2-Se** (600 MHz, chloroform-*d*, 300 K).

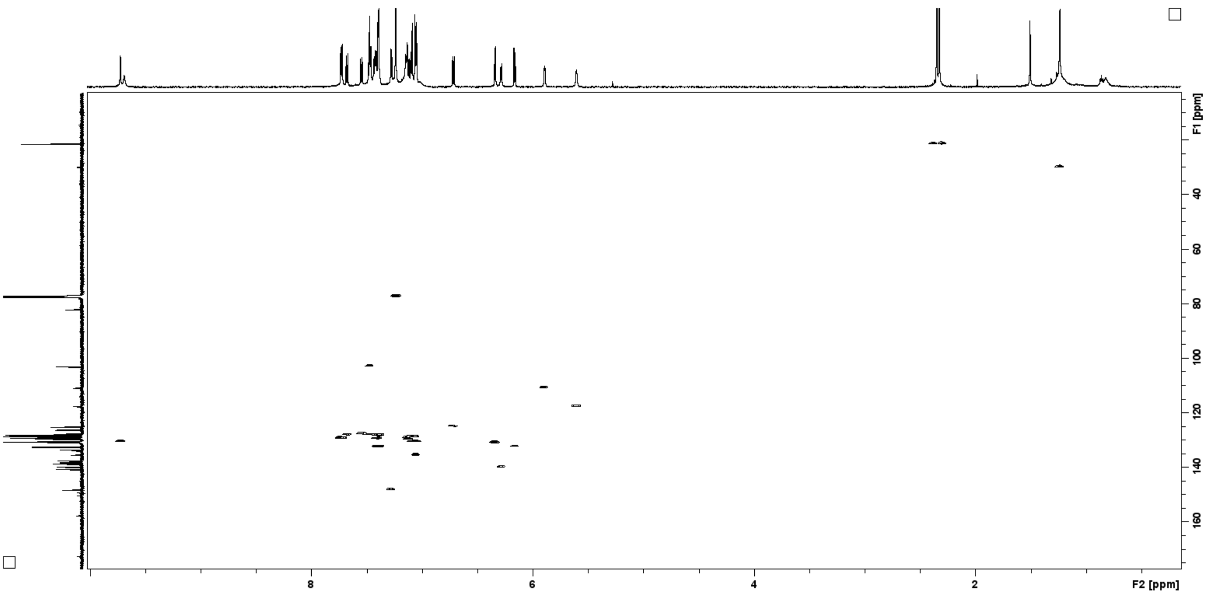

Figure S 19. The ^{1}H-^{13}C HMQC NMR spectrum of **2-Se** (600 MHz, chloroform-*d*, 300 K).

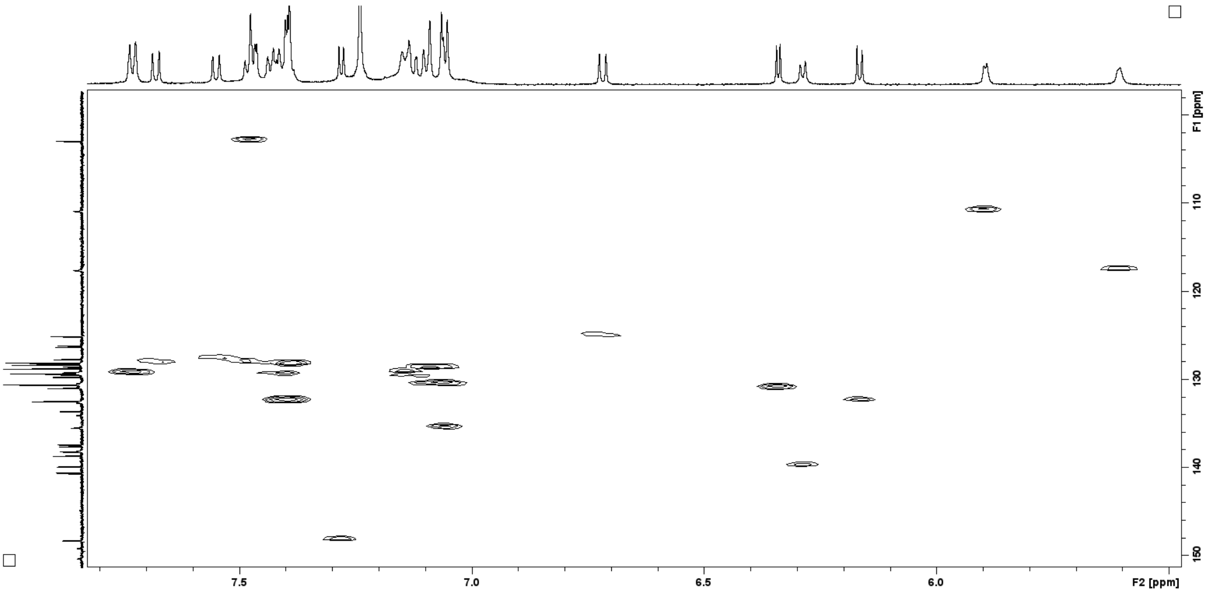

Figure S 20. Part of the ^{1}H-^{13}C HMQC NMR spectrum of **2-Se** (600 MHz, chloroform-*d*, 300 K).

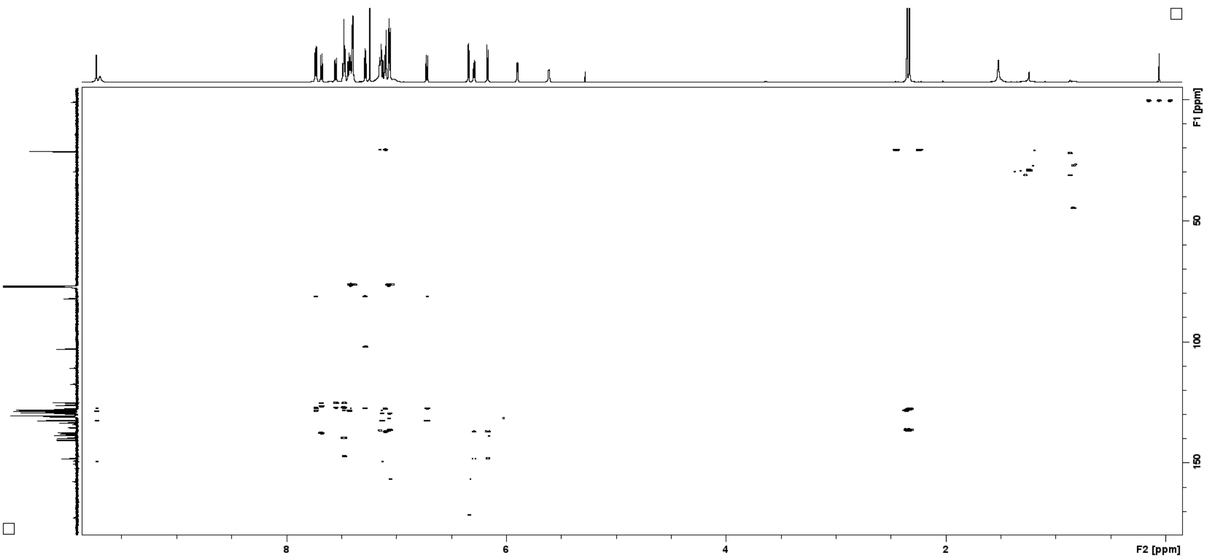

Figure S 21. The ^{1}H-^{13}C HMBC NMR spectrum of **2-Se** (600 MHz, chloroform-*d*, 300 K).

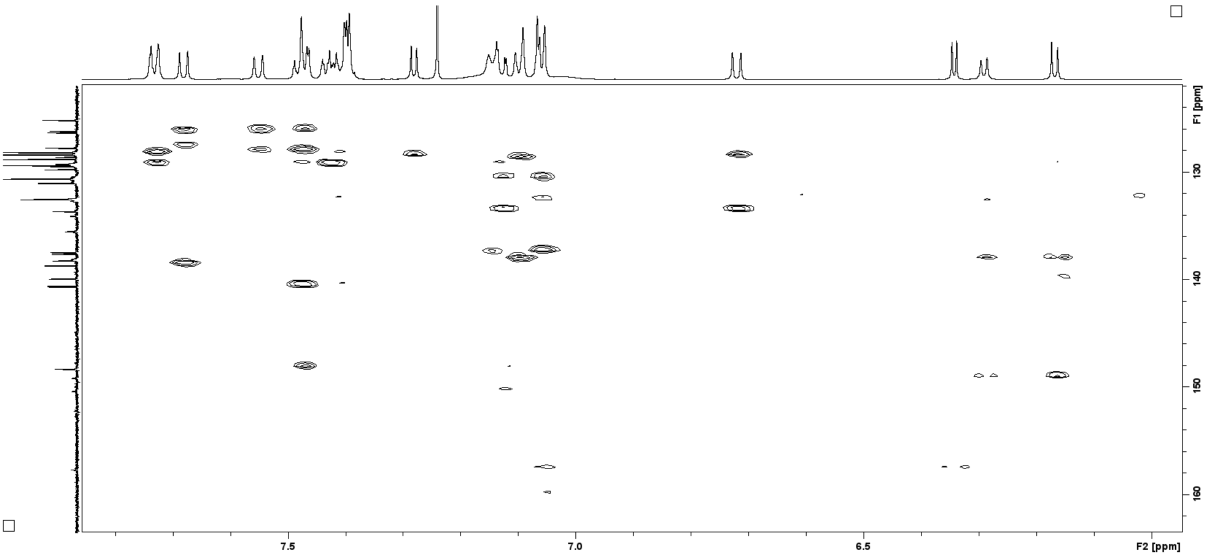

Figure S 22. Part of the ^{1}H-^{13}C HMBC NMR spectrum of **2-Se** (600 MHz, chloroform-*d*, 300 K).

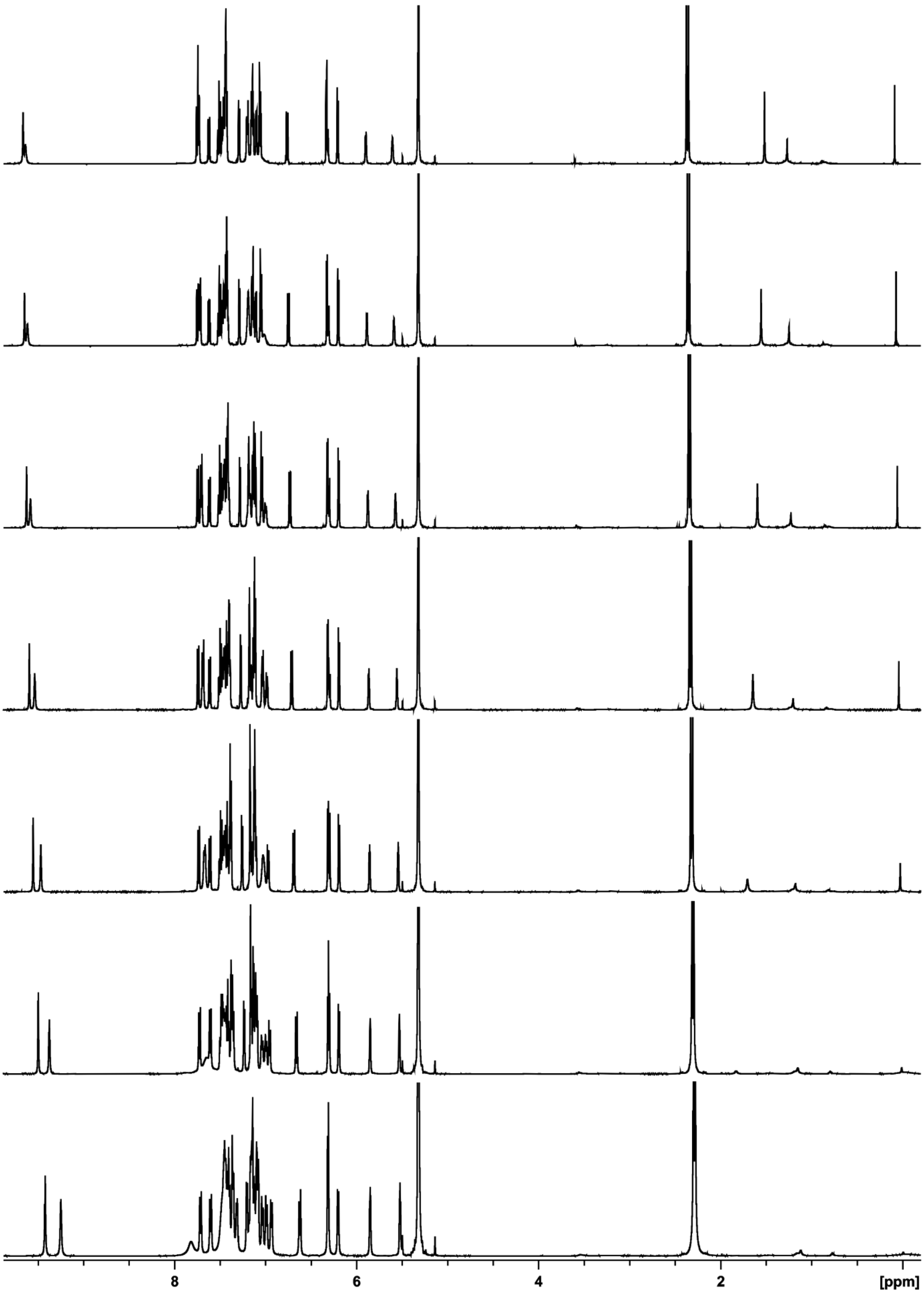

Figure S 23. The ^{1}H NMR spectra of **2-Se** recorded every 20 K in the 300 K (top) – 180 K (bottom) temperature range (600 MHz, dichloromethane-d_2).

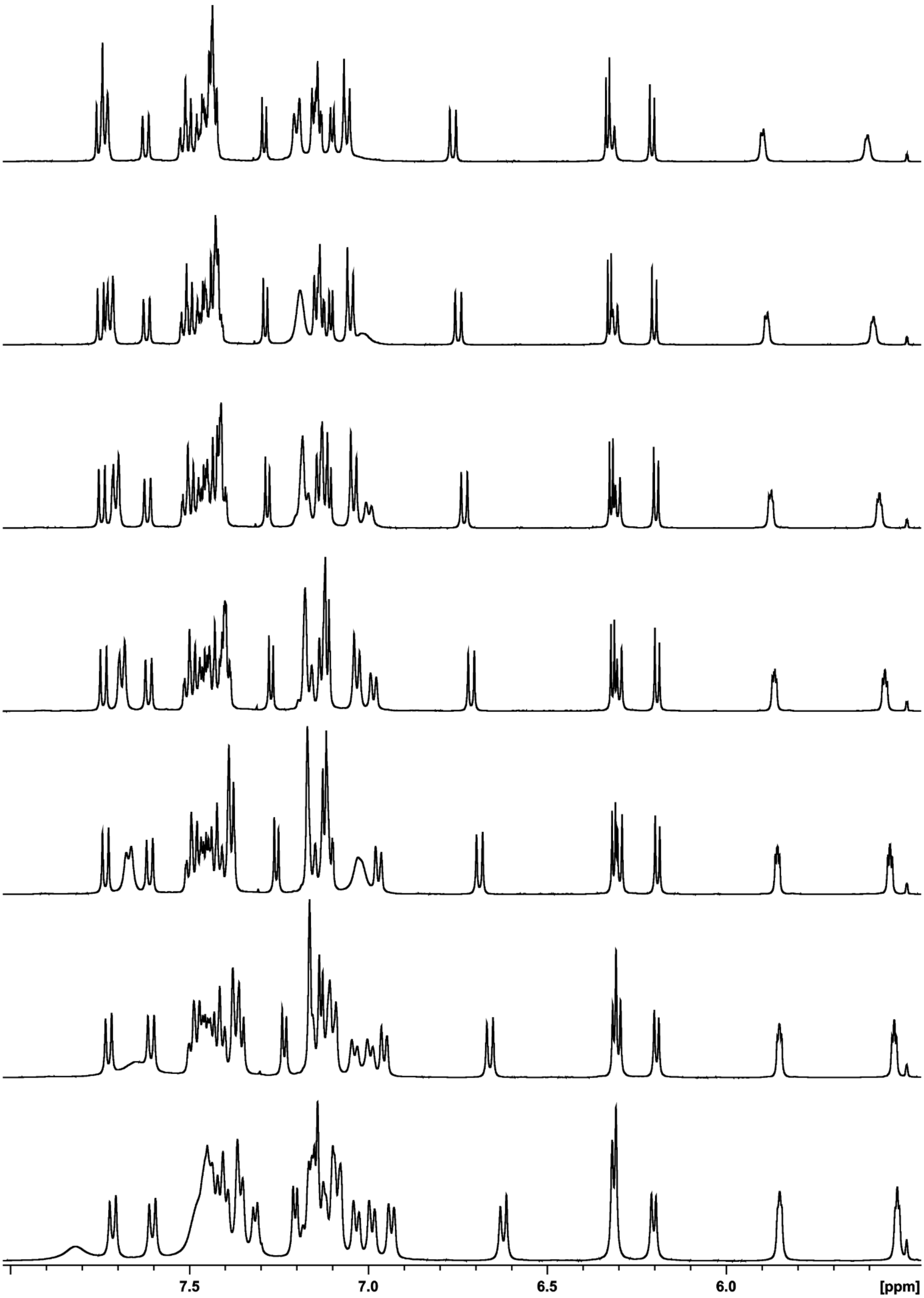

Figure S 24. Part of the ^1H NMR spectra of **2-Se** recorded every 20 K in the 300 K (top) – 180 K (bottom) temperature range (600 MHz, dichloromethane-d_2).

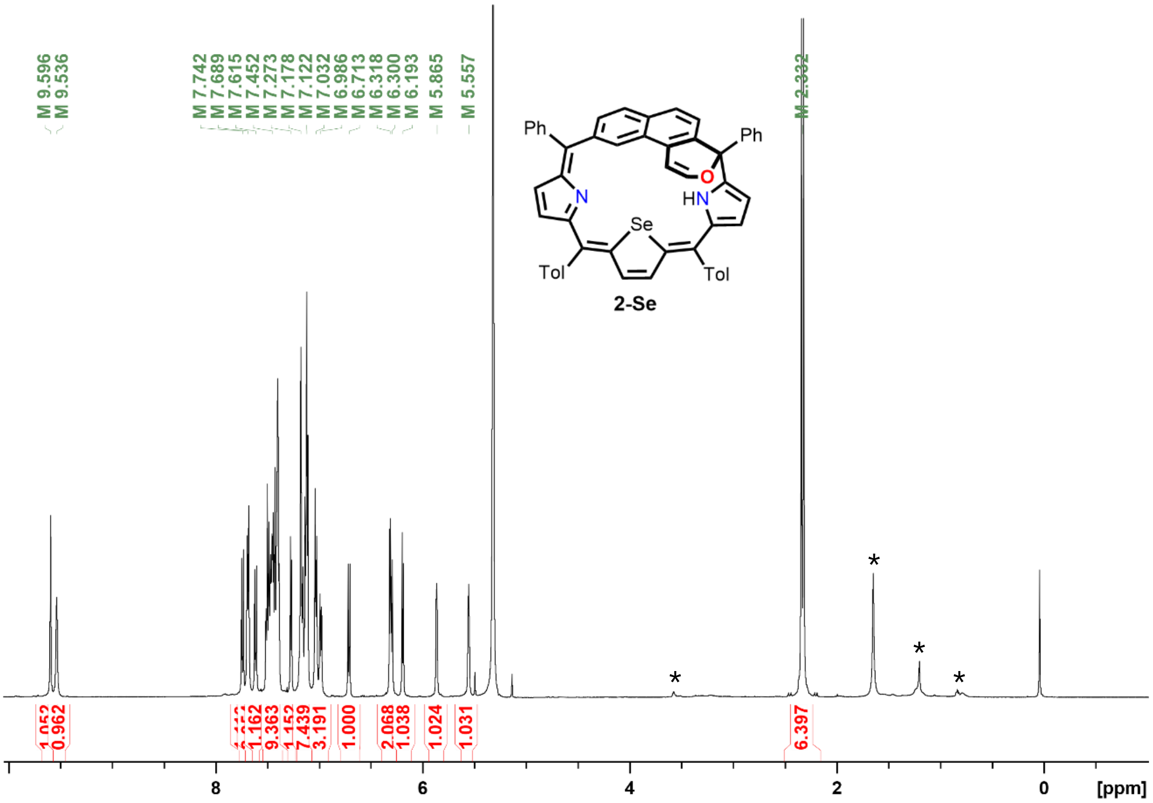

Figure S 25. The ^{1}H NMR spectrum of **2-Se** (600 MHz, dichloromethane-d_2, 240 K). Impurities were marked with asterisks.

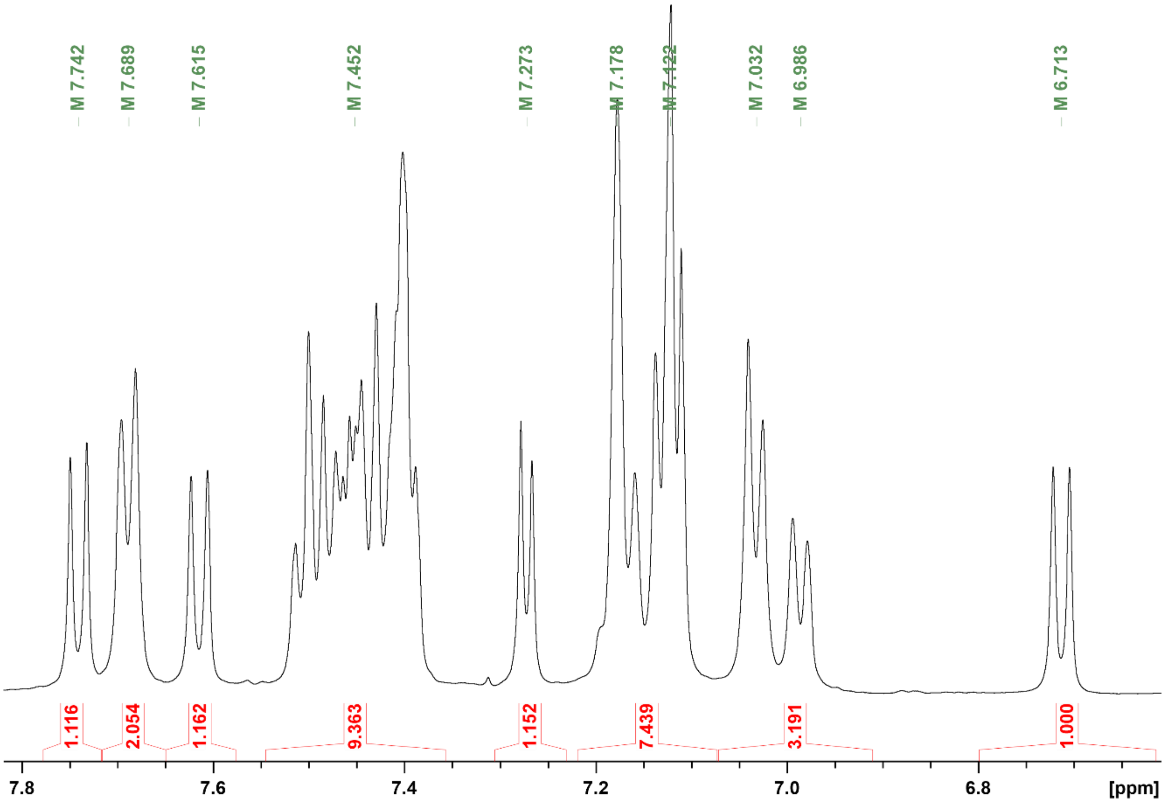

Figure S 26. Part of the ^{1}H NMR spectrum of **2-Se** (600 MHz, dichloromethane-d_2, 240 K).

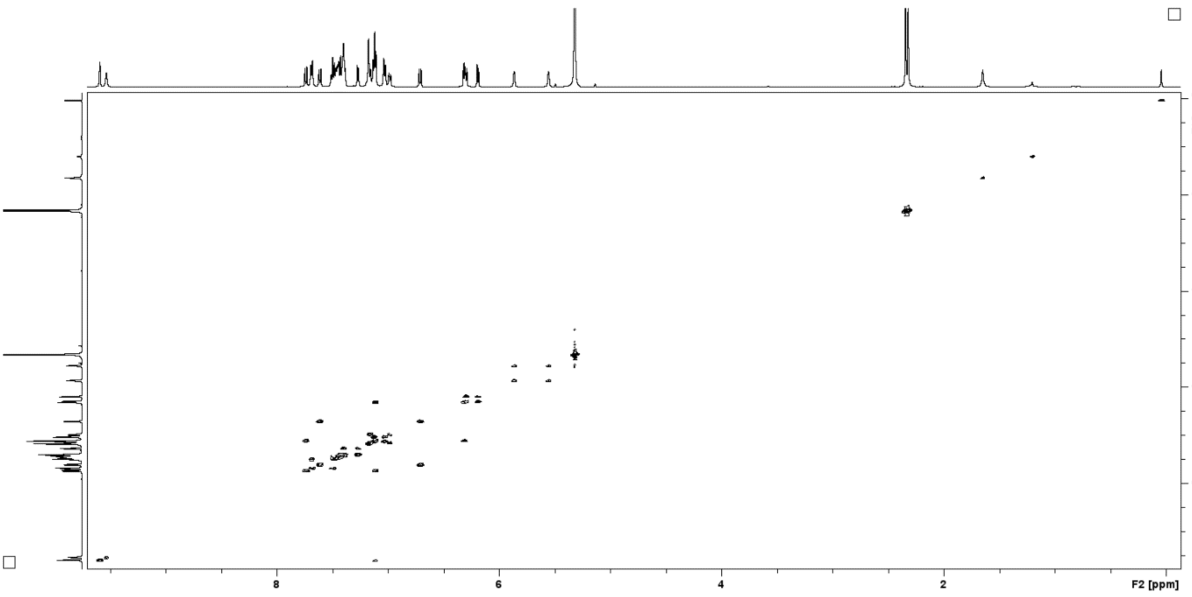

Figure S 27. The ^{1}H-^{1}H COSY NMR spectrum of **2-Se** (600 MHz, dichloromethane-*d*$_2$, 240 K).

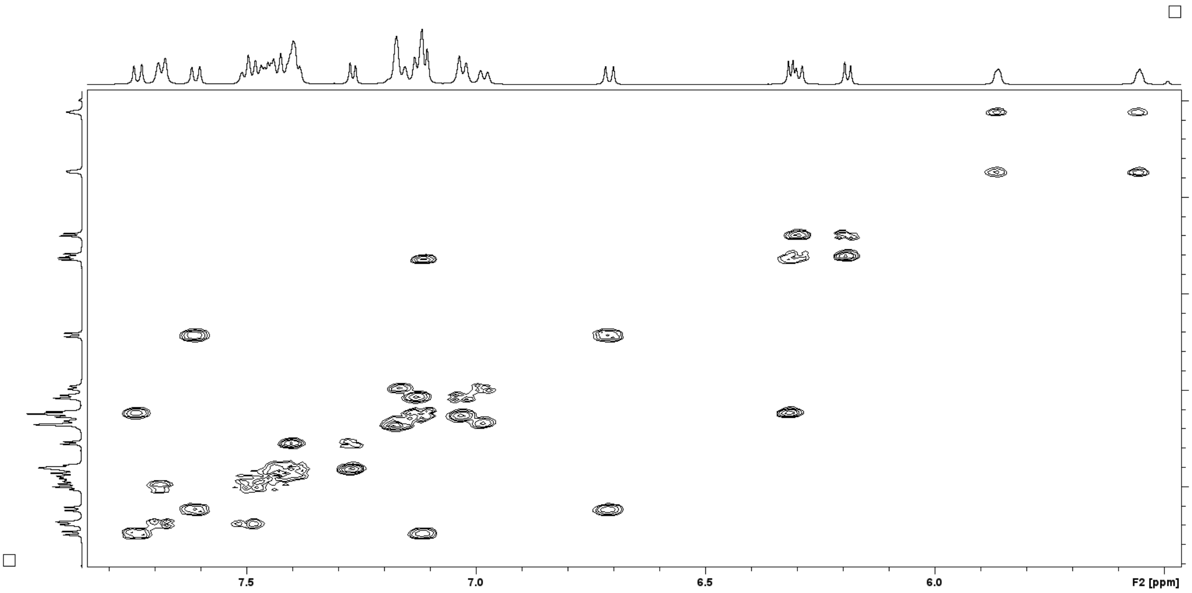

Figure S 28. Part of the ^{1}H-^{1}H COSY NMR spectrum of **2-Se** (600 MHz, dichloromethane-*d*$_2$, 240 K).

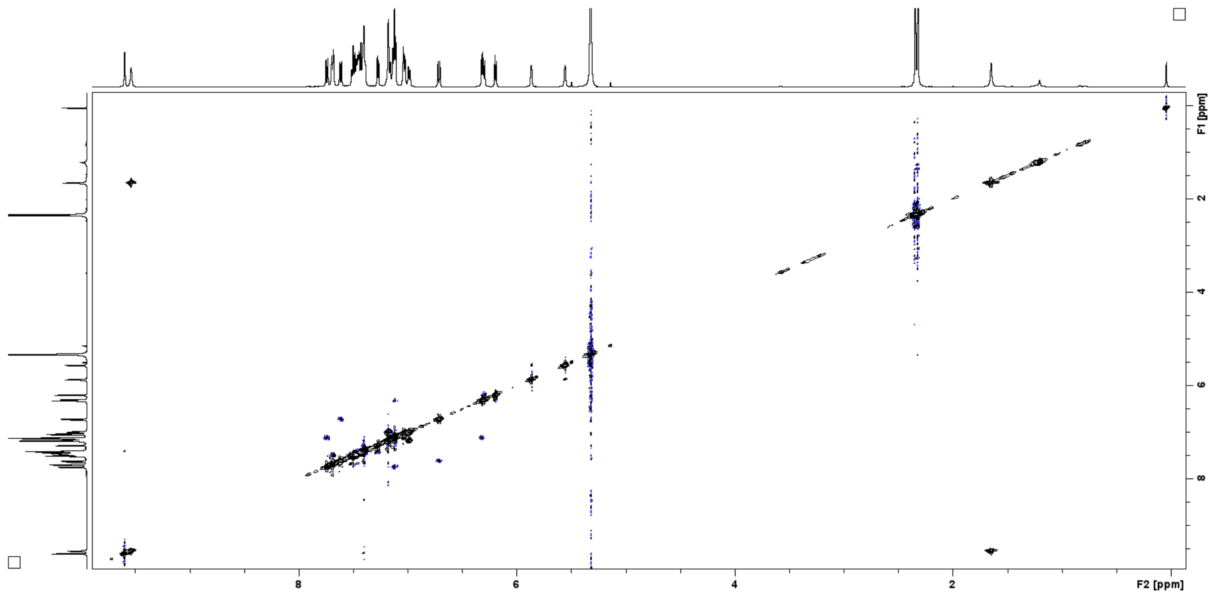

Figure S 29. The ^{1}H-^{1}H NOESY spectrum of **2-Se** (600 MHz, dichloromethane-d_2, 240 K).

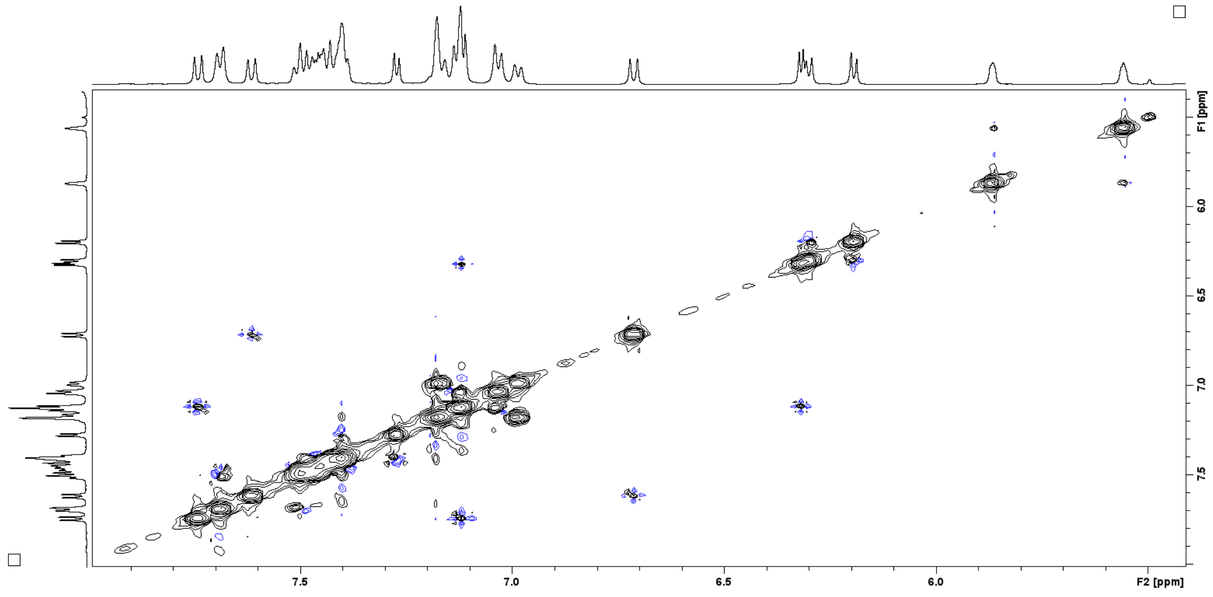

Figure S 30. Part of the ^{1}H-^{1}H NOESY NMR spectrum of **2-Se** (600 MHz, dichloromethane-d_2, 240 K).

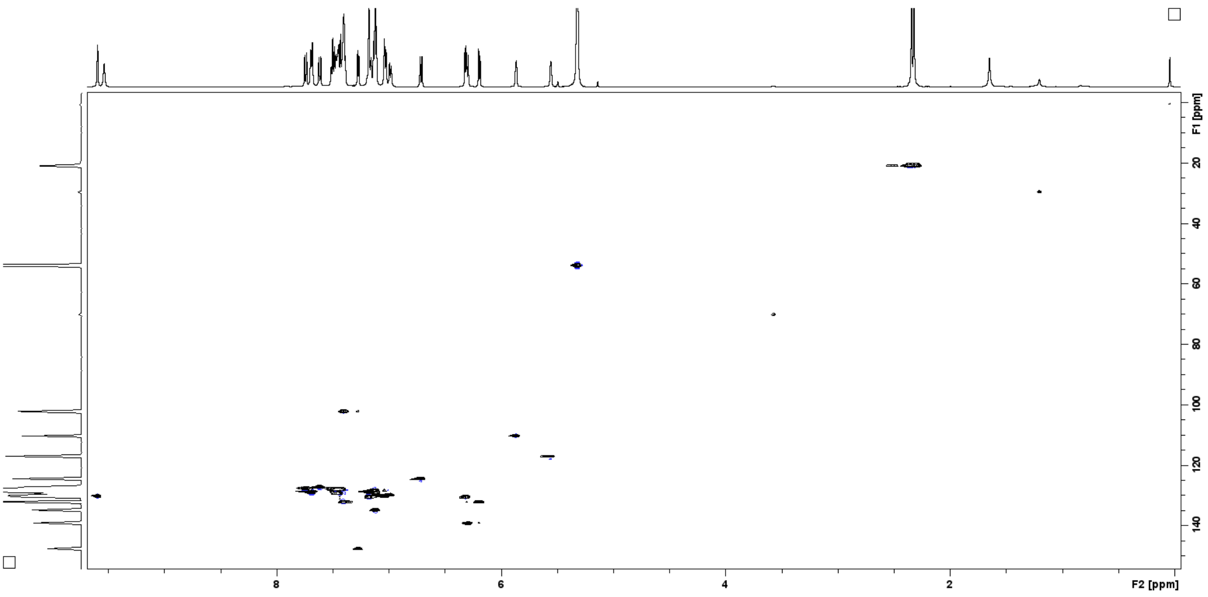

Figure S 31. The ^{1}H-^{13}C HSQC NMR spectrum of **2-Se** (600 MHz, dichloromethane-*d*2, 240 K).

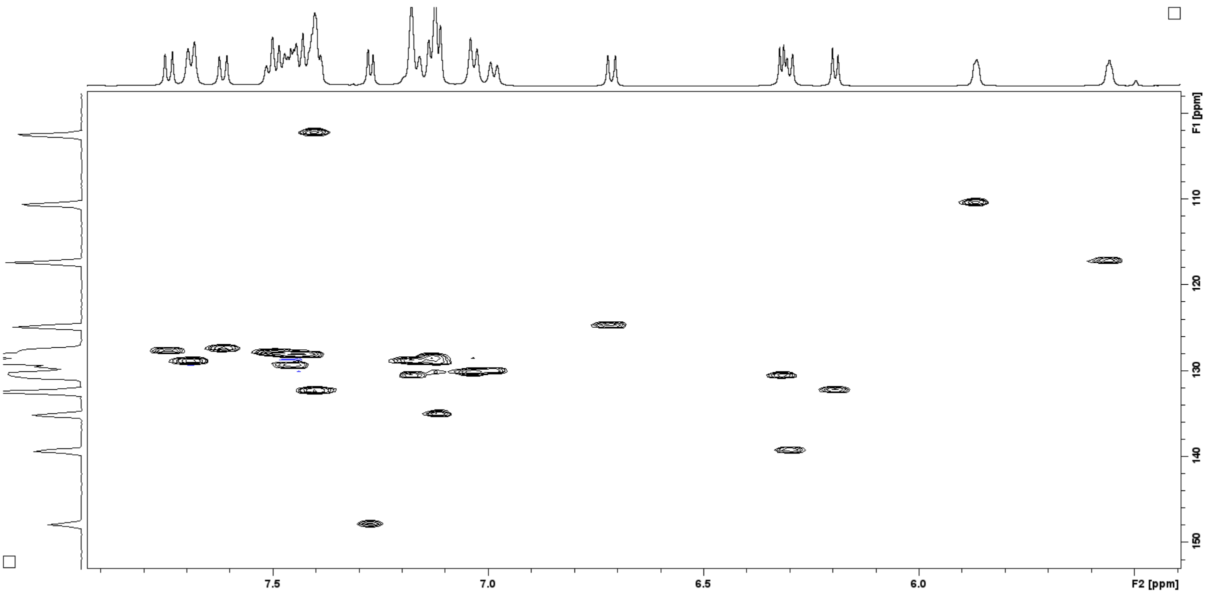

Figure S 32. Part of the ^{1}H-^{13}C HSQC NMR spectrum of **2-Se** (600 MHz, dichloromethane-*d*2, 240 K).

NMR spectra of macrocycle 3-Se

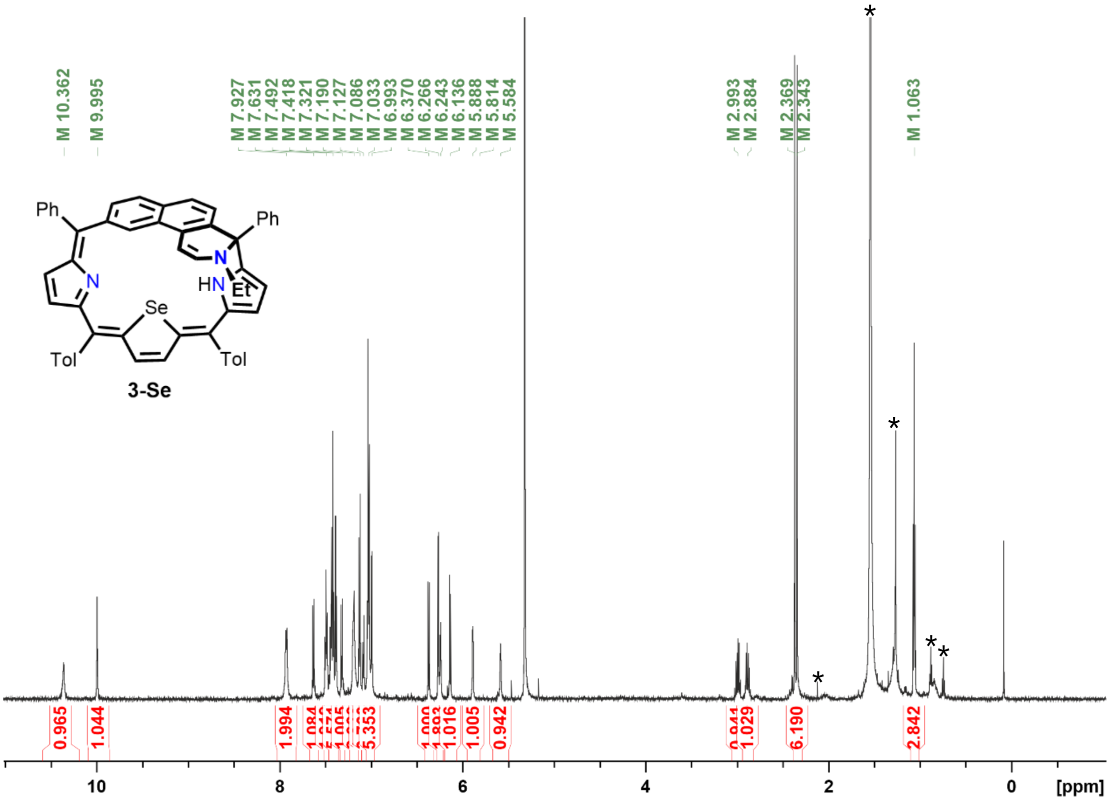

Figure S 33. The ^{1}H NMR spectrum of **3-Se** (600 MHz, dichloromethane-d_2, 300 K). Impurities were marked with asterisks.

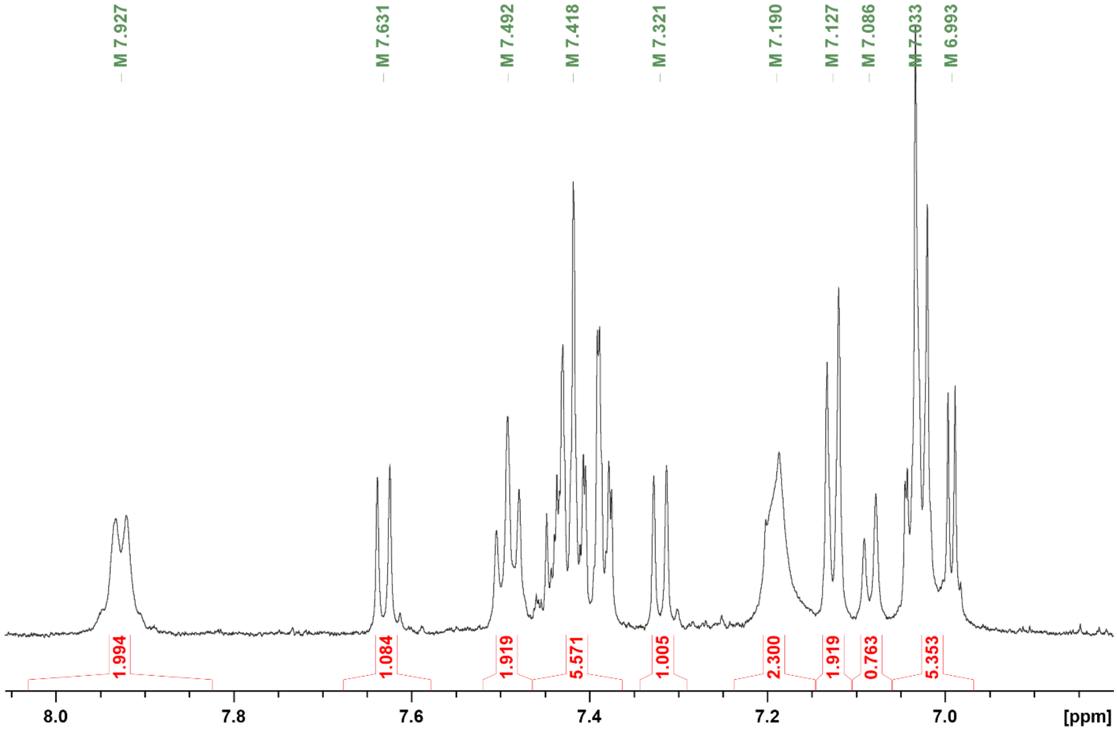

Figure S 34. Part of the ^{1}H NMR spectrum of **3-Se** (600 MHz, dichloromethane-d_2, 300 K).

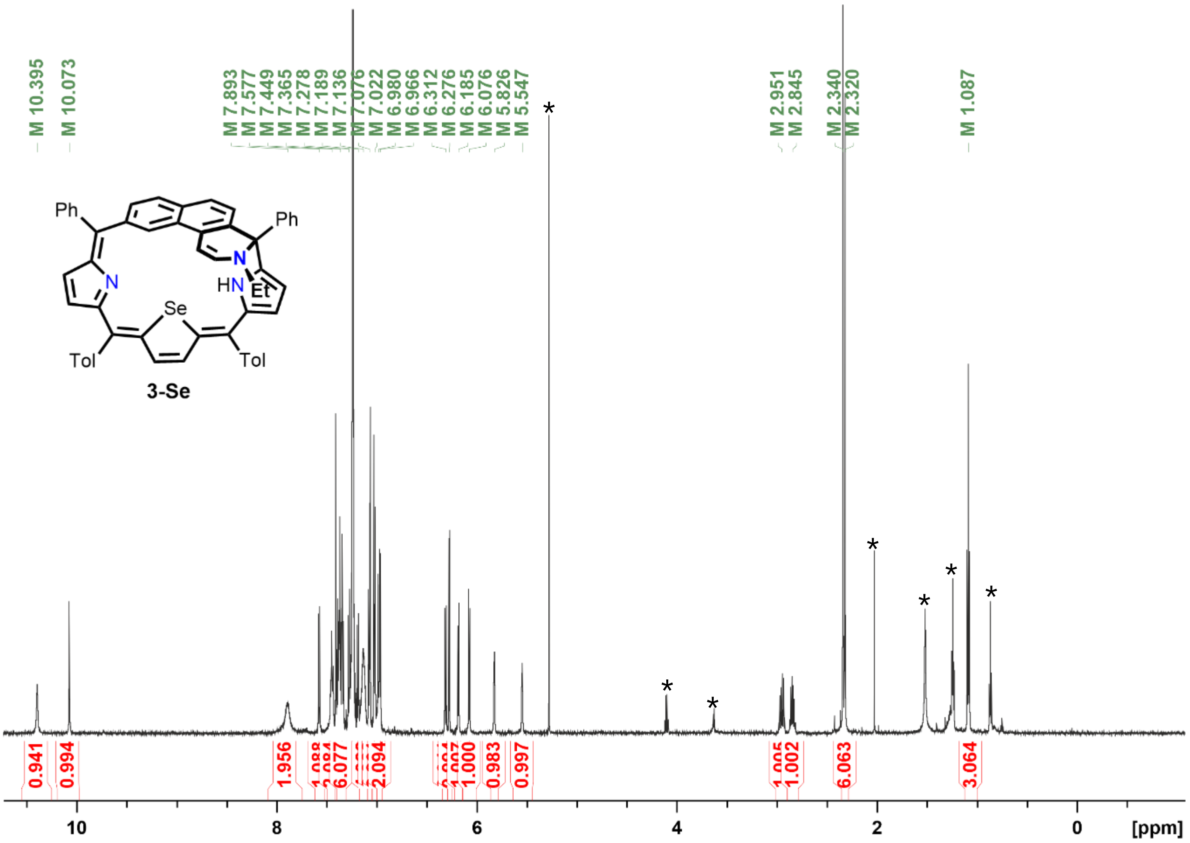

Figure S 35. The ^{1}H NMR spectrum of **3-Se** (600 MHz, chloroform-*d*, 300 K). Impurities were marked with asterisks.

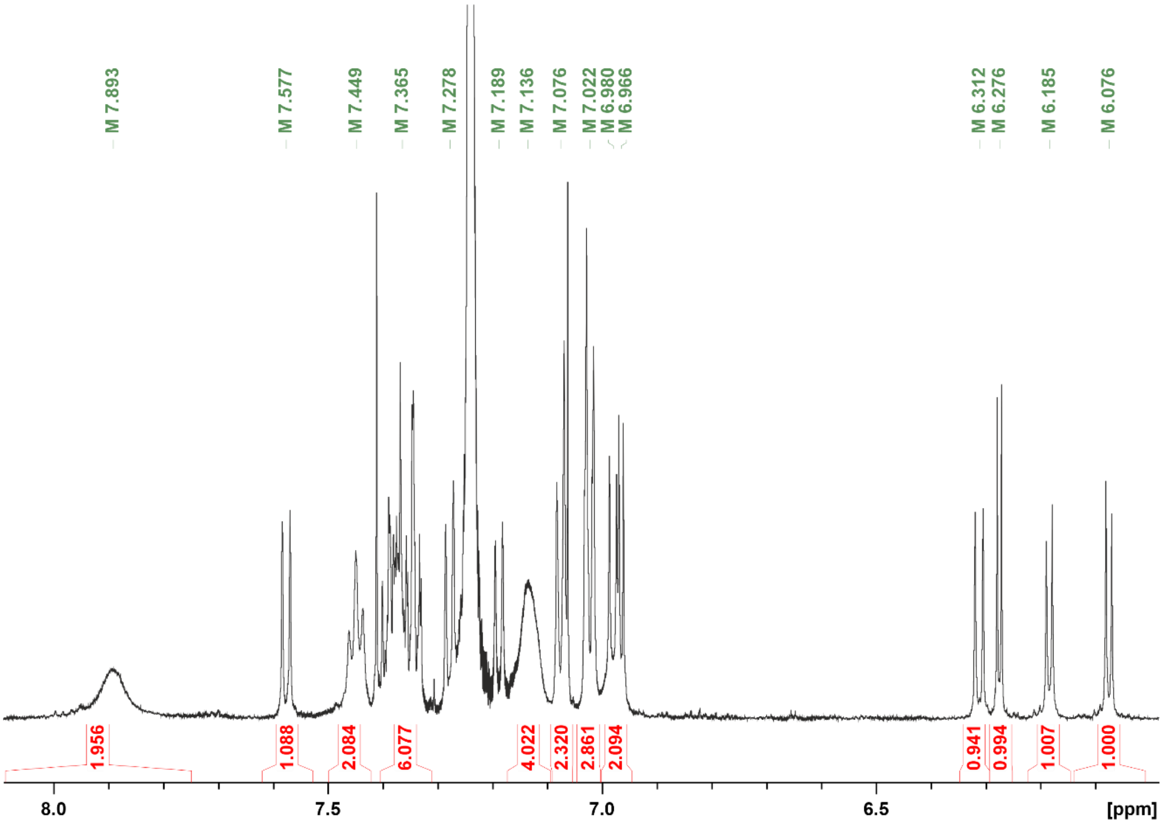

Figure S 36. Part of the ^{1}H NMR spectrum of **3-Se** (600 MHz, chloroform-*d*, 300 K).

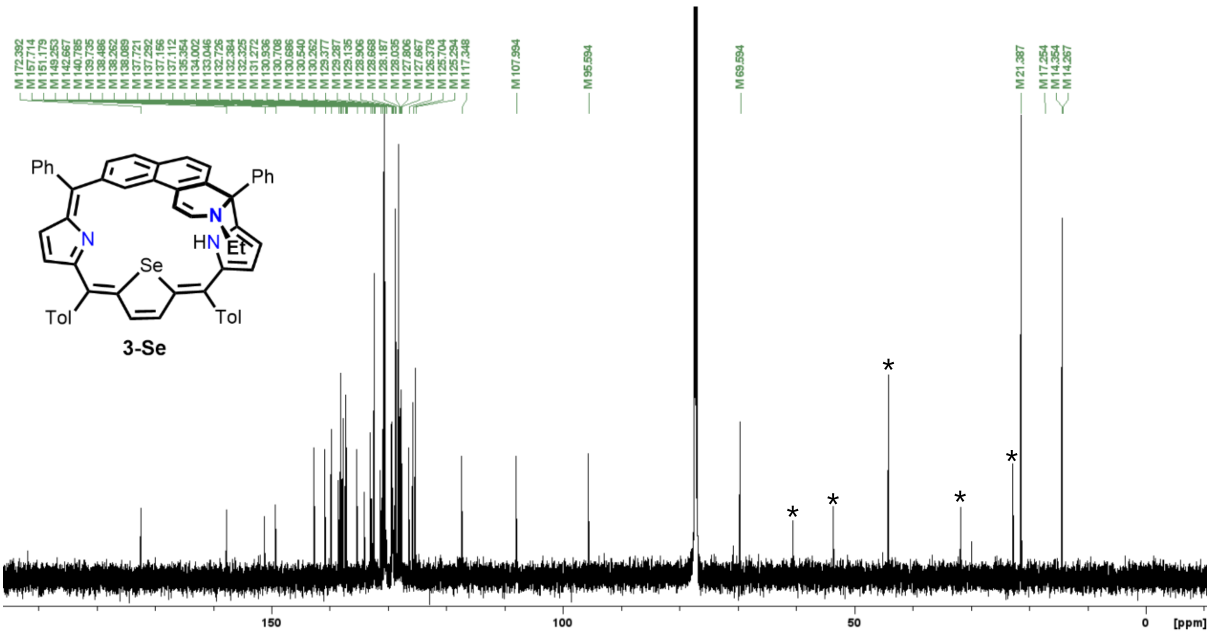

Figure S 37. The ^{13}C NMR spectrum of **3-Se** (151 MHz, chloroform-*d*, 300 K). Impurities were marked with asterisks.

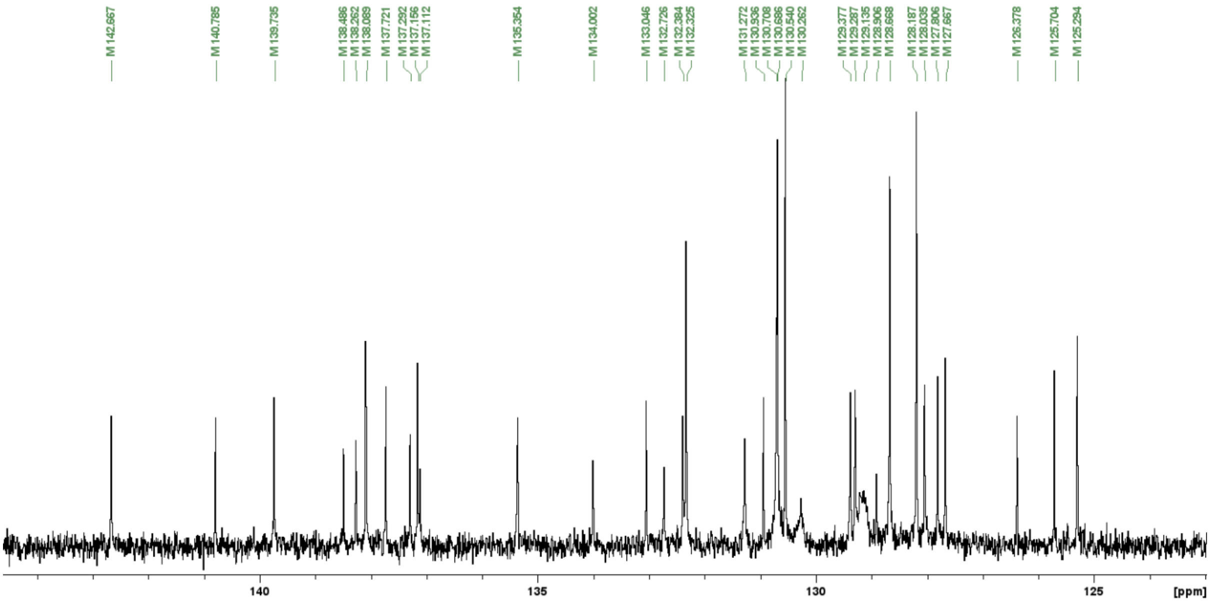

Figure S 38. Part of the ^{13}C NMR spectrum of **3-Se** (151 MHz, chloroform-*d*, 300 K).

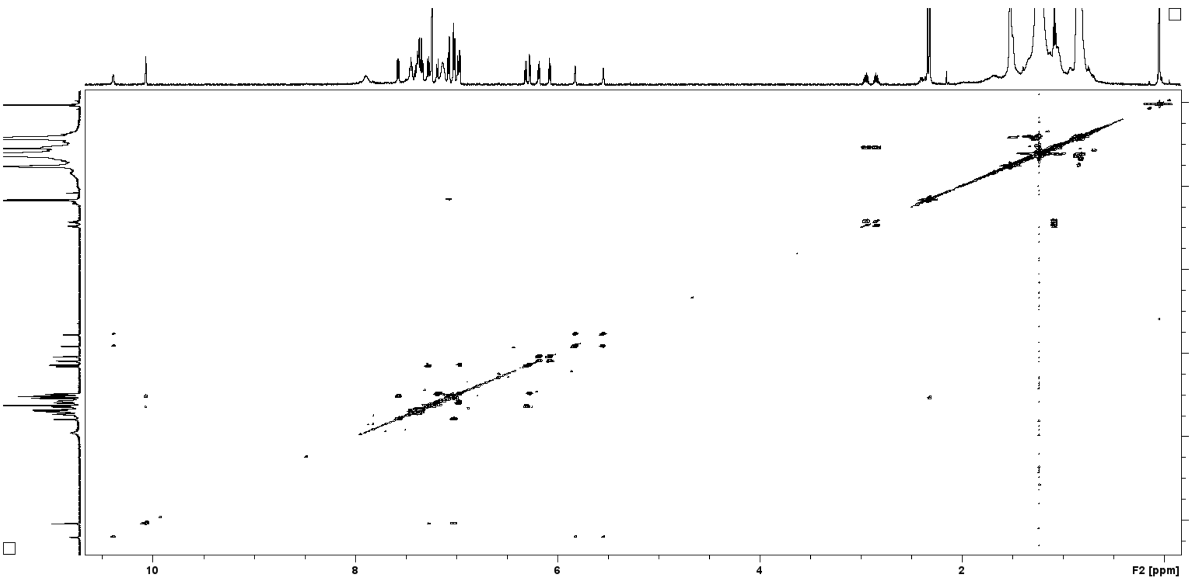

Figure S 39. The ^{1}H-^{1}H COSY NMR spectrum of **3-Se** (600 MHz, chloroform-*d*, 300 K).

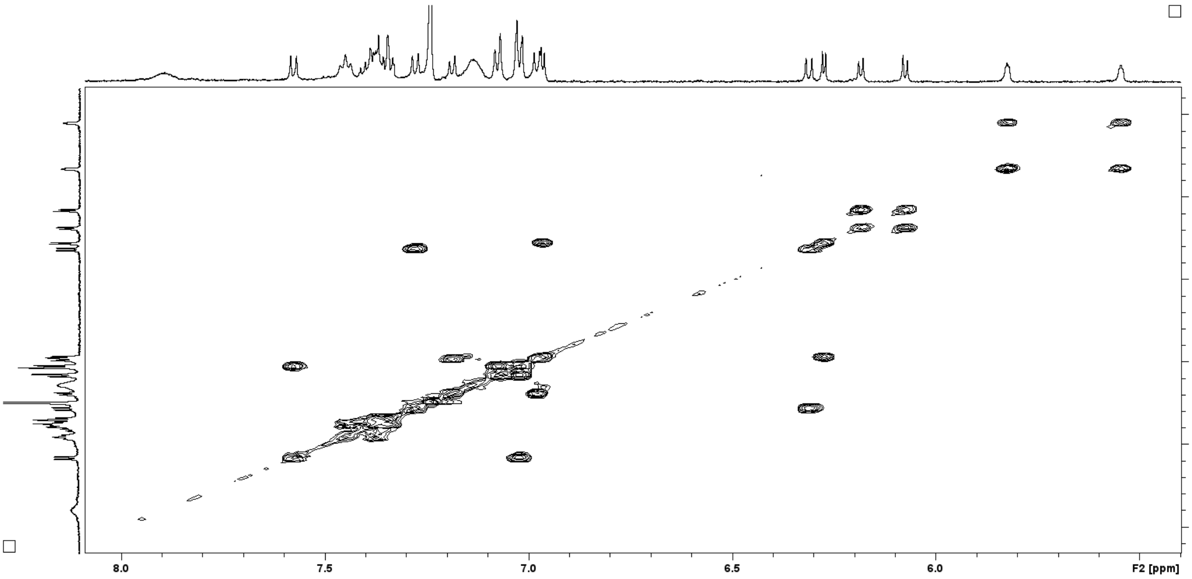

Figure S 40. Part of the ^{1}H-^{1}H COSY NMR spectrum of **3-Se** (600 MHz, chloroform-*d*, 300 K).

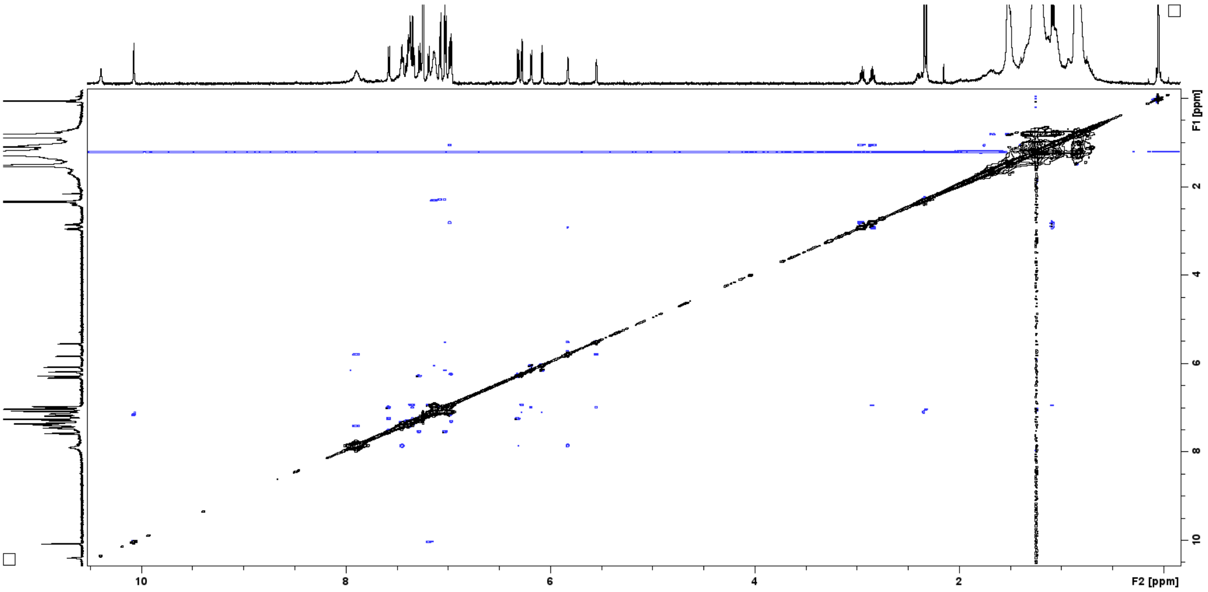

Figure S 41. The ^{1}H-^{1}H ROESY NMR spectrum of **3-Se** (600 MHz, dichloromethane-*d*$_2$, 300 K).

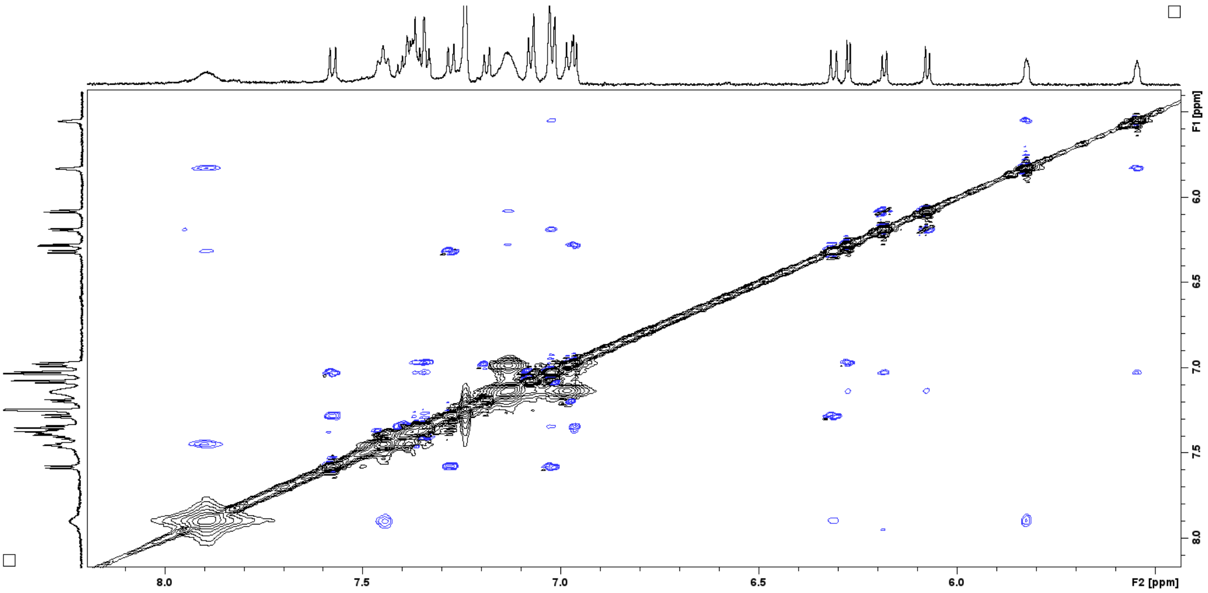

Figure S 42. Part of the ^{1}H-^{1}H ROESY NMR spectrum of **3-Se** (600 MHz, dichloromethane-*d*$_2$, 300 K).

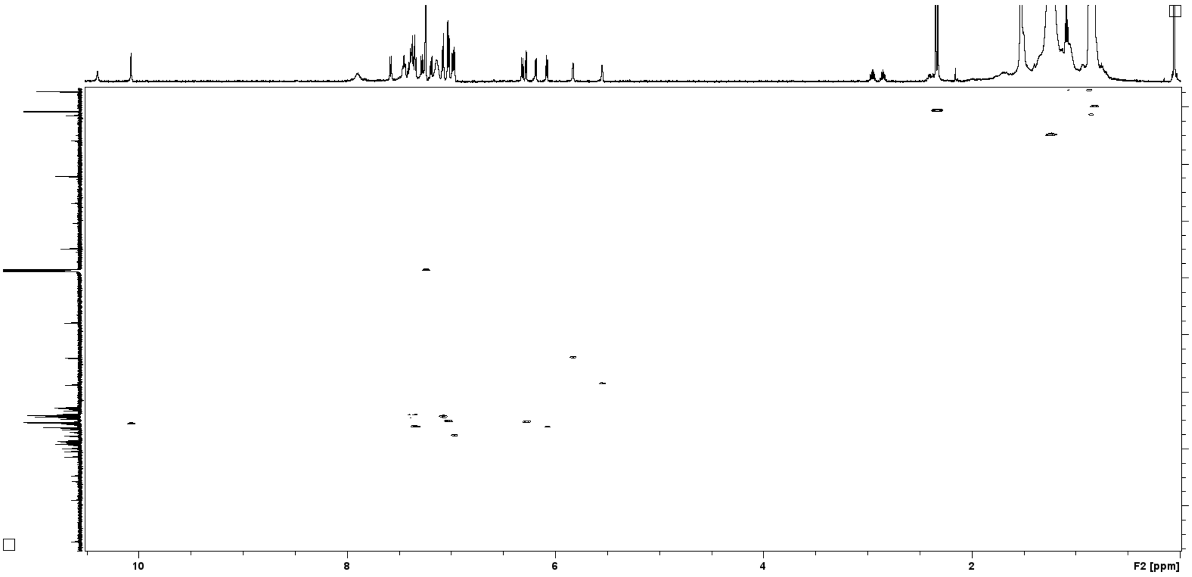

Figure S 43. The ^{1}H-^{13}C HMQC NMR spectrum of **3-Se** (600 MHz, chloroform-*d*, 300 K).

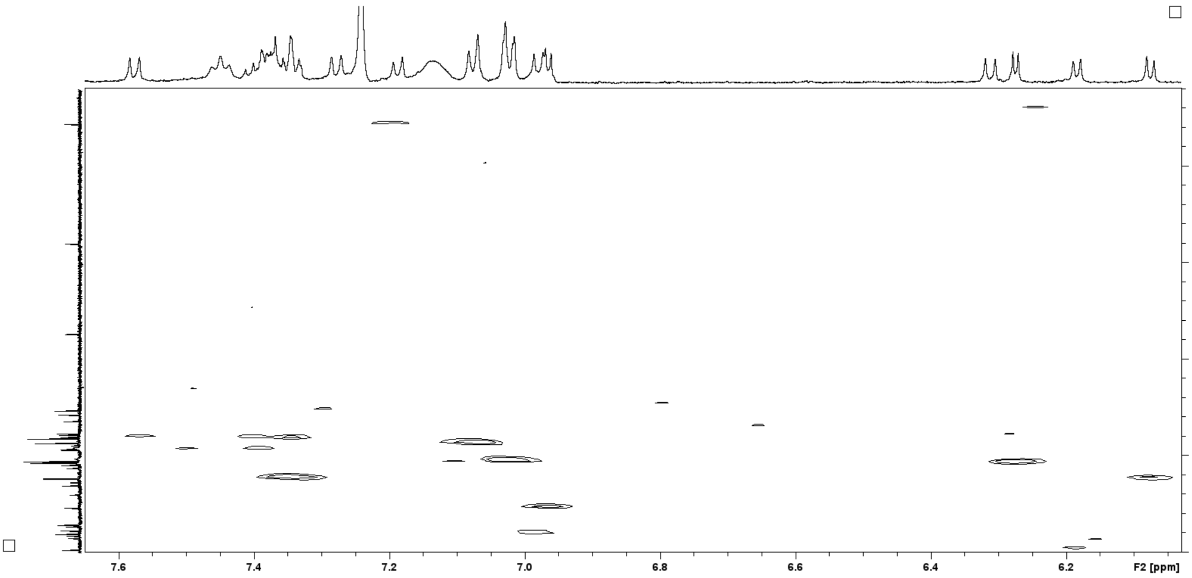

Figure S 44. Part of the ^{1}H-^{13}C HMQC NMR spectrum of **3-Se** (600 MHz, chloroform-*d*, 300 K).

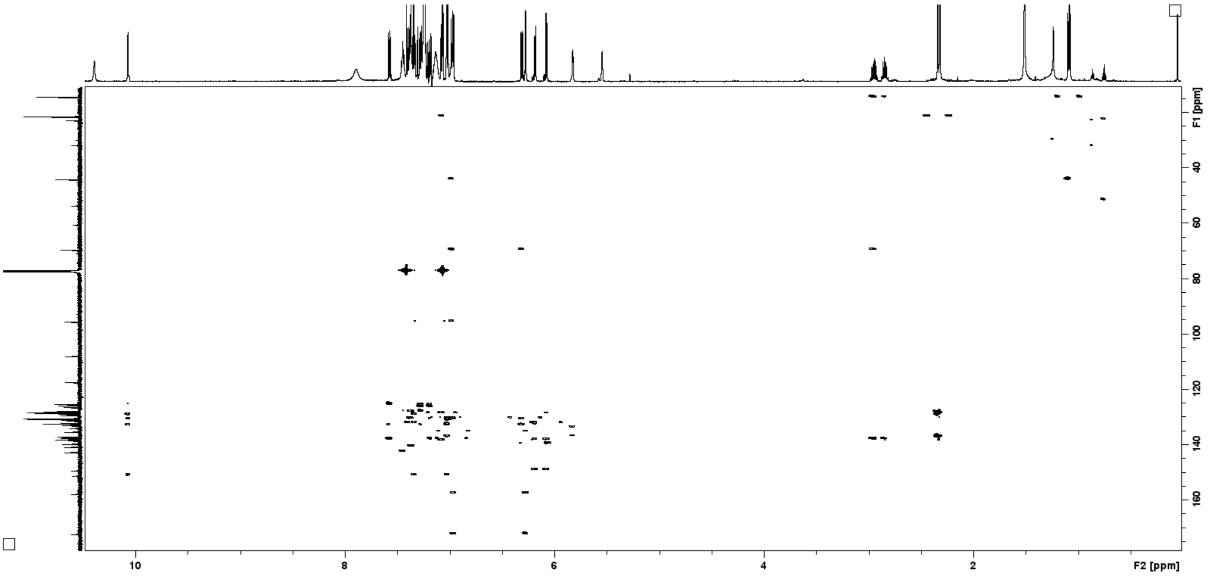

Figure S 45. The ^{1}H-^{13}C HMBC NMR spectrum of **3-Se** (600 MHz, chloroform-*d*, 300 K).

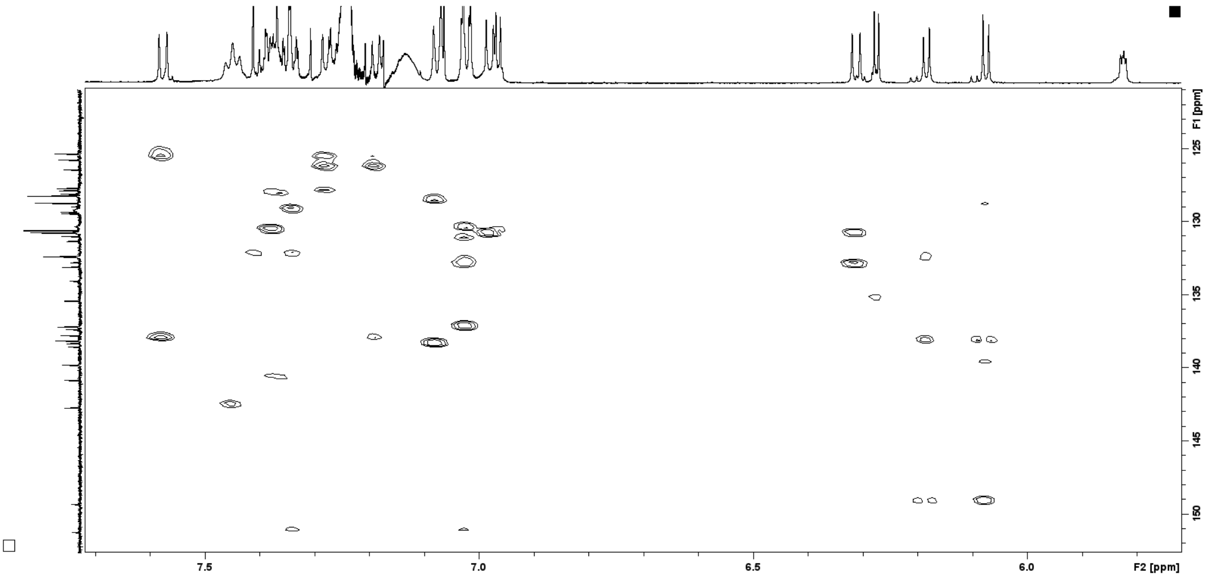

Figure S 46. Part of the ^{1}H-^{13}C HMBC NMR spectrum of **3-Se** (600 MHz, chloroform-*d*, 300 K).

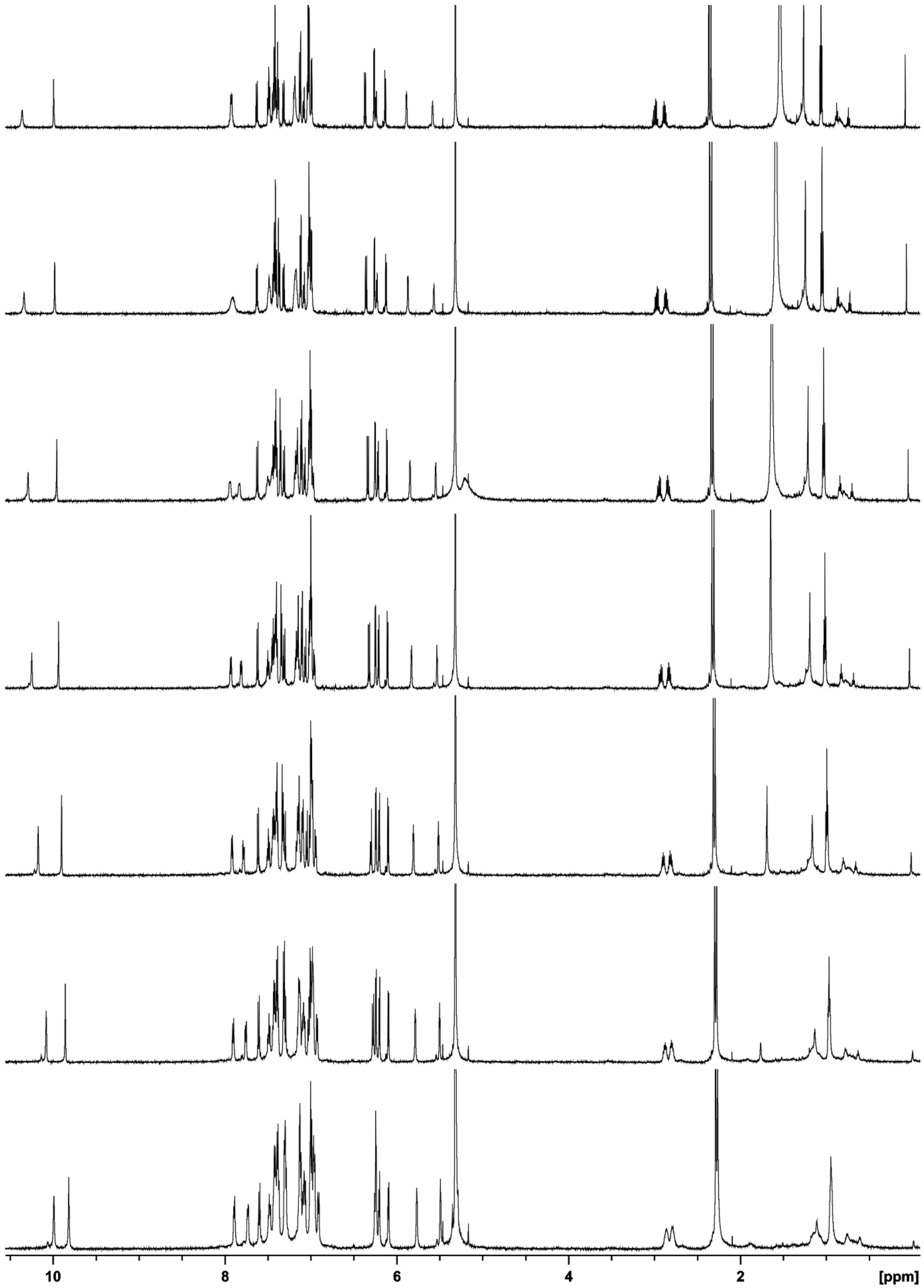

Figure S 47. The ^{1}H NMR spectra of **3-Se** recorded every 20 K in the 300 K (top) – 180 K (bottom) temperature range (600 MHz, dichloromethane-d_2).

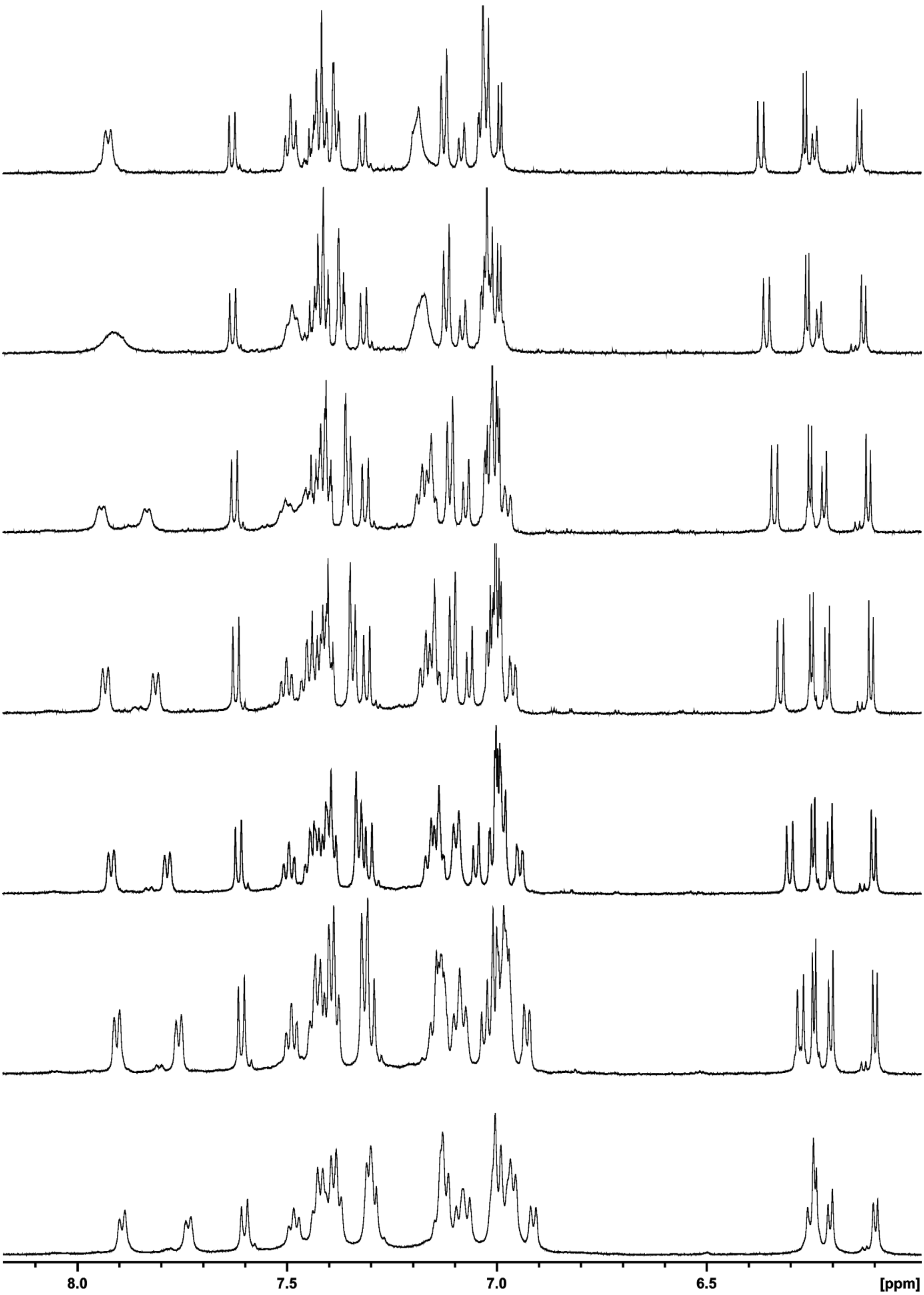

Figure S 48. Part of the ^{1}H NMR spectra of **3-Se** recorded every 20 K In the 300 K (top) – 180 K (bottom) temperature range (600 MHz, dichloromethane-d_2).

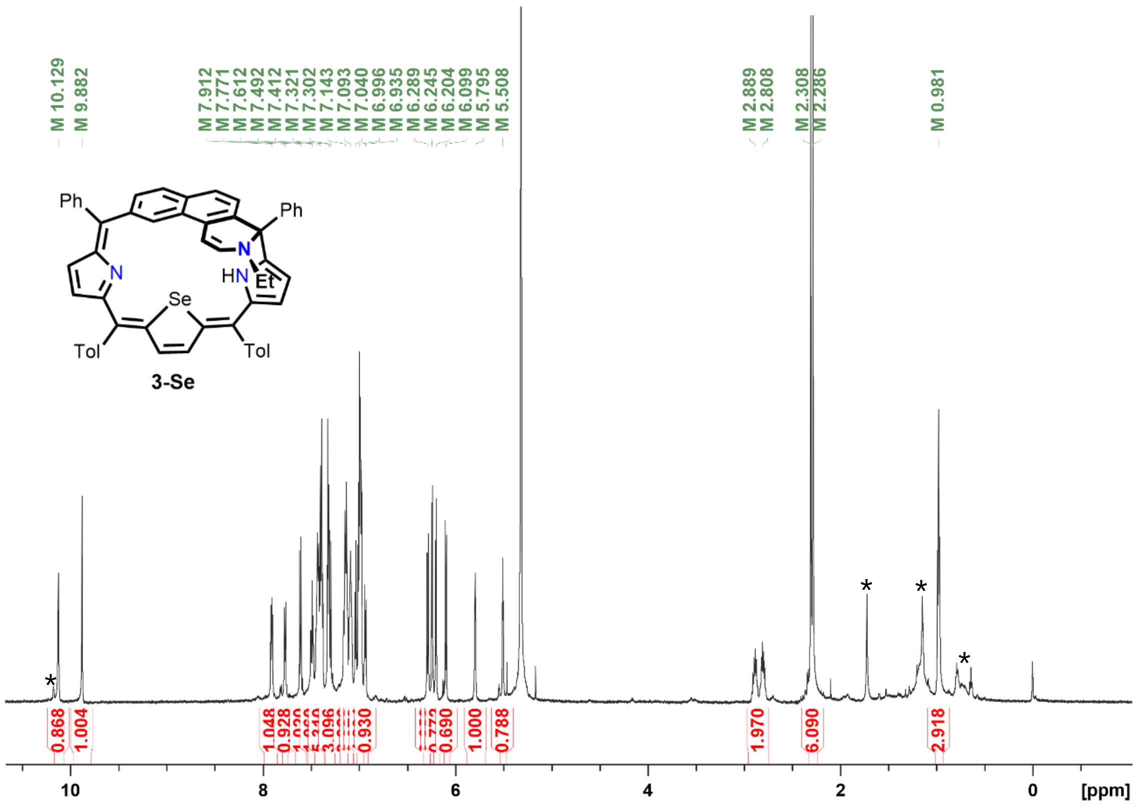

Figure S 49. The ^{1}H NMR spectrum of **3-Se** (600 MHz, dichloromethane-d_2, 210 K). Impurities were marked with asterisks.

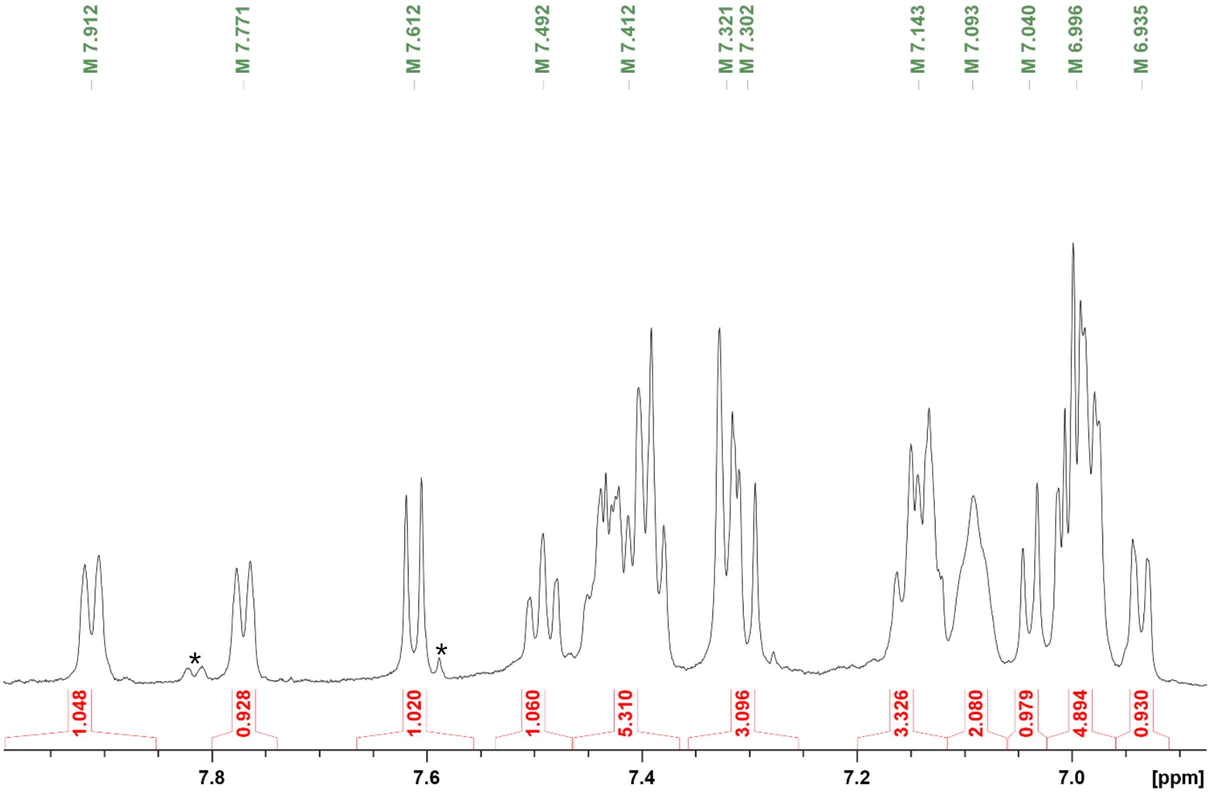

Figure S 50. Part of the ^{1}H NMR spectrum of **3-Se** (600 MHz, dichloromethane-d_2, 210 K). Impurities were marked with asterisks.

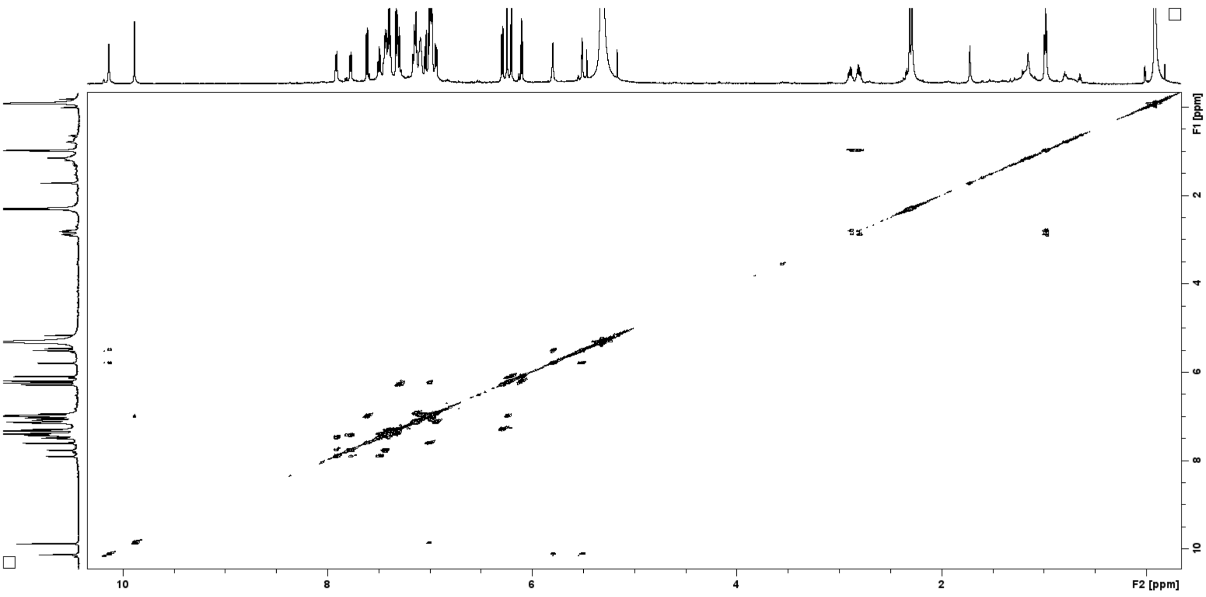

Figure S 51. The ^{1}H-^{1}H COSY NMR spectrum of **3-Se** (600 MHz, dichloromethane-*d*$_2$, 210 K).

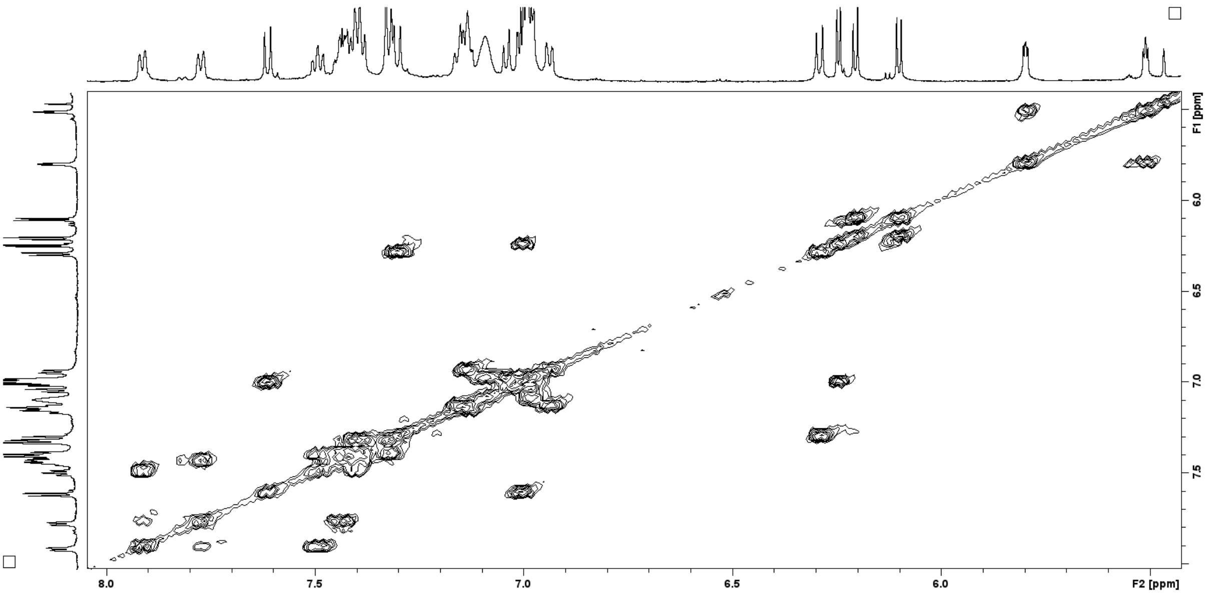

Figure S 52. Part of the ^{1}H-^{1}H COSY NMR spectrum of **3-Se** (600 MHz, dichloromethane-*d*$_2$, 210 K).

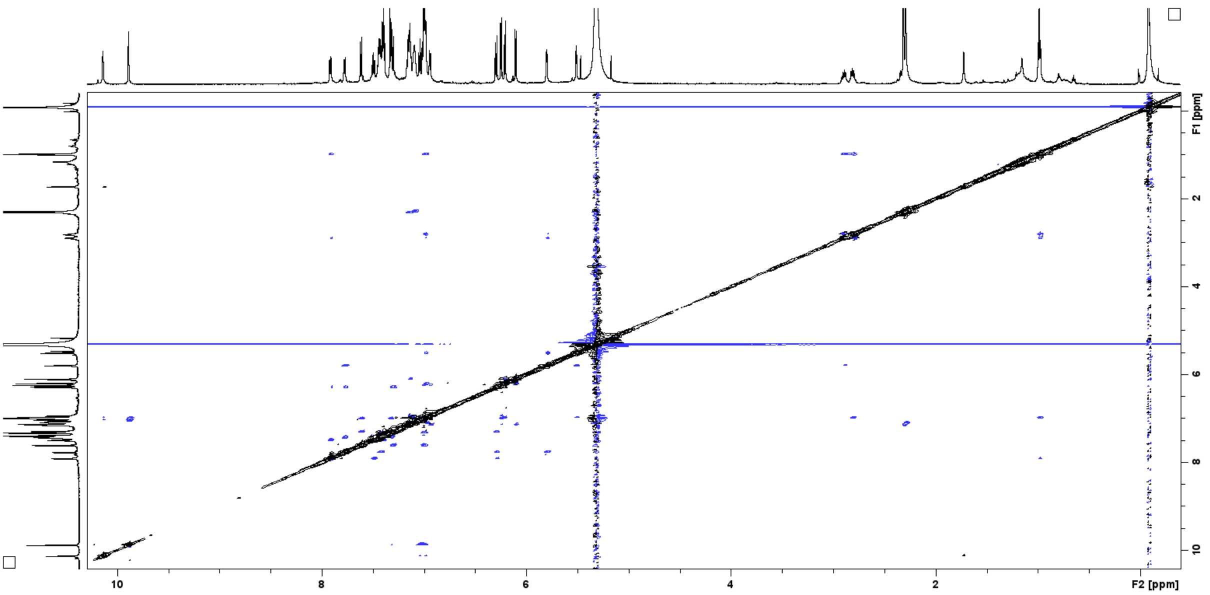

Figure S 53. The ^{1}H-^{1}H ROESY NMR spectrum of **3-Se** (600 MHz, dichloromethane-*d*$_2$, 210 K).

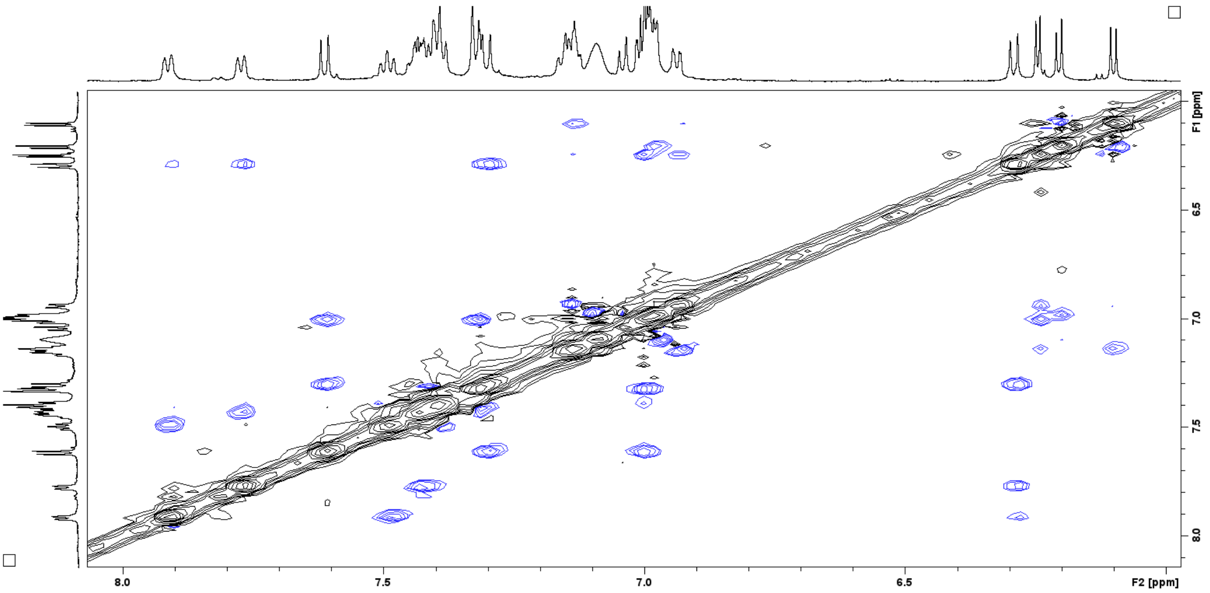

Figure S 54. Part of the ^{1}H-^{1}H ROESY NMR spectrum of **3-Se** (600 MHz, dichloromethane-*d*$_2$, 210 K).

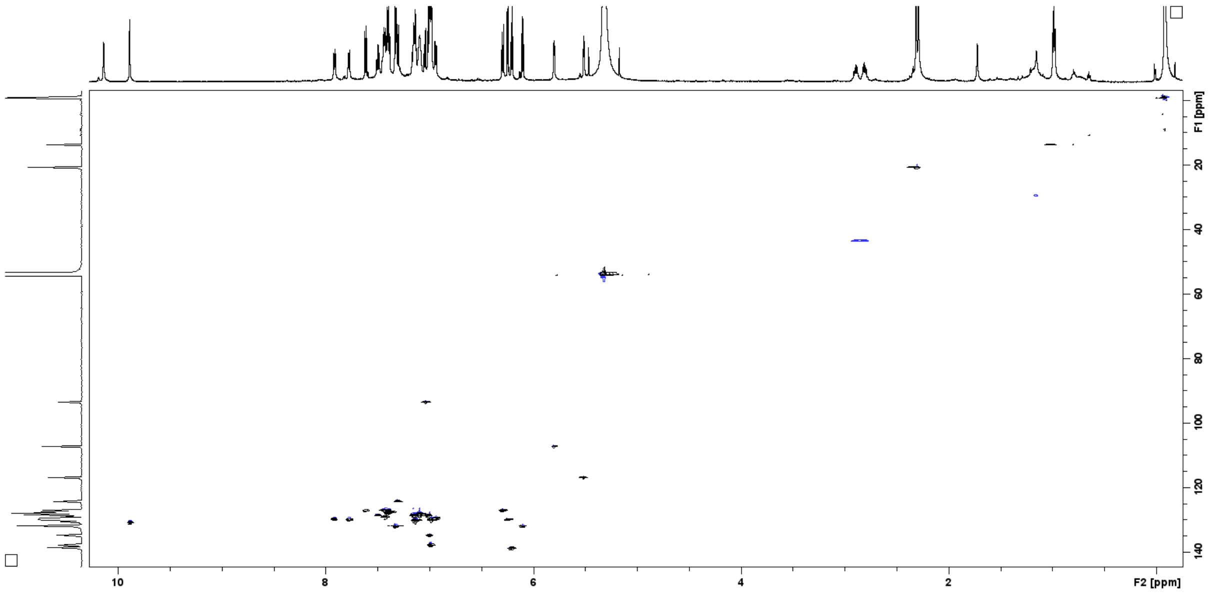

Figure S 55. The ^{1}H-^{13}C HSQC NMR spectrum of **3-Se** (600 MHz, dichloromethane-d_2, 210 K).

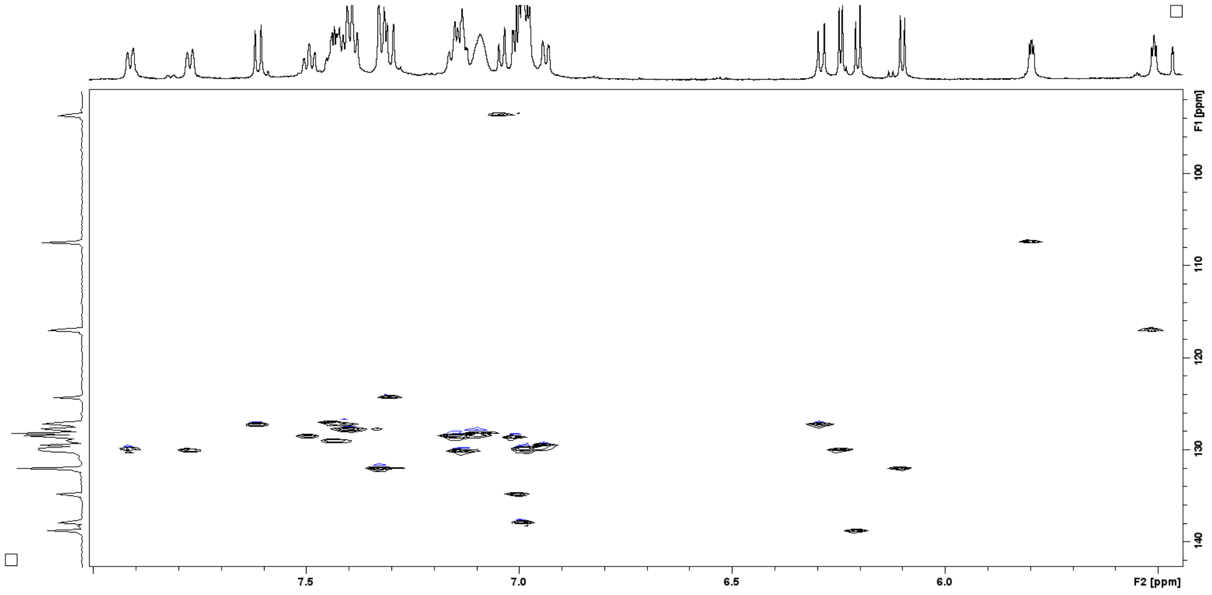

Figure S 56. Part of the ^{1}H-^{13}C HSQC NMR spectrum of **3-Se** (600 MHz, dichloromethane-d_2, 210 K).

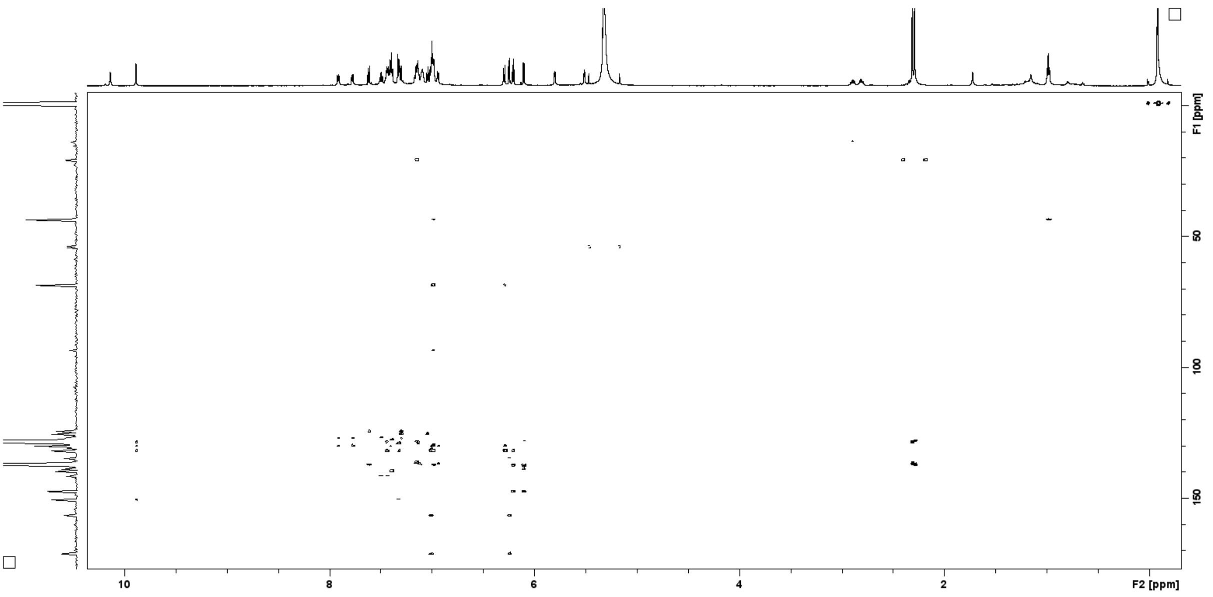

Figure S 57. The ^{1}H-^{13}C HMBC NMR spectrum of **3-Se** (600 MHz, dichloromethane-*d*2, 210 K).

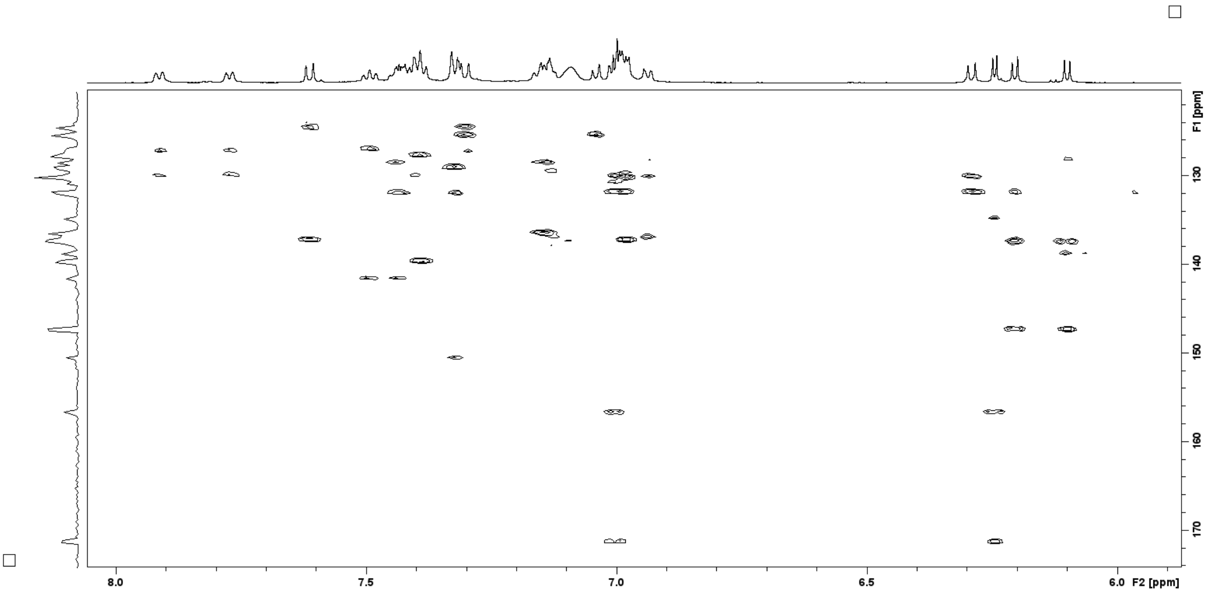

Figure S 58. Part of the ^{1}H-^{13}C HMBC NMR spectrum of **3-Se** (600 MHz, dichloromethane-*d*2, 210 K).

NMR spectra of macrocycle 4-Se

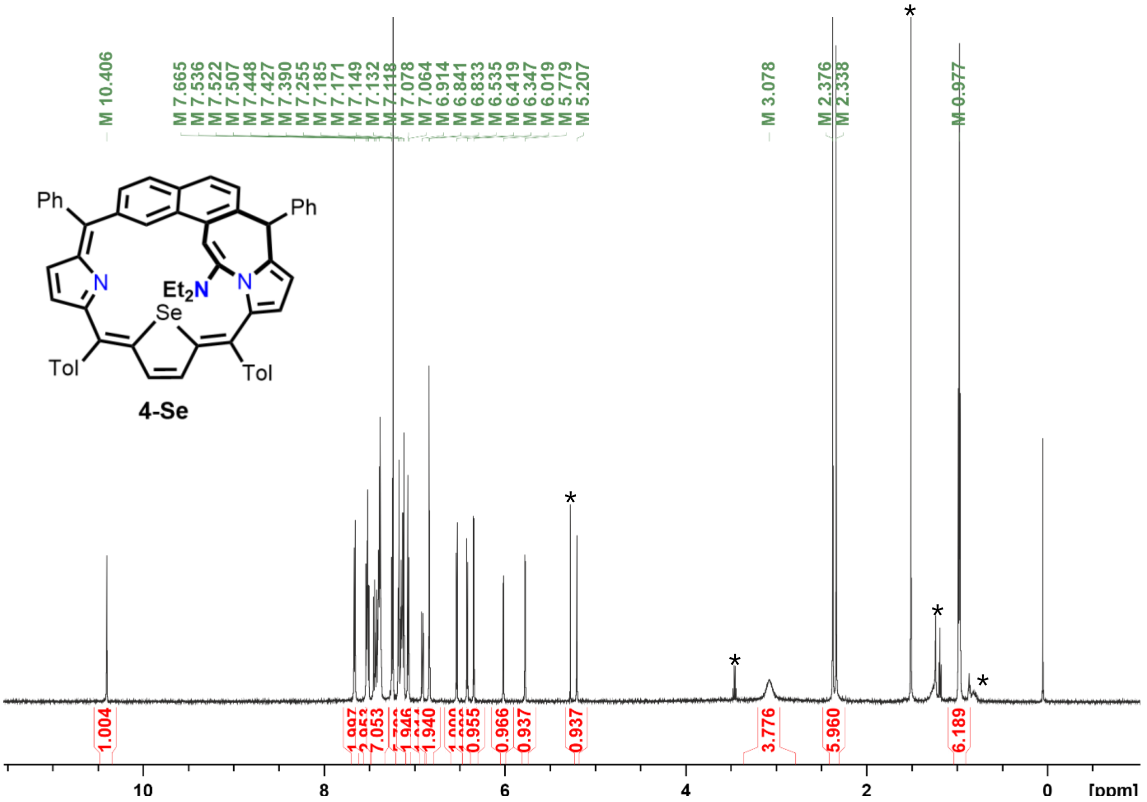

Figure S 59. The ^{1}H NMR spectrum of **4-Se** (600 MHz, chloroform-*d*, 300 K). Impurities were marked with asterisks.

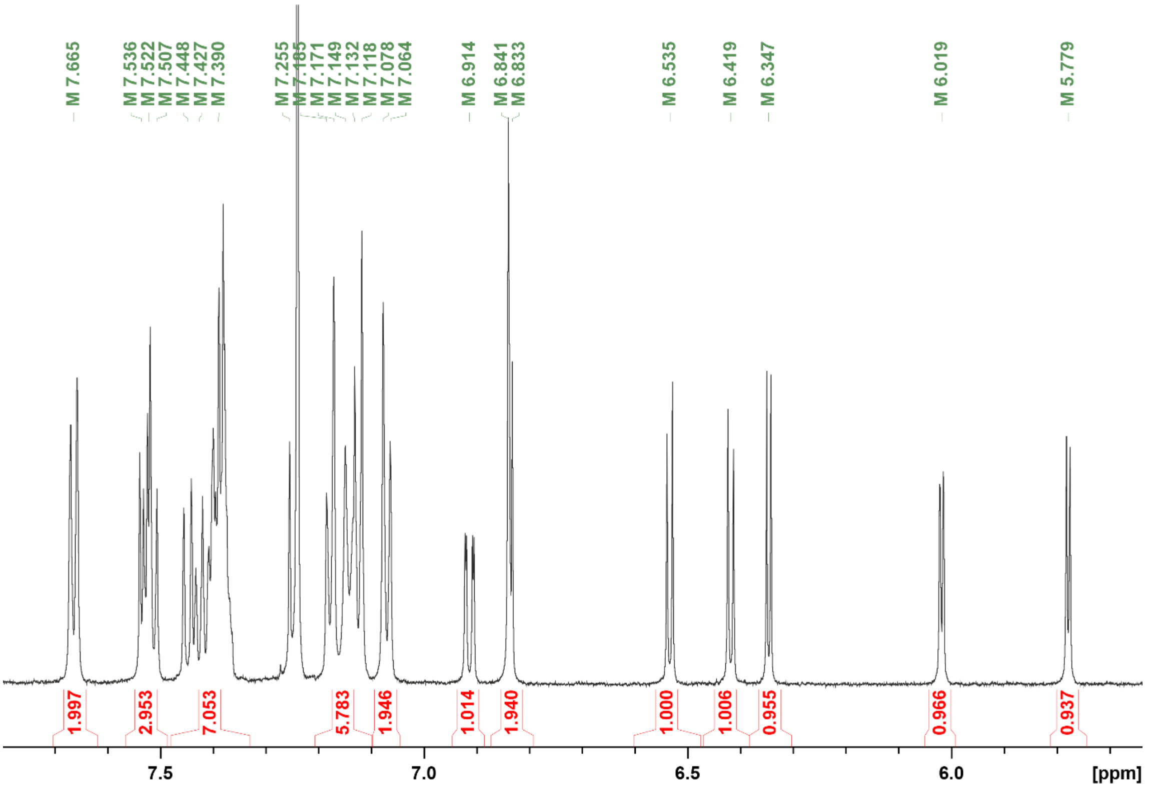

Figure S 60. Part of the ^{1}H NMR spectrum of **4-Se** (600 MHz, chloroform-*d*, 300 K).

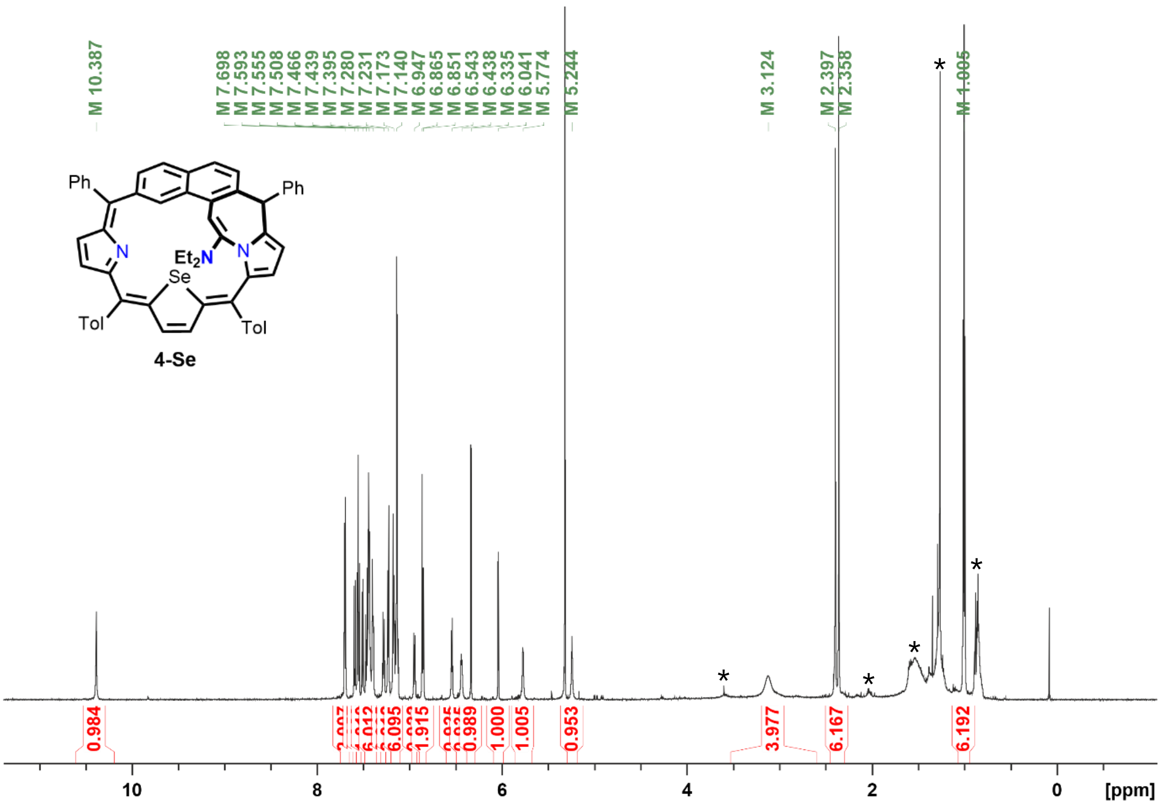

Figure S 61. The ^{1}H NMR spectrum of **4-Se** (600 MHz, dichloromethane-d_2, 300 K). Impurities were marked with asterisks.

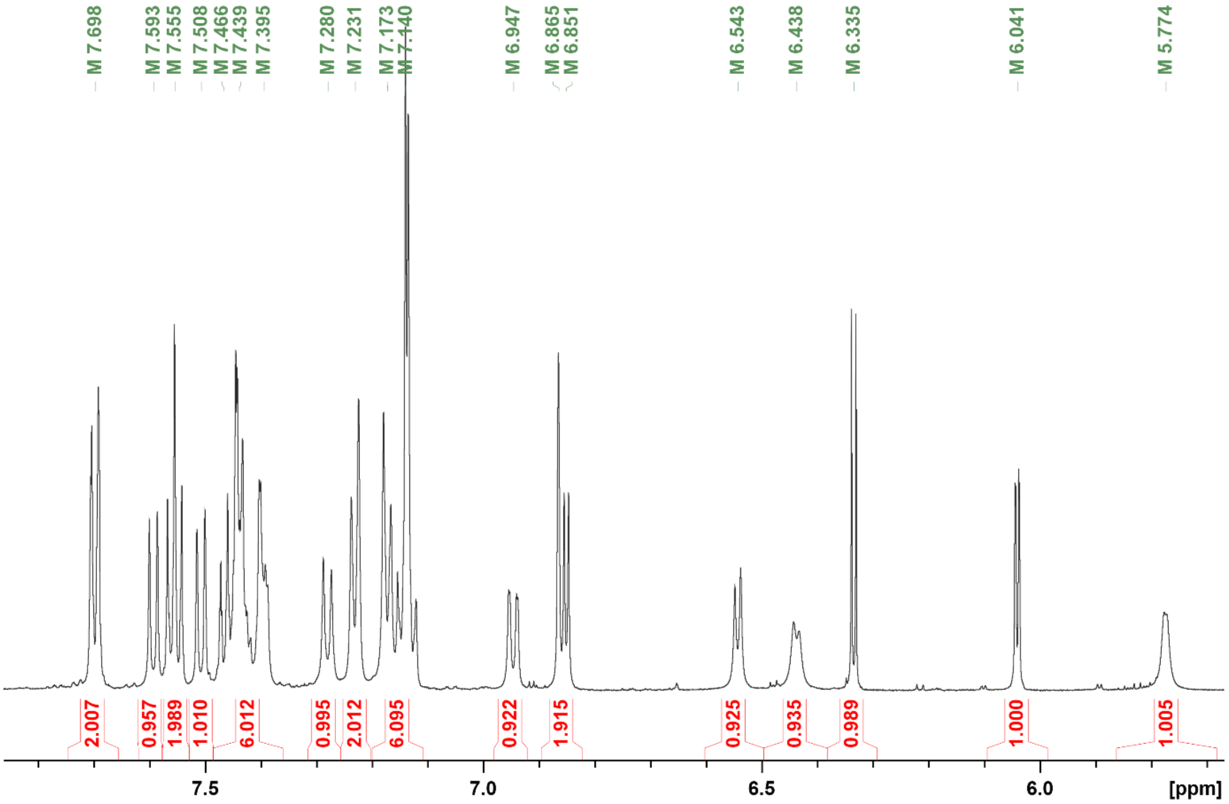

Figure S 62. Part of the ^{1}H NMR spectrum of **4-Se** (600 MHz, dichloromethane-d_2, 300 K).

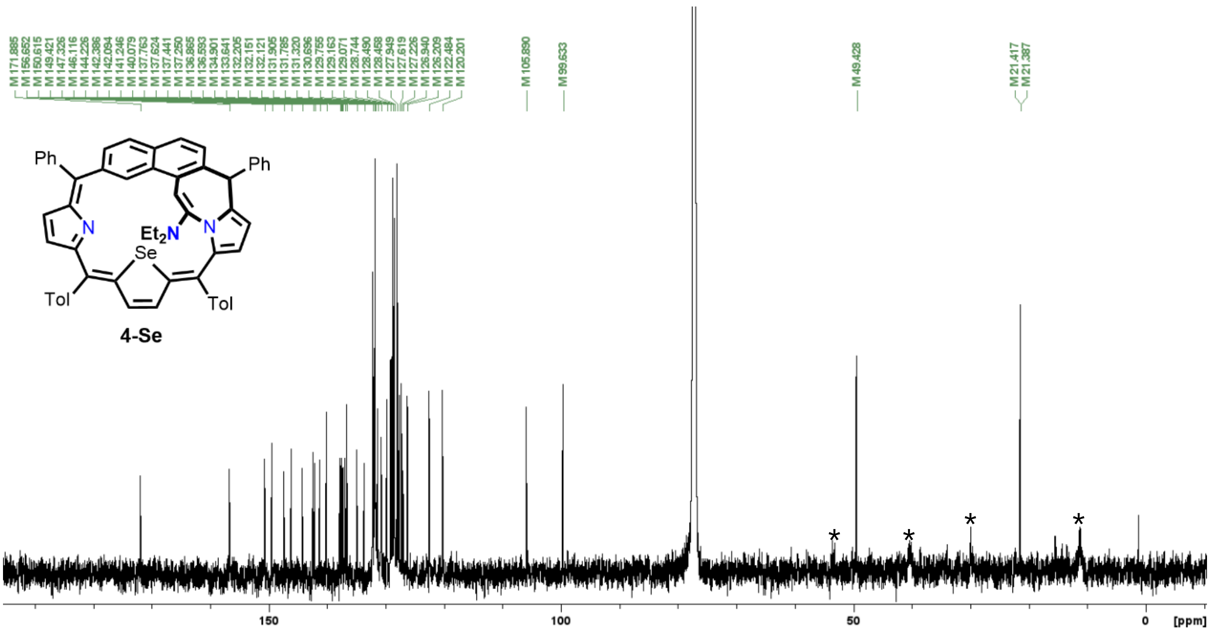

Figure S 63. The ^{13}C NMR spectrum of **4-Se** (151 MHz, chloroform-*d*, 300 K). Impurities were marked with asterisks.

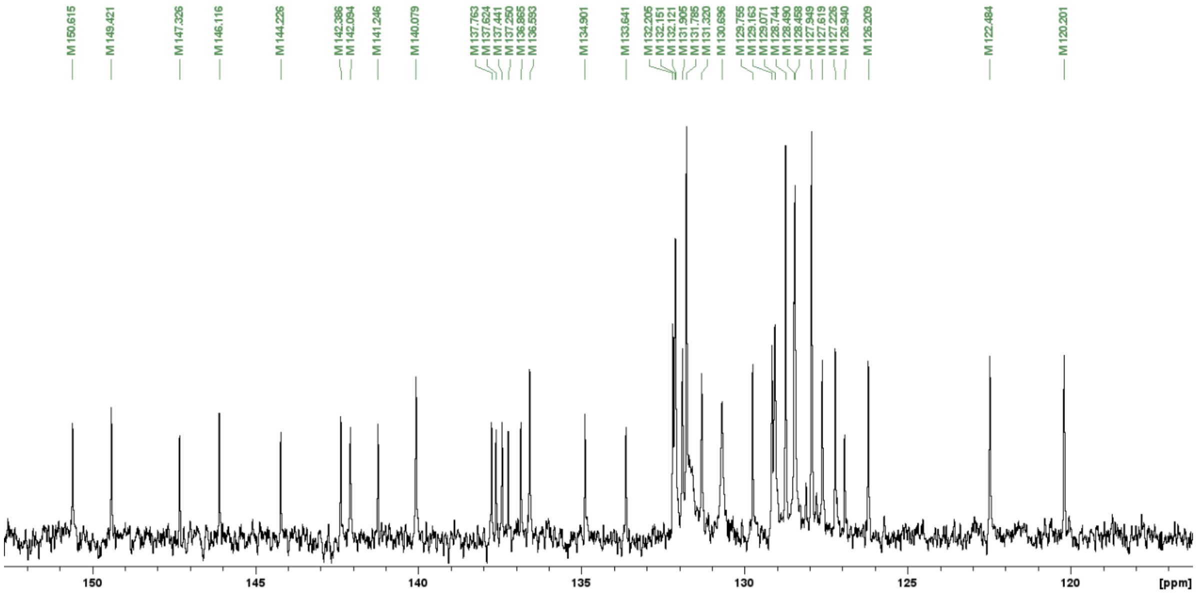

Figure S 64. Part of the ^{13}C NMR spectrum of **4-Se** (151 MHz, chloroform-*d*, 300 K).

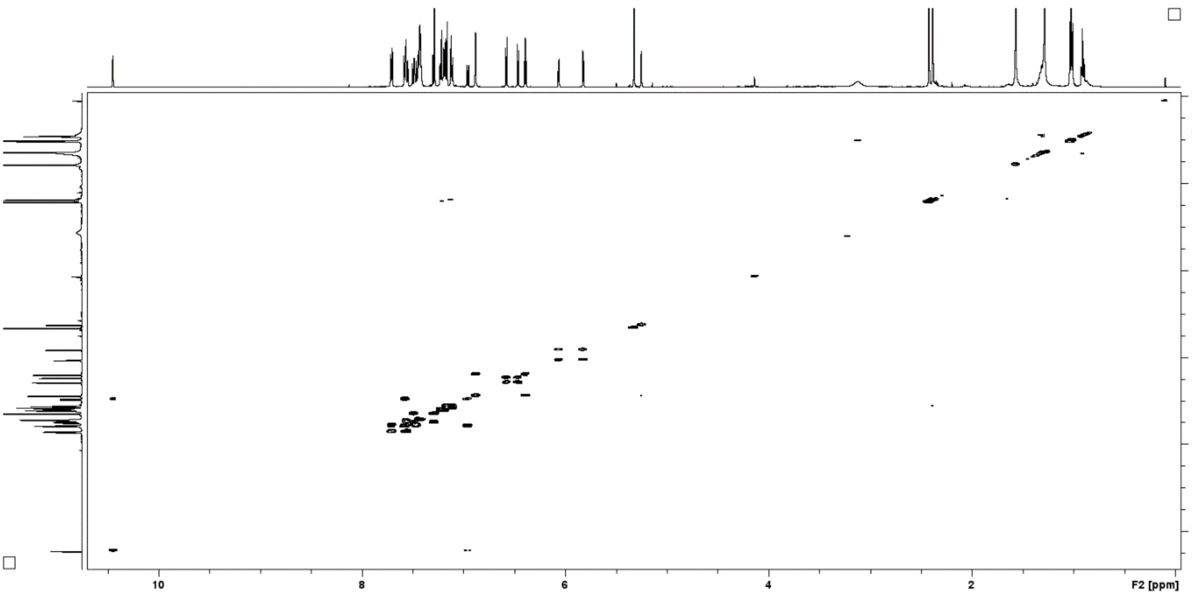

Figure S 65. The ^{1}H-^{1}H COSY NMR spectrum of **4-Se** (500 MHz, chloroform-*d*, 300 K).

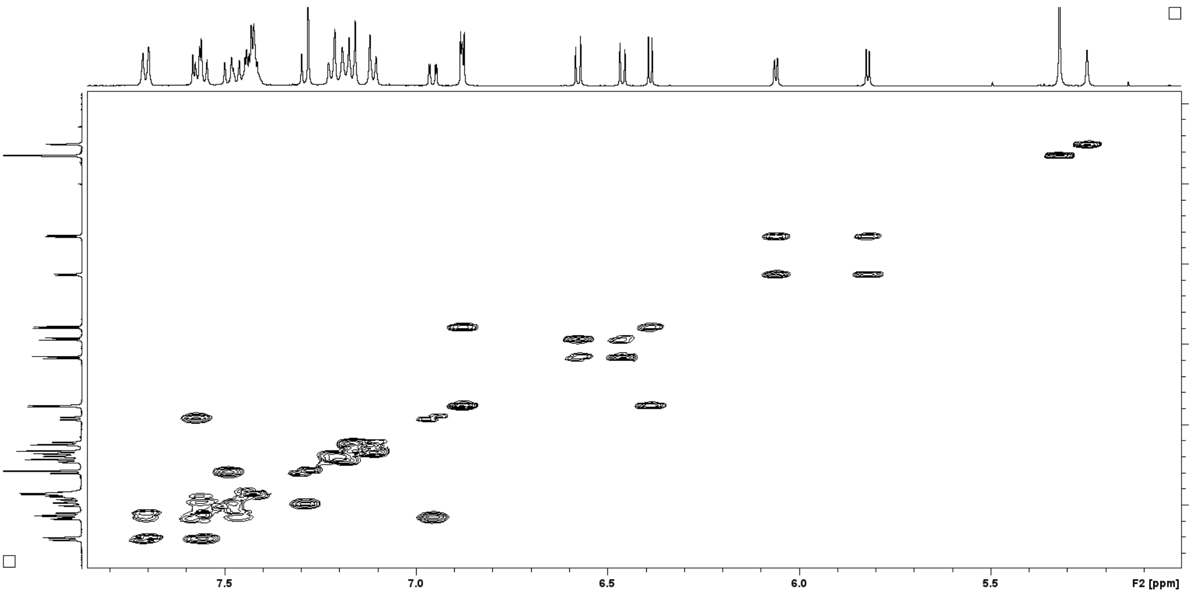

Figure S 66. Part of the ^{1}H-^{1}H COSY NMR spectrum of **4-Se** (500 MHz, chloroform-*d*, 300 K).

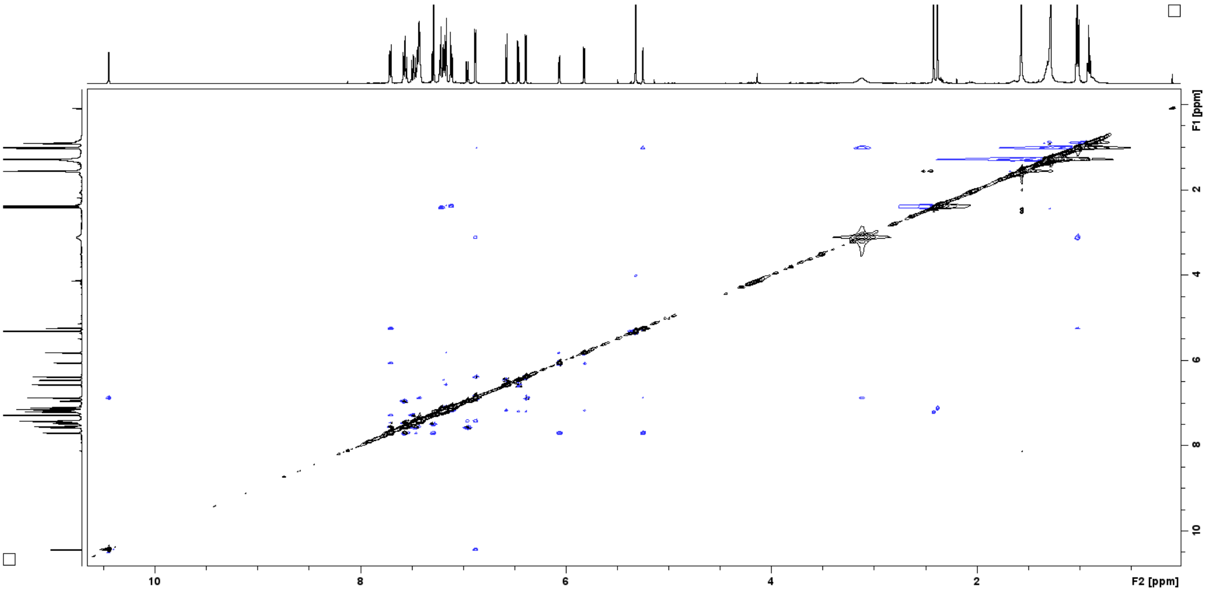

Figure S 67. The ^{1}H-^{1}H NOESY NMR spectrum of **4-Se** (500 MHz, chloroform-*d*, 300 K).

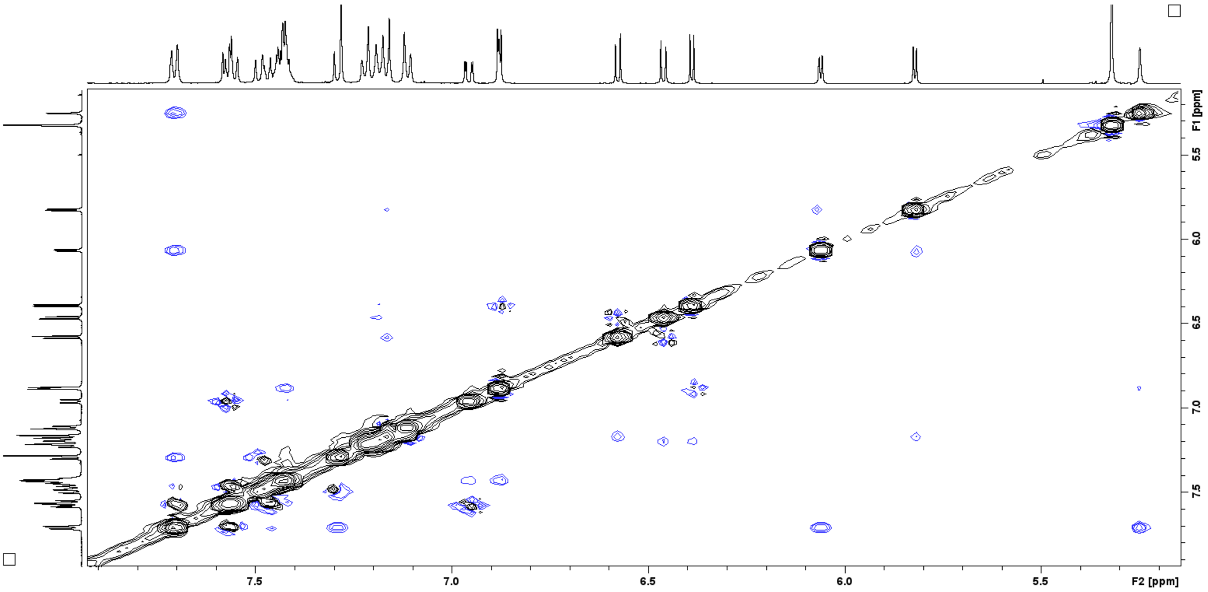

Figure S 68. Part of the ^{1}H-^{1}H NOESY spectrum of **4-Se** (500 MHz, chloroform-*d*, 300 K).

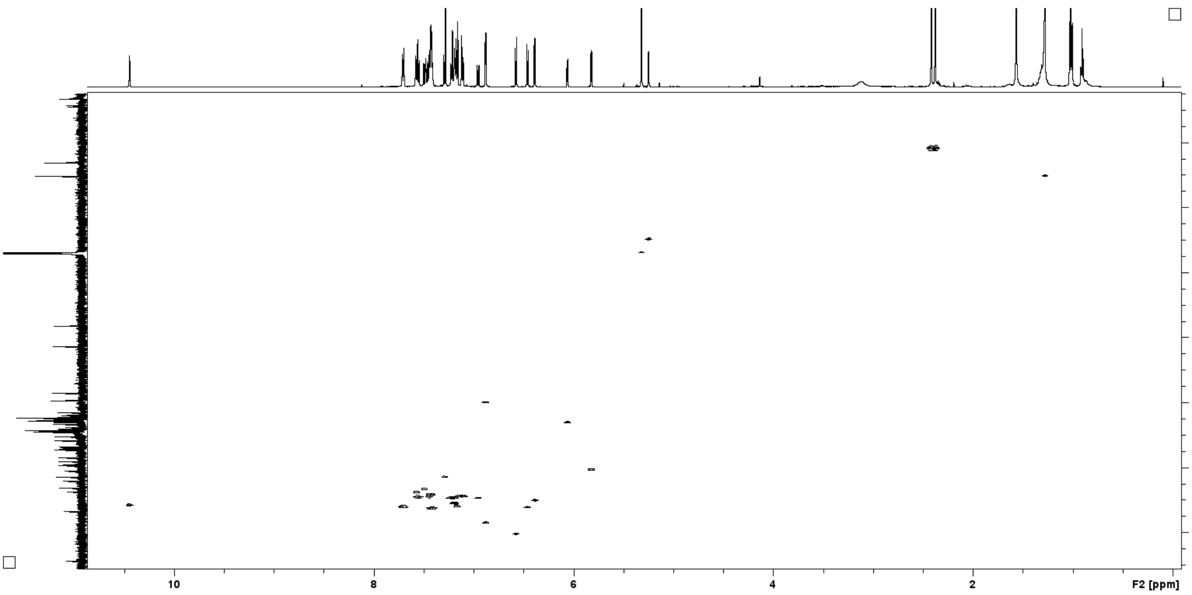

Figure S 69. The ^{1}H-^{13}C HMQC NMR spectrum of **4-Se** (500 MHz, chloroform-*d*, 300 K).

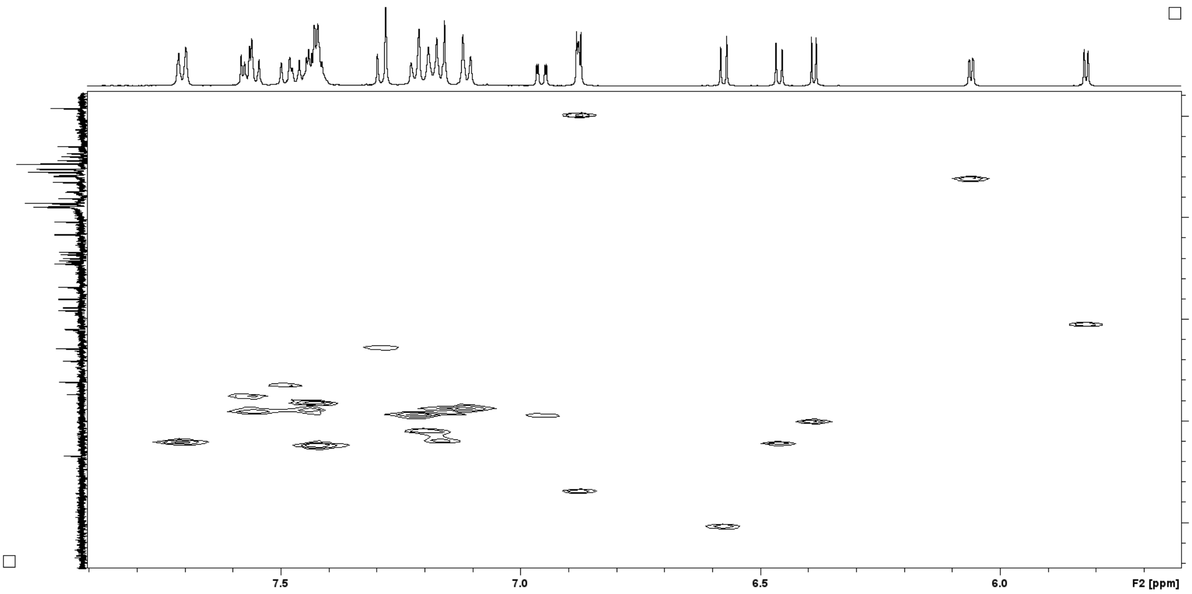

Figure S 70. Part of the ^{1}H-^{13}C HMQC NMR spectrum of **4-Se** (500 MHz, chloroform-*d*, 300 K).

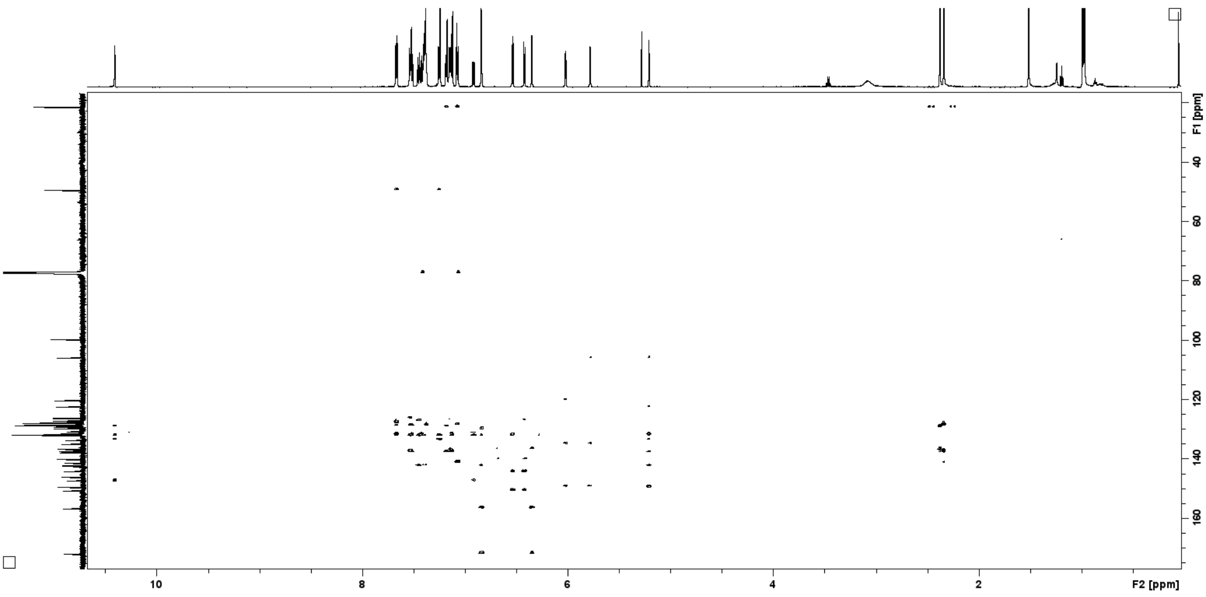

Figure S 71. The ^{1}H-^{13}C HMBC NMR spectrum of **4-Se** (600 MHz, chloroform-*d*, 300 K).

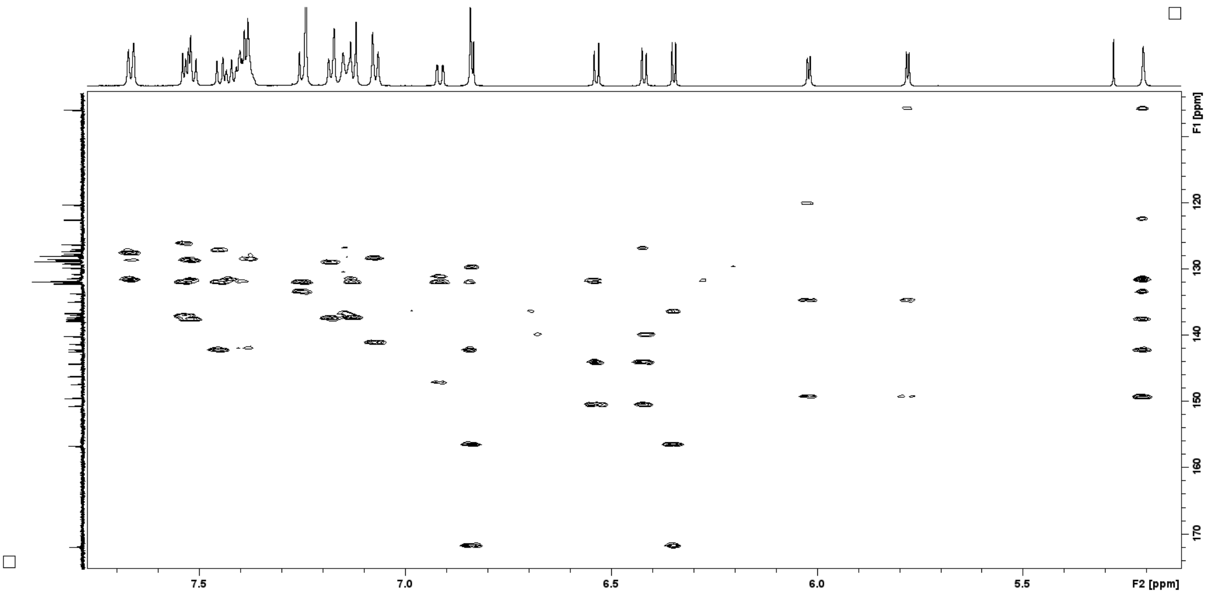

Figure S 72. Part of the ^{1}H-^{13}C HMBC NMR spectrum of **4-Se** (600 MHz, chloroform-*d*, 300 K).

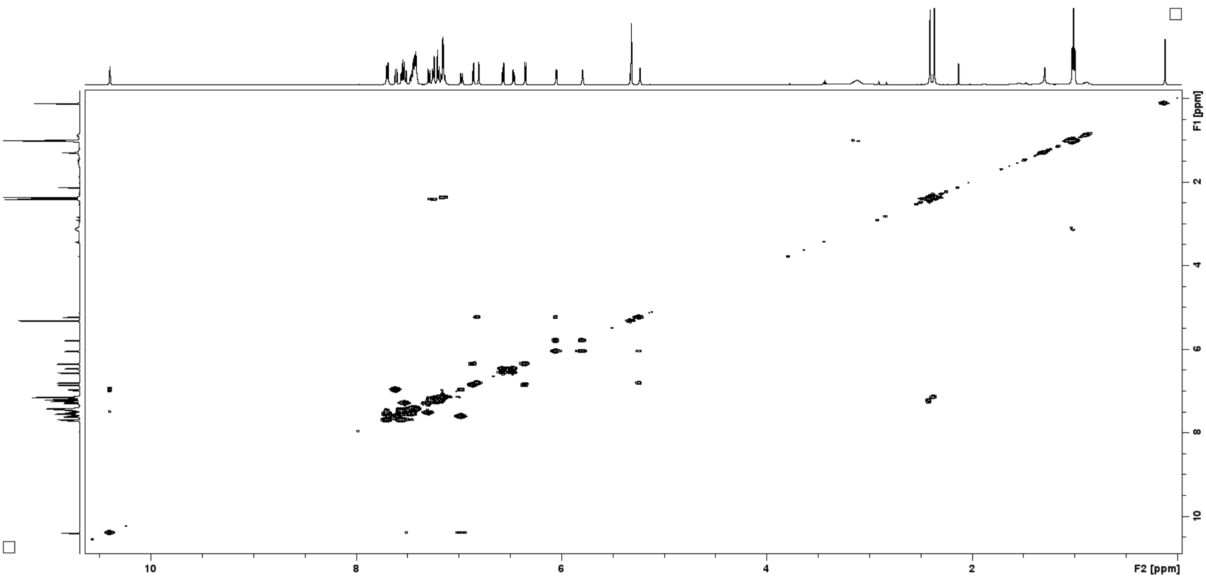

Figure S 73. The ^{1}H-^{1}H COSY NMR spectrum of **4-Se** (500 MHz, dichloromethane-*d*$_2$, 300 K).

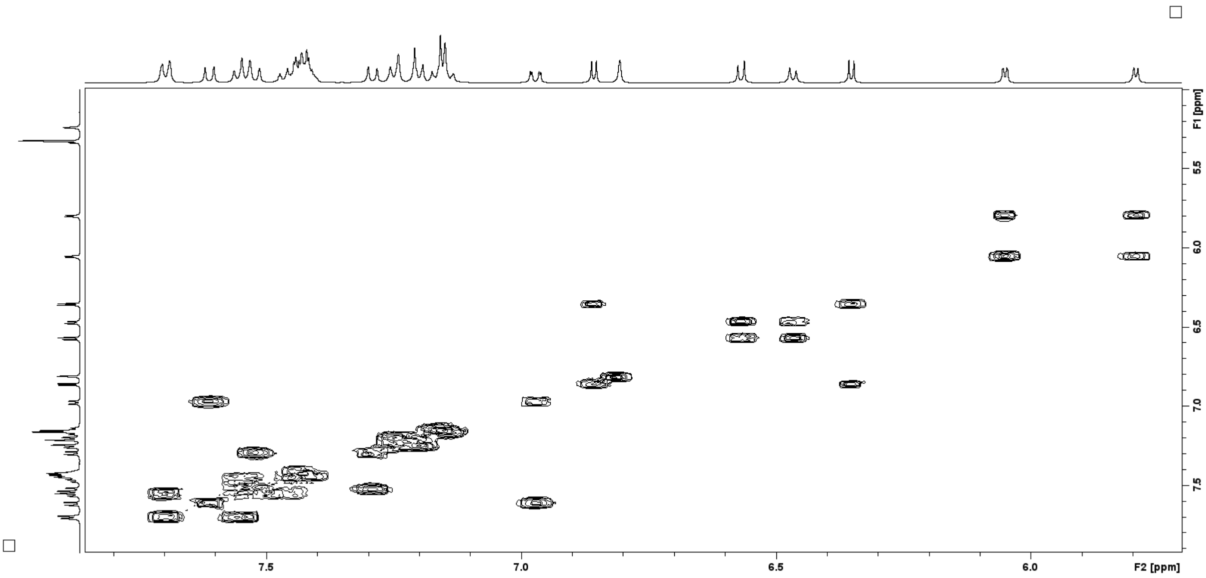

Figure S 74. Part of the ^{1}H-^{1}H COSY NMR spectrum of **4-Se** (500 MHz, dichloromethane-*d*$_2$, 300 K).

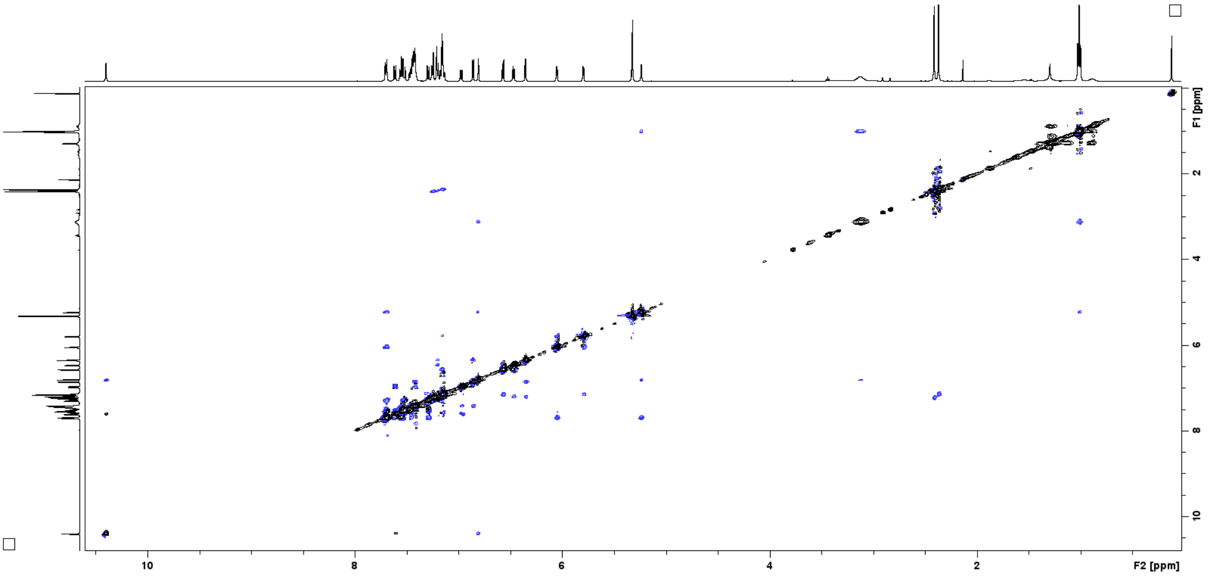

Figure S 75. The ^{1}H-^{1}H ROESY NMR spectrum of **4-Se** (500 MHz, dichloromethane-*d*$_2$, 300 K).

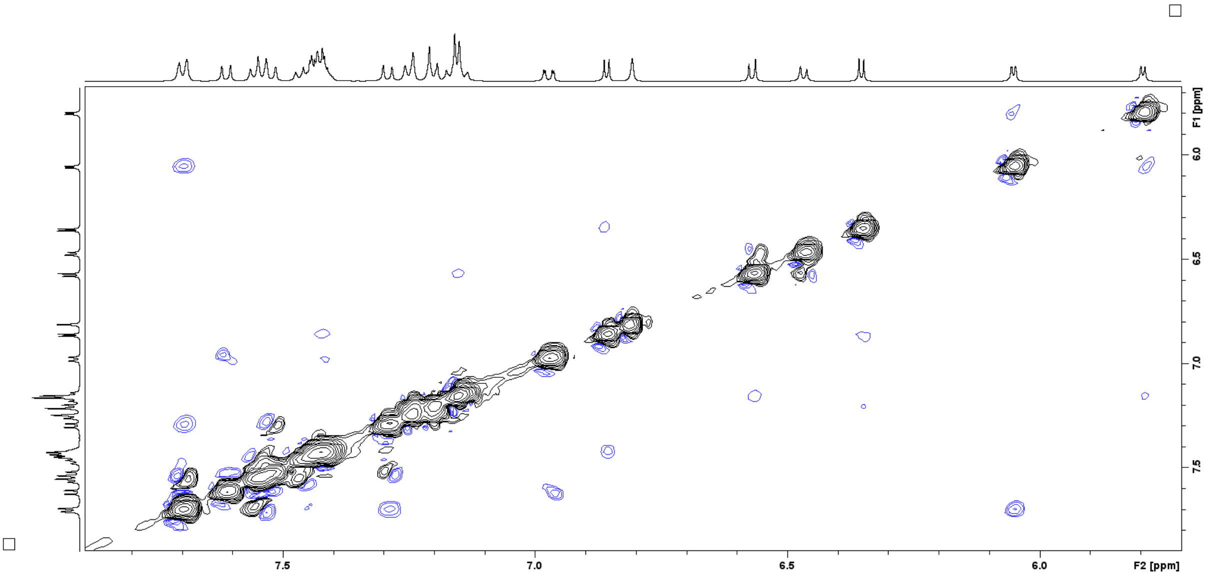

Figure S 76. Part of the ^{1}H-^{1}H ROESY NMR spectrum of **4-Se** (500 MHz, dichloromethane-*d*$_2$, 300 K).

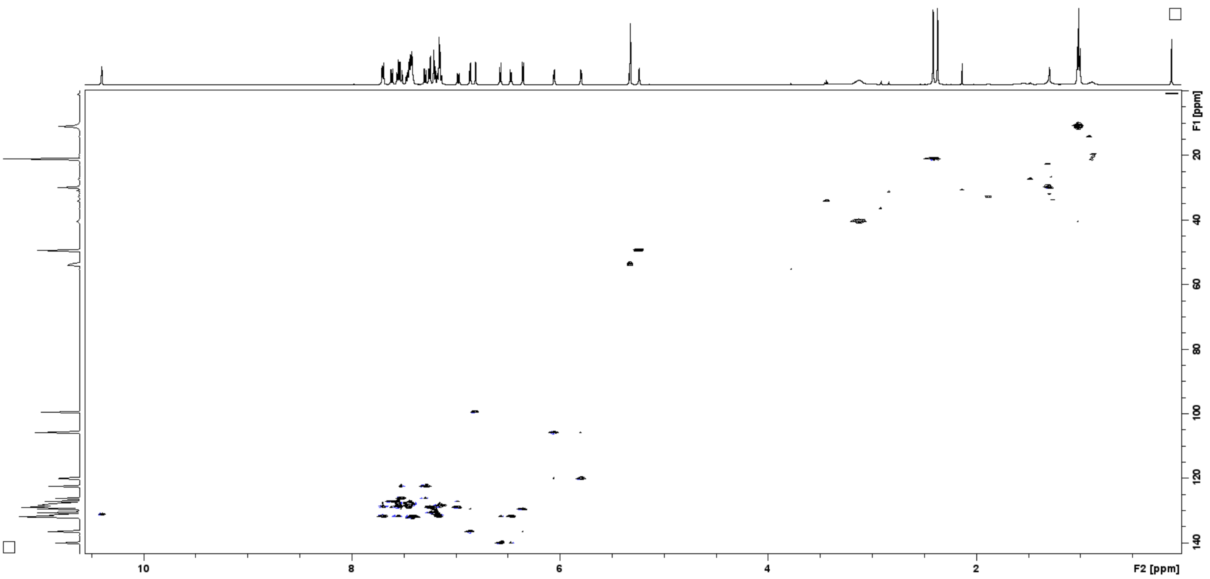

Figure S 77. The ^{1}H-^{13}C HSQC NMR spectrum of **4-Se** (500 MHz, dichloromethane-d_2, 300 K).

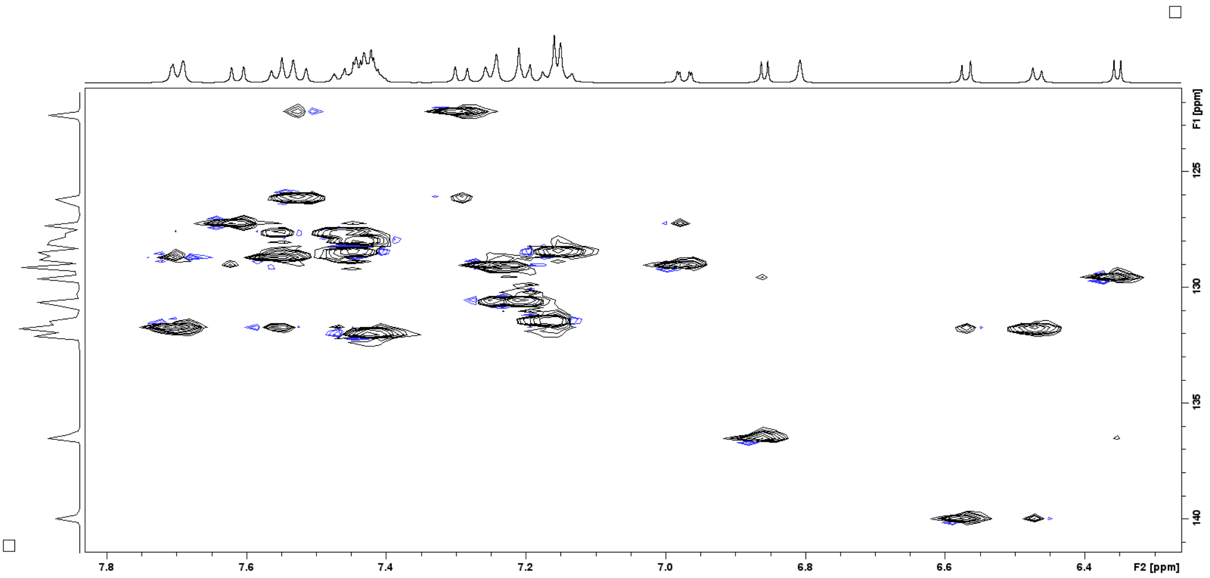

Figure S 78. Part of the ^{1}H-^{13}C HSQC NMR spectrum of **4-Se** (500 MHz, dichloromethane-d_2, 300 K).

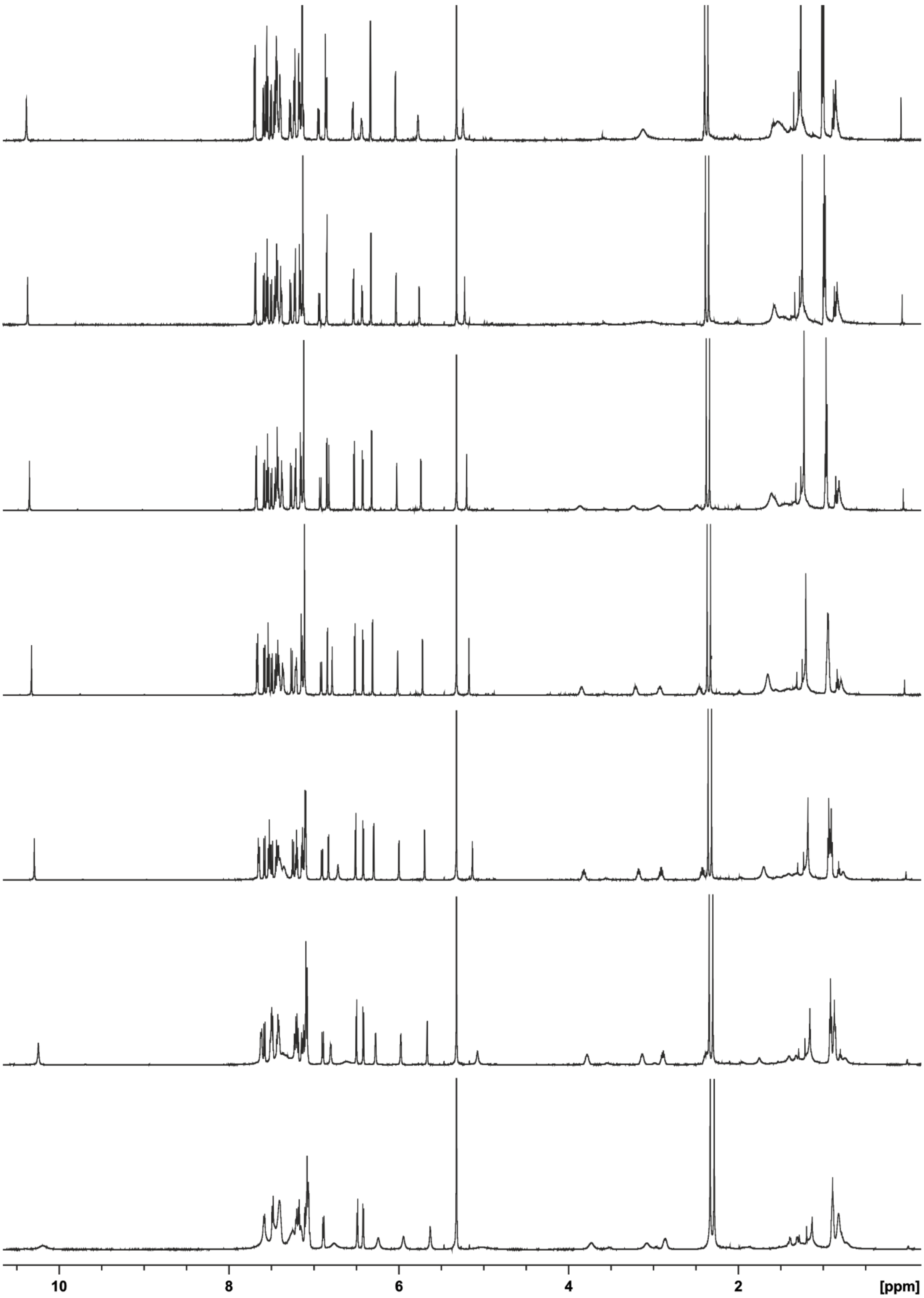

Figure S 79. The ^{1}H NMR spectra of **4-Se** recorded every 20 K in the 300 K (top) – 180 K (bottom) temperature range (600 MHz, dichloromethane-d_2).

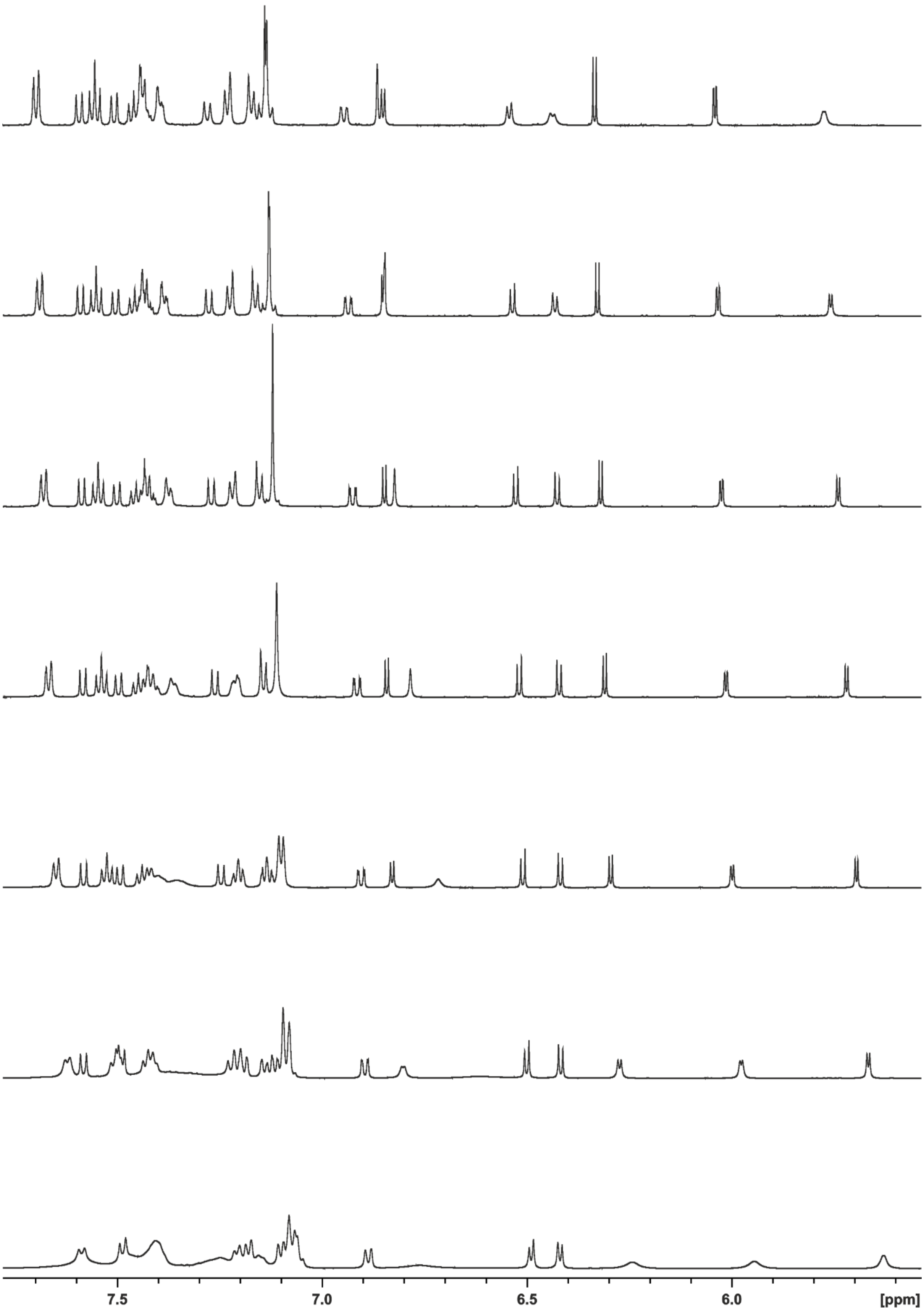

Figure S 80. Part of the ^{1}H NMR spectra of **4-Se** recorded every 20 K in the 300 K (top) – 180 K (bottom) temperature range (600 MHz, dichloromethane-d_2).

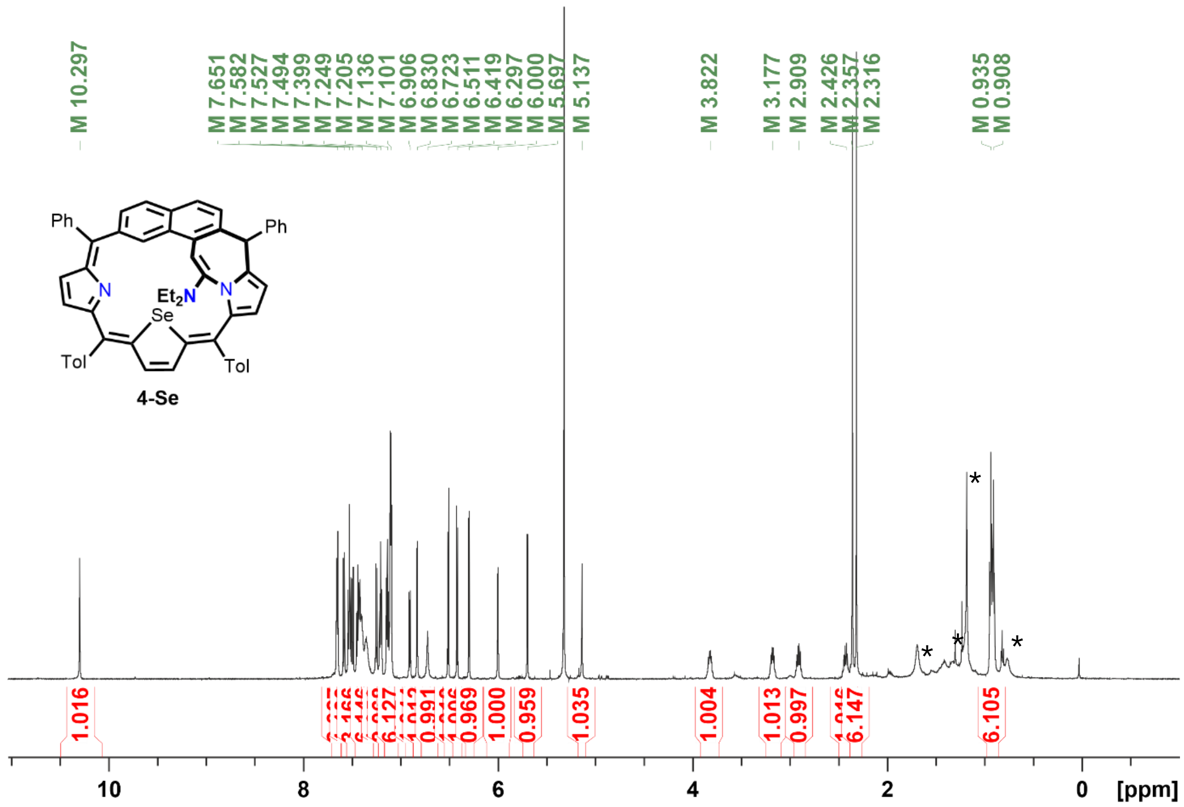

Figure S 81. The ^{1}H NMR spectrum of **4-Se** (600 MHz, dichloromethane-d_2, 220 K). Impurities were marked with asterisks.

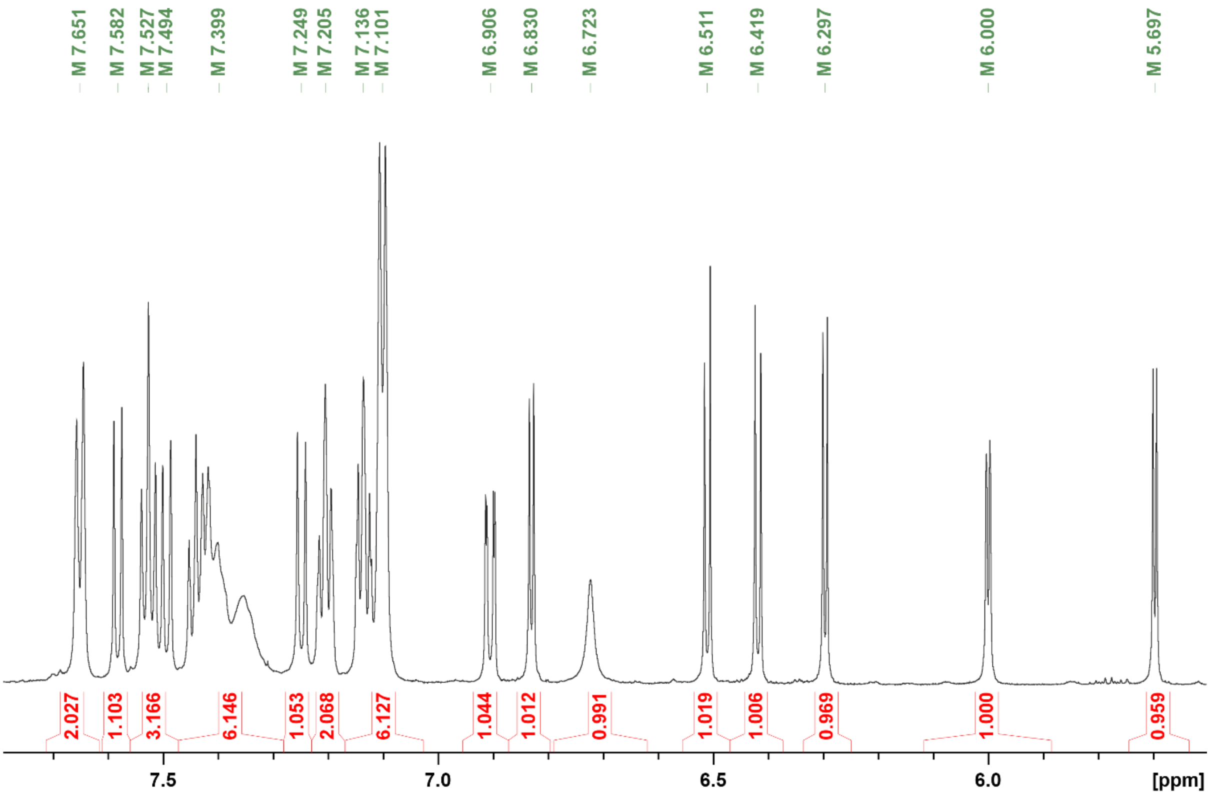

Figure S 82. Part of the ^{1}H NMR spectrum of **4-Se** (600 MHz, dichloromethane-d_2, 220 K).

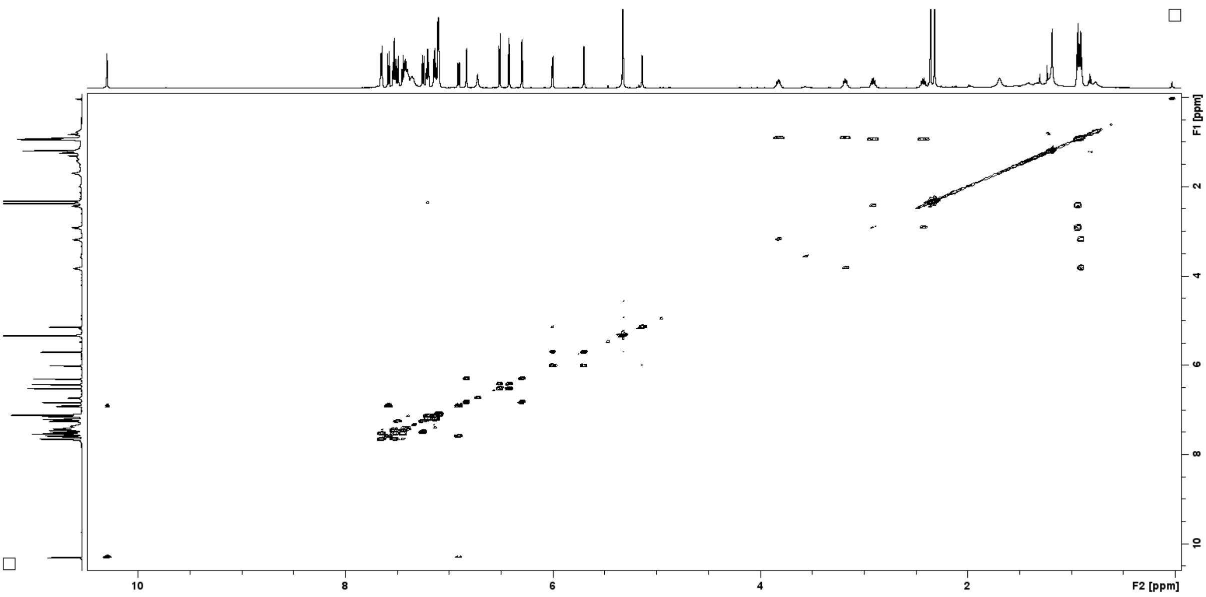

Figure S 83. The ^{1}H-^{1}H COSY NMR spectrum of **4-Se** (600 MHz, dichloromethane-d_2, 220 K).

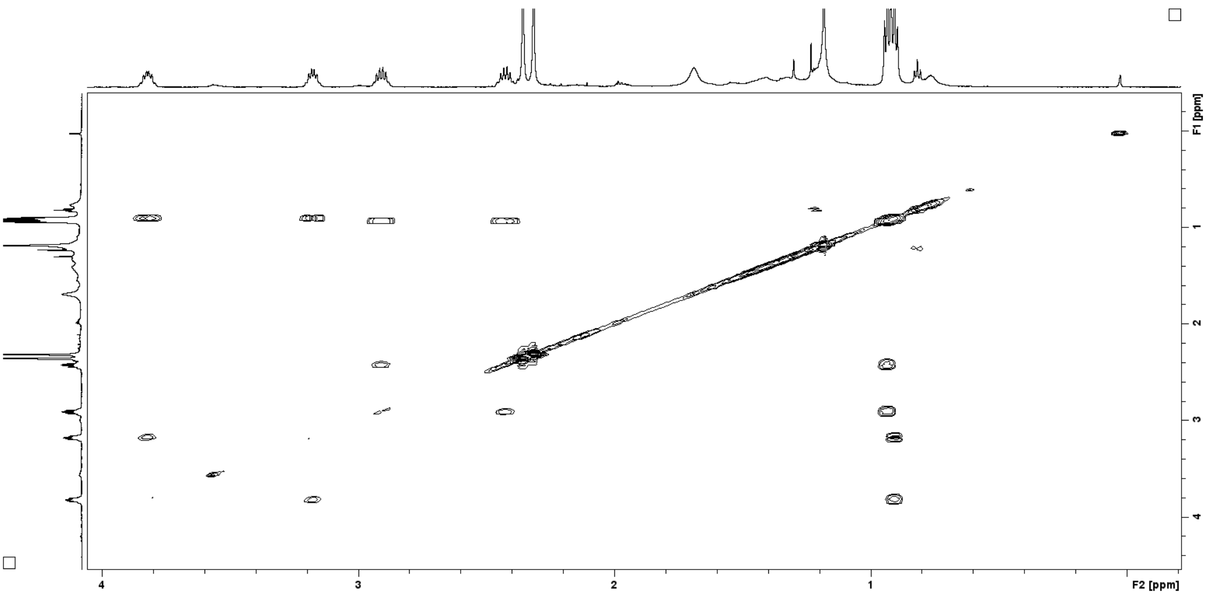

Figure S 84. Aliphatic part of the ^{1}H-^{1}H COSY NMR spectrum of **4-Se** (600 MHz, dichloromethane-d_2, 220 K).

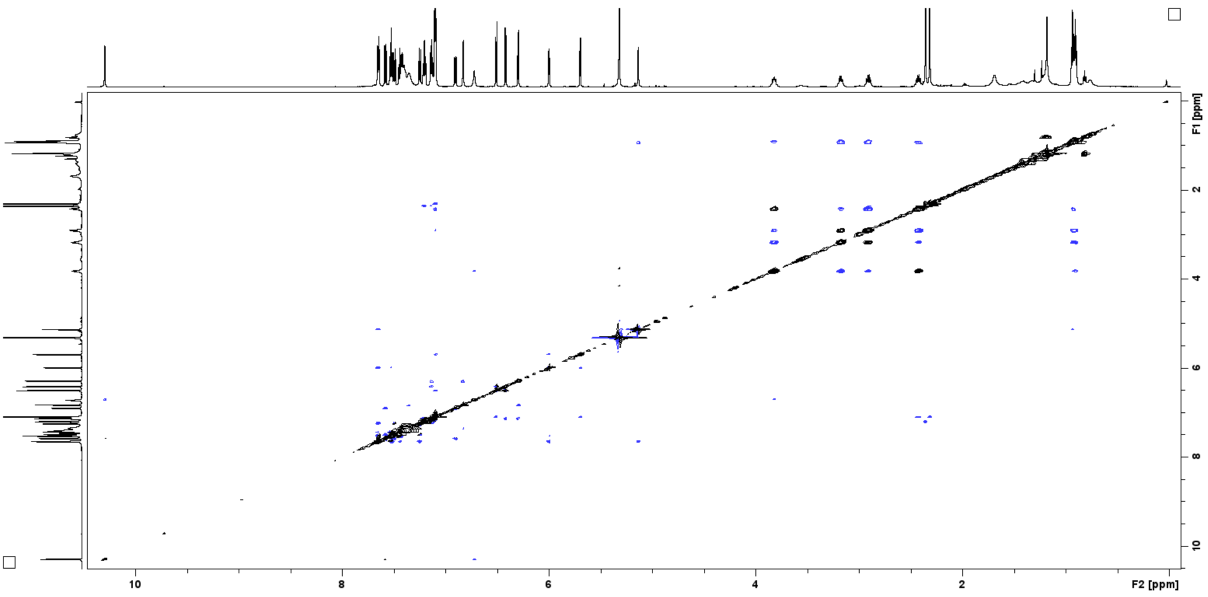

Figure S 85. The ^{1}H-^{1}H ROESY NMR spectrum of **4-Se** (600 MHz, dichloromethane-*d*$_2$, 220 K).

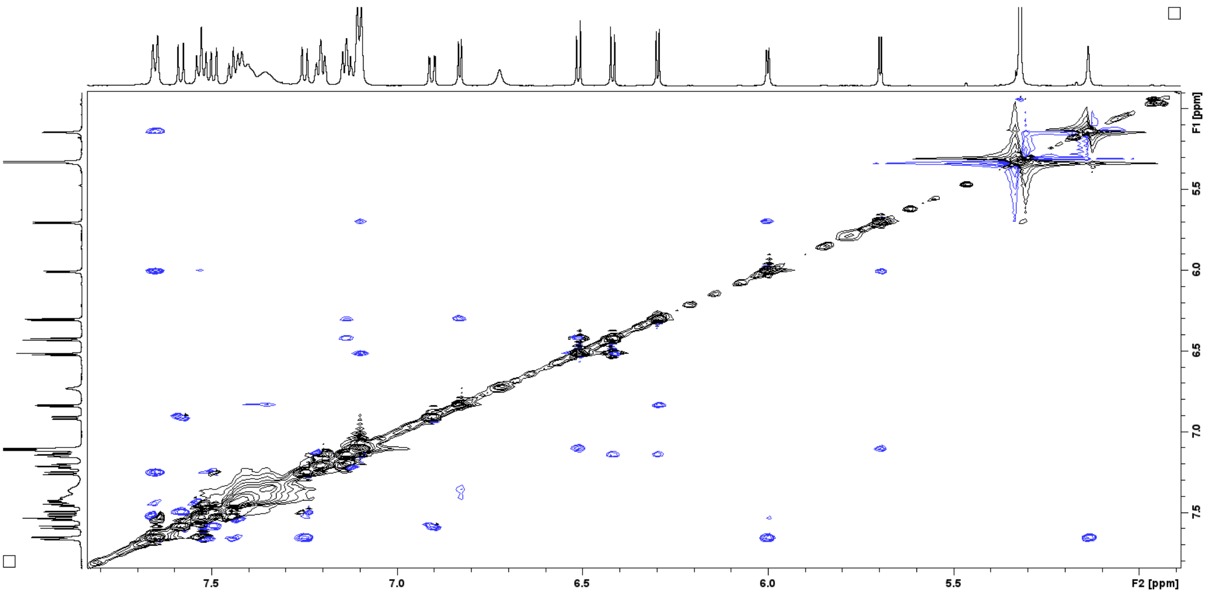

Figure S 86. Part of the ^{1}H-^{1}H ROESY NMR spectrum of **4-Se** (600 MHz, dichloromethane-*d*$_2$, 220 K).

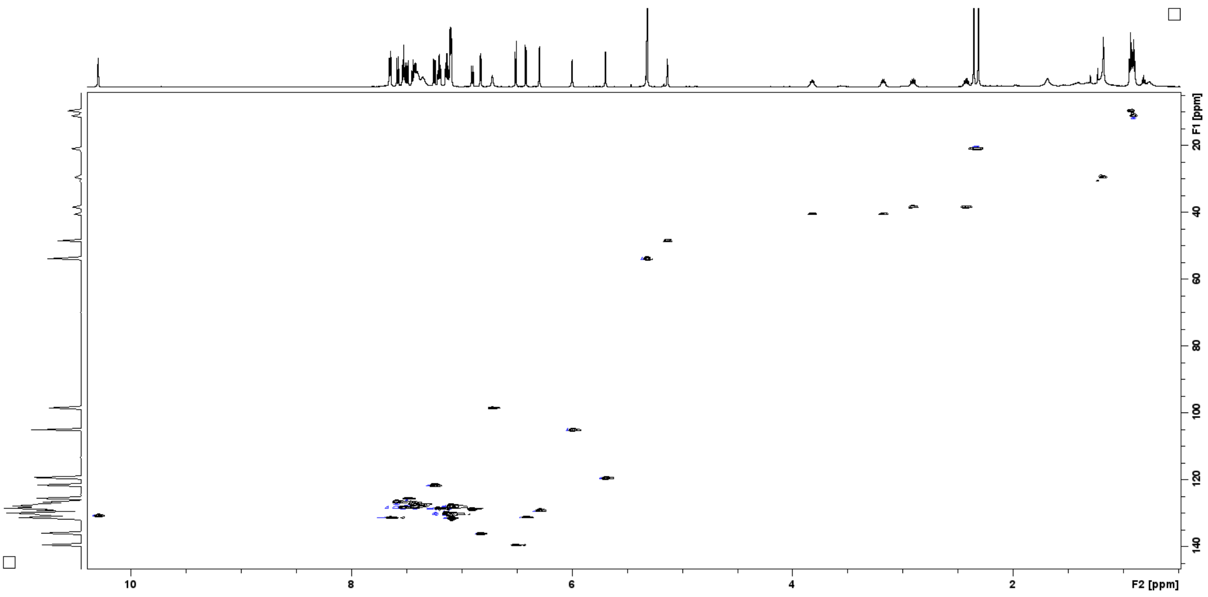

Figure S 87. The ^{1}H-^{13}C HSQC NMR spectrum of **4-Se** (600 MHz, dichloromethane-d_2, 220 K).

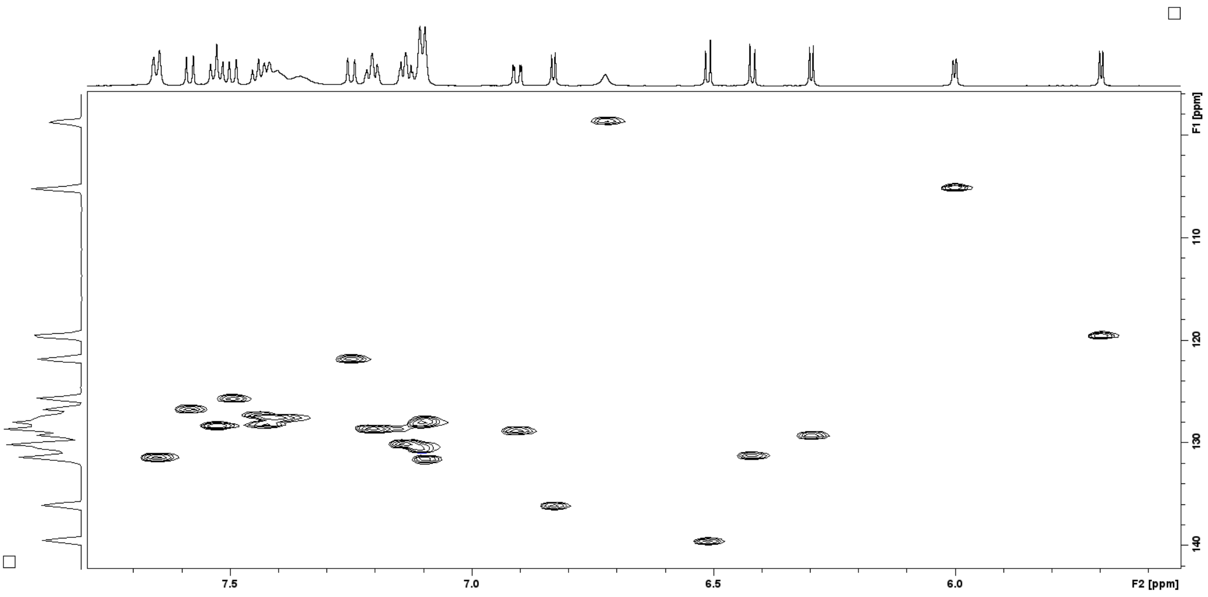

Figure S 88. Part of the ^{1}H-^{13}C HSQC NMR spectrum of **4-Se** (600 MHz, dichloromethane-d_2, 220 K).

NMR spectra of macrocycle 4-S

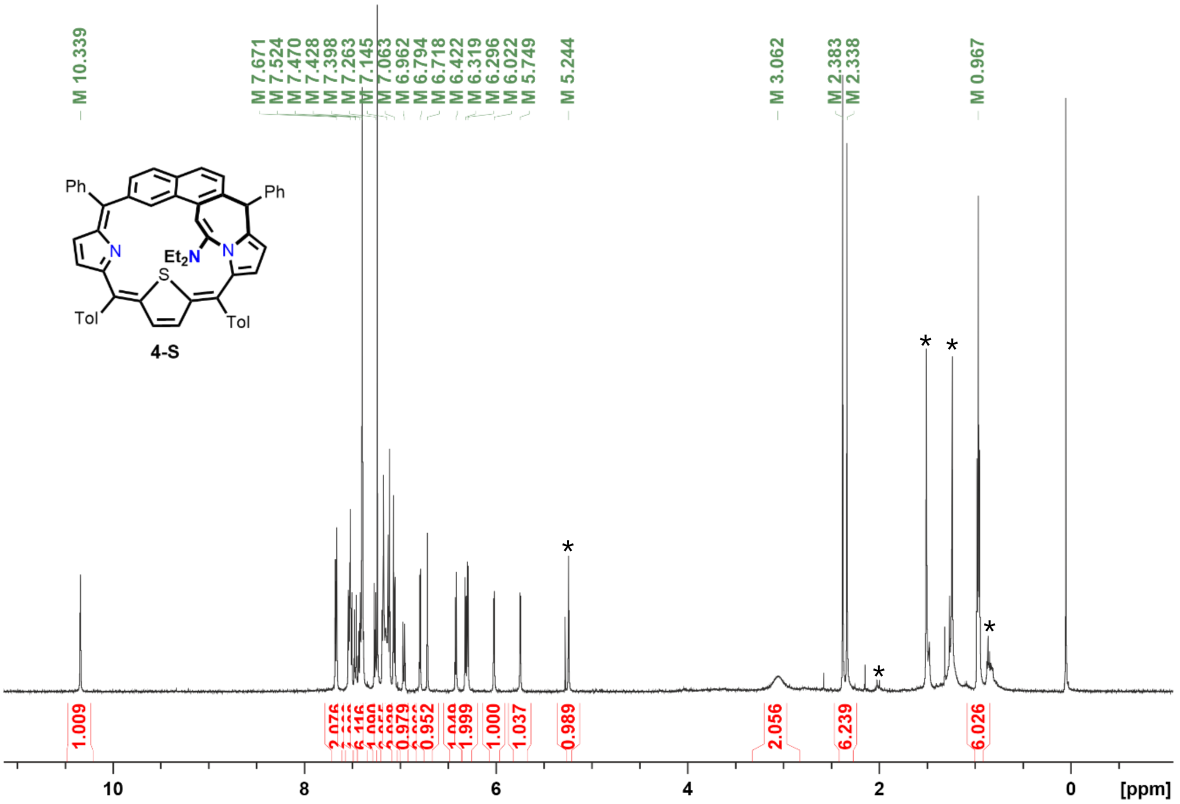

Figure S 89. The ^{1}H NMR spectrum of **4-S** (500 MHz, chloroform-*d*, 300 K). Impurities were marked with asterisks.

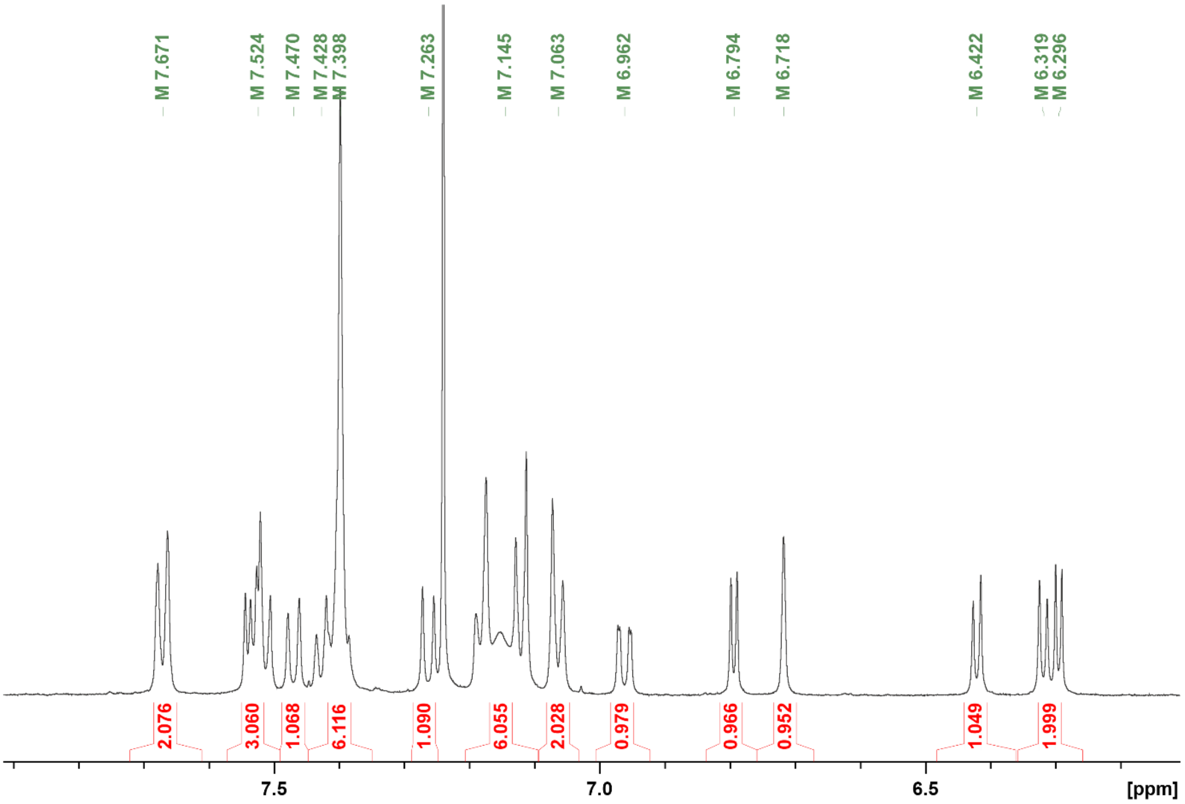

Figure S 90. Part of the ^{1}H NMR spectrum of **4-S** (500 MHz, chloroform-*d*, 300 K).

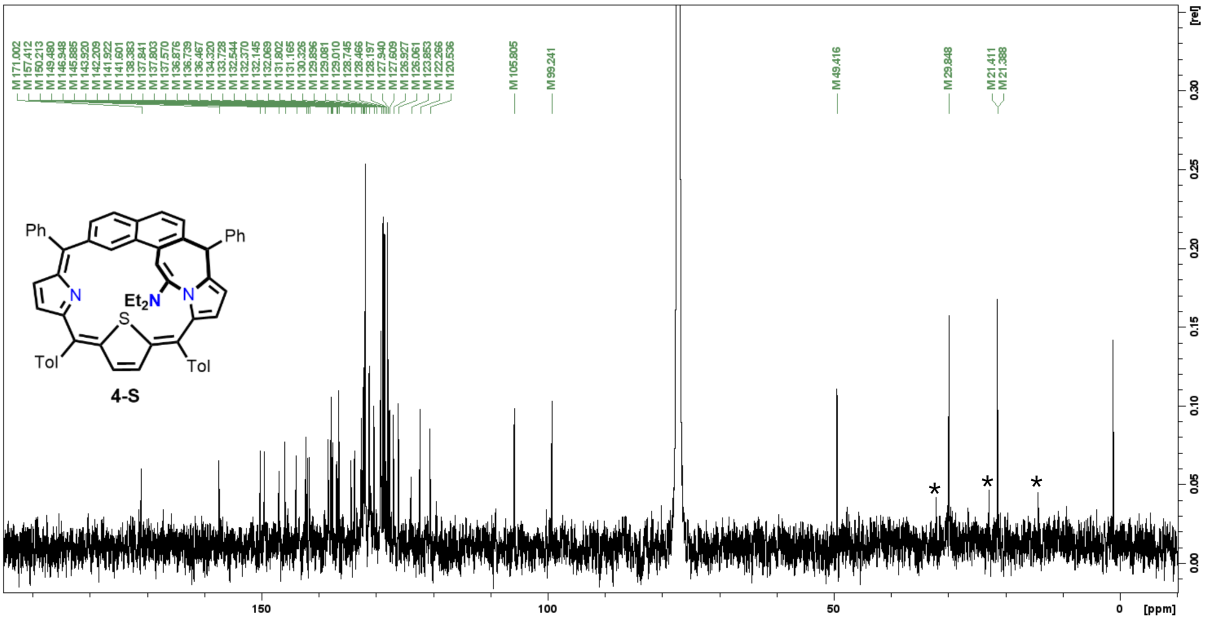

Figure S 91. The ^{13}C NMR spectrum of **4-S** (126 MHz, chloroform-*d*, 300 K). Impurities were marked with asterisks.

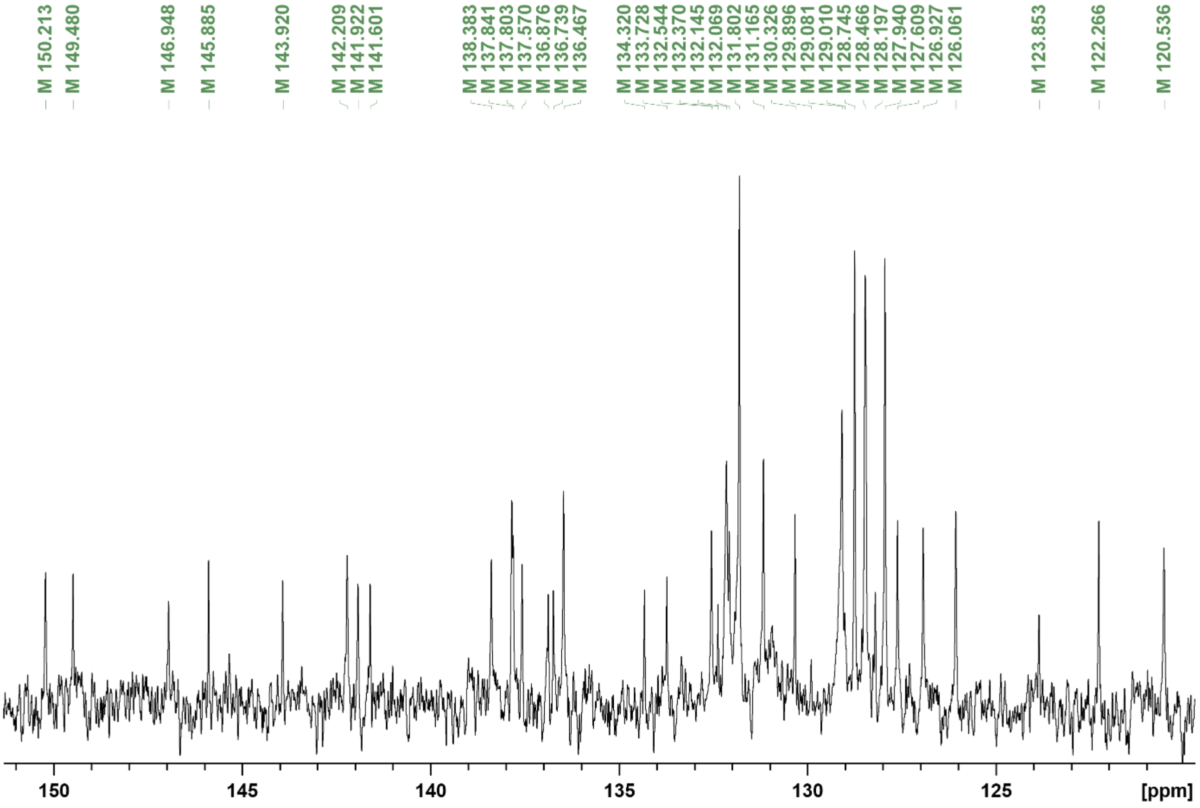

Figure S 92. Part of the ^{13}C NMR spectrum of **4-S** (126 MHz, chloroform-*d*, 300 K).

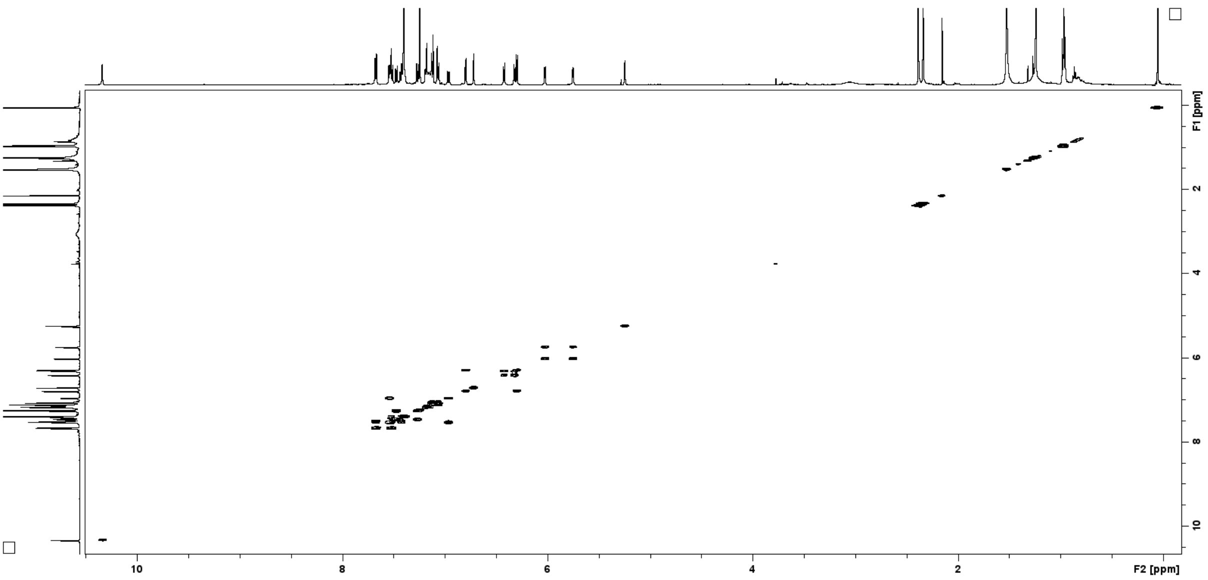

Figure S 93. The ^{1}H-^{1}H COSY NMR spectrum of **4-S** (500 MHz, chloroform-*d*, 300 K).

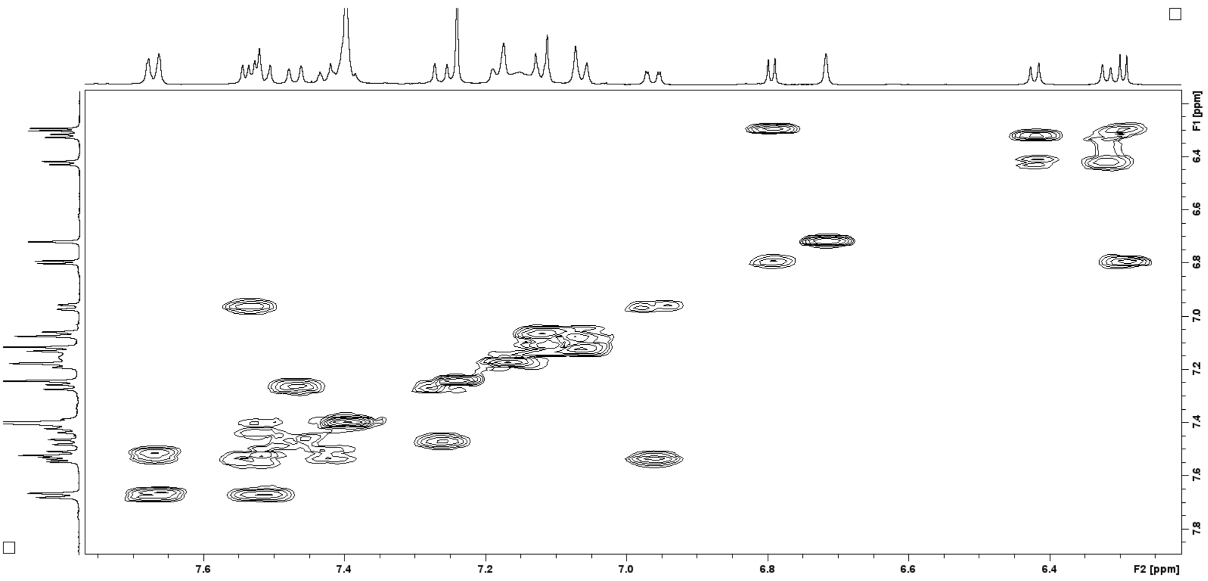

Figure S 94. Part of the ^{1}H-^{1}H COSY NMR spectrum of **4-S** (500 MHz, chloroform-*d*, 300 K).

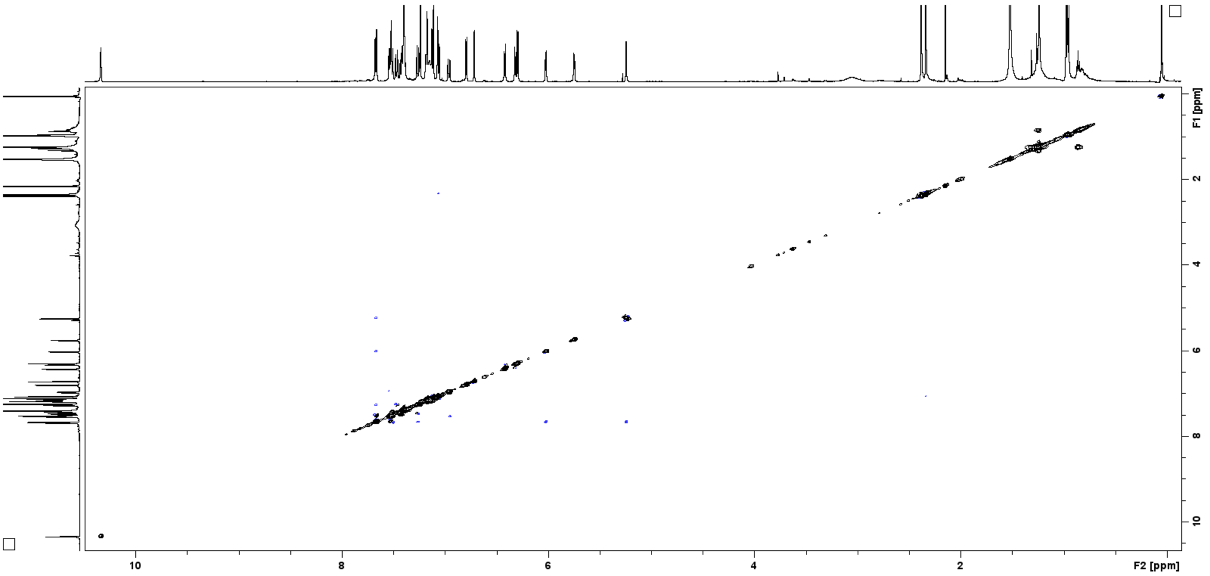

Figure S 95. The ^{1}H-^{1}H ROESY NMR spectrum of **4-S** (500 MHz, chloroform-*d*, 300 K).

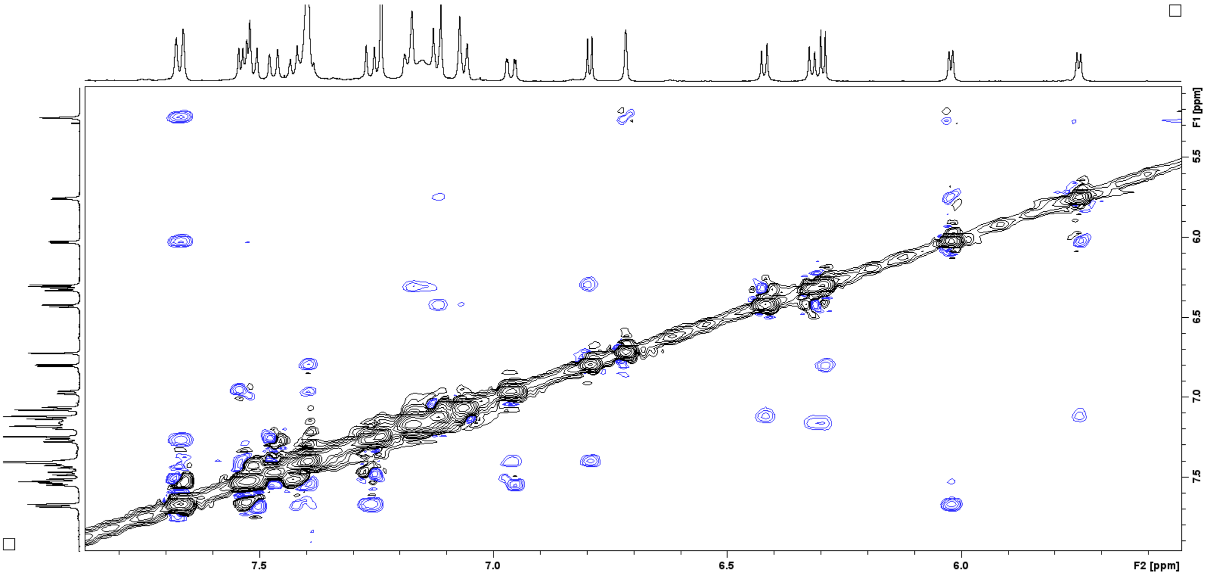

Figure S 96. Part of the ^{1}H-^{1}H ROESY NMR spectrum of **4-S** (500 MHz, chloroform-*d*, 300 K).

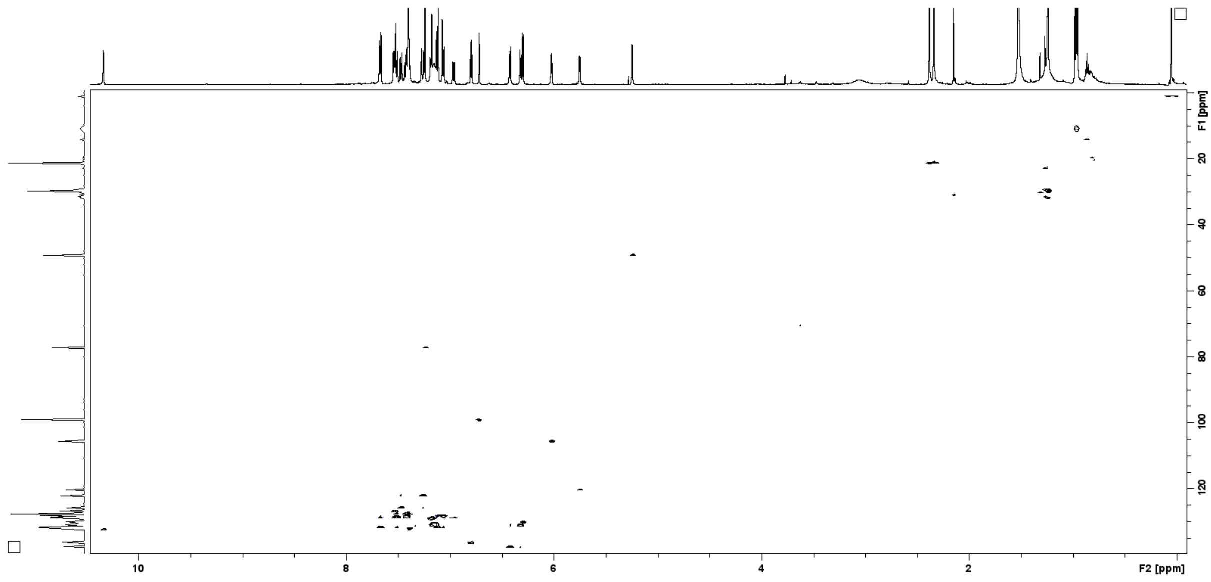

Figure S 97. The ^{1}H-^{13}C HSQC NMR spectrum of **4-S** (500 MHz, chloroform-*d*, 300 K).

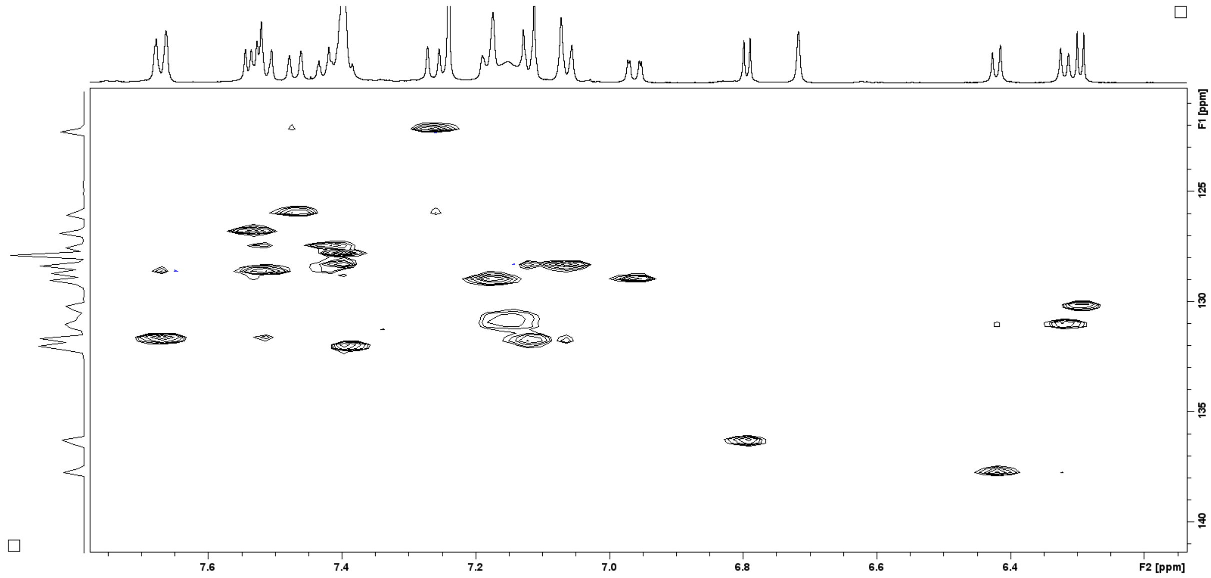

Figure S 98. Part of the ^{1}H-^{13}C HSQC NMR spectrum of **4-S** (500 MHz, chloroform-*d*, 300 K).

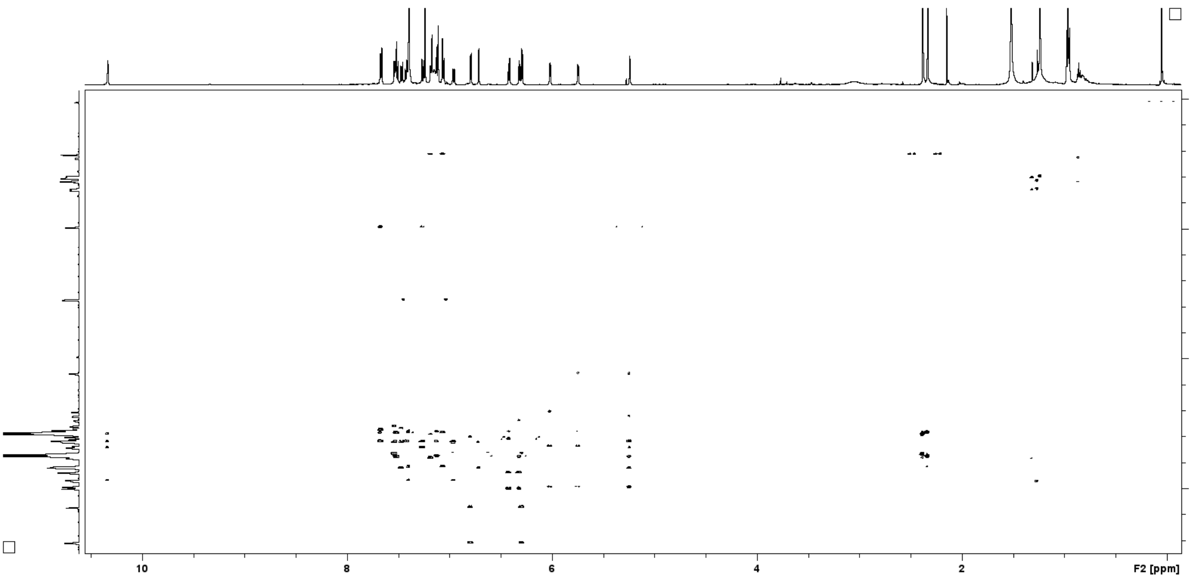

Figure S 99. The ^{1}H-^{13}C HMBC NMR spectrum of **4-S** (500 MHz, chloroform-*d*, 300 K).

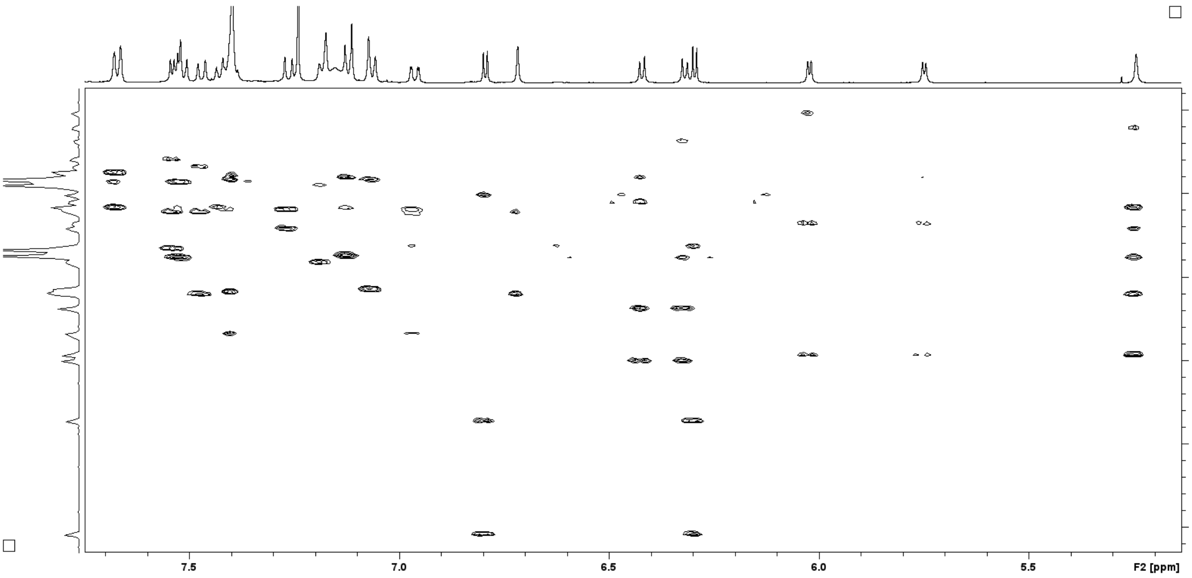

Figure S 100. Part of the ^{1}H-^{13}C HMBC NMR spectrum of **4-S** (500 MHz, chloroform-*d*, 300 K).

NMR spectra of crude reaction mixtures

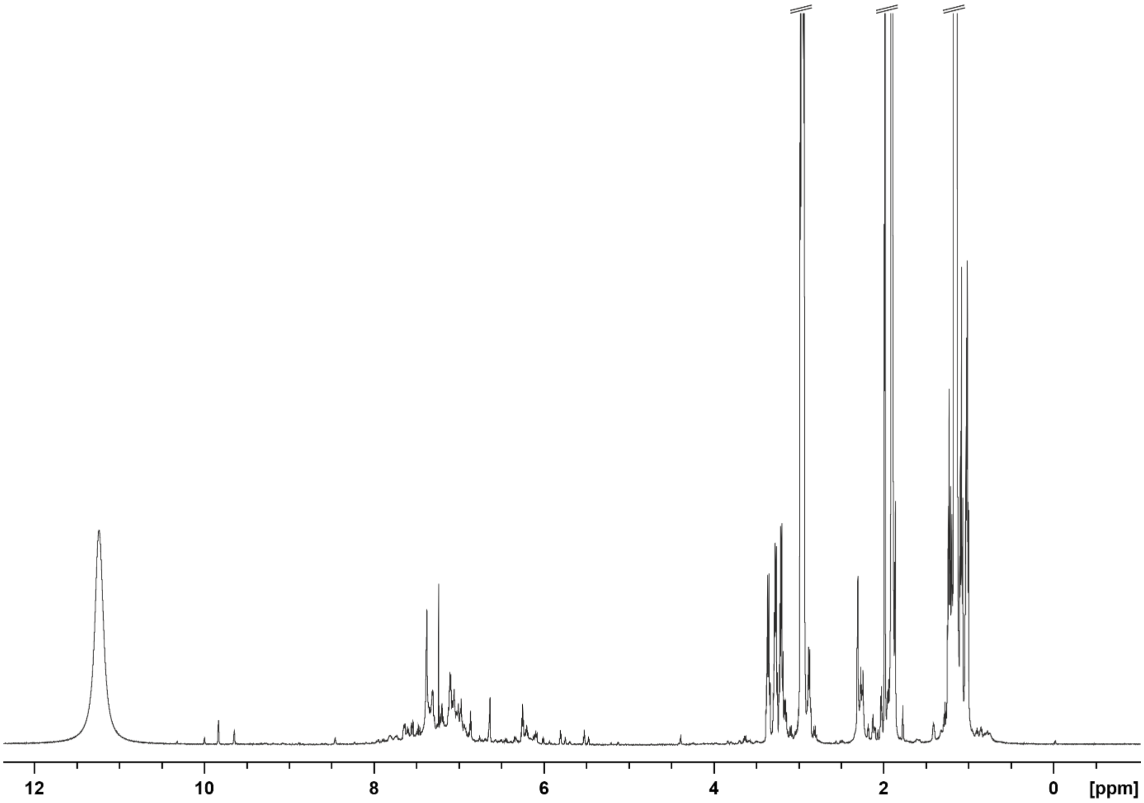

Figure S 101. The ^{1}H NMR spectrum of the crude mixture after the reaction of **1-Se** with commercially available triethylamine (500 MHz, chloroform-*d*, 300 K).

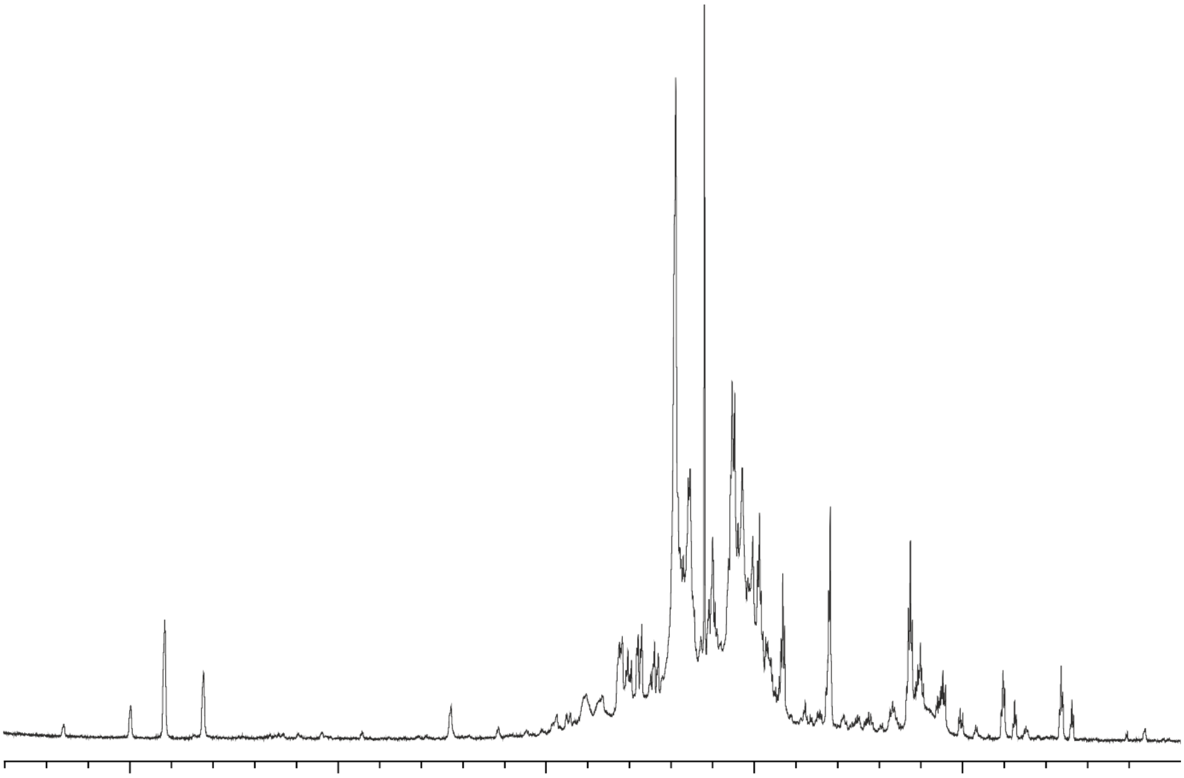

Figure S 102. Part of the ^{1}H NMR spectrum of the crude mixture after the reaction of **1-Se** with commercially available triethylamine (500 MHz, chloroform-*d*, 300 K).

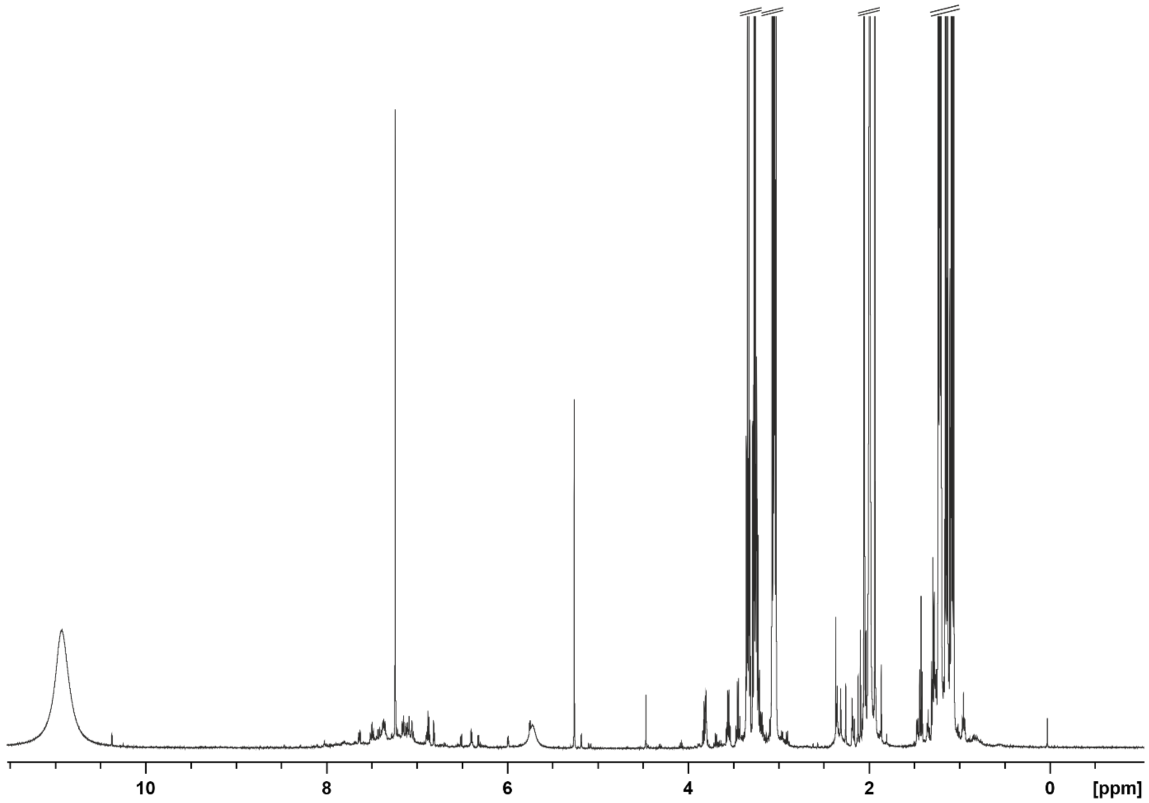

Figure S 103. The ^{1}H NMR spectrum of the crude mixture after the reaction of **1-Se** with purified triethylamine (500 MHz, chloroform-*d*, 300 K).

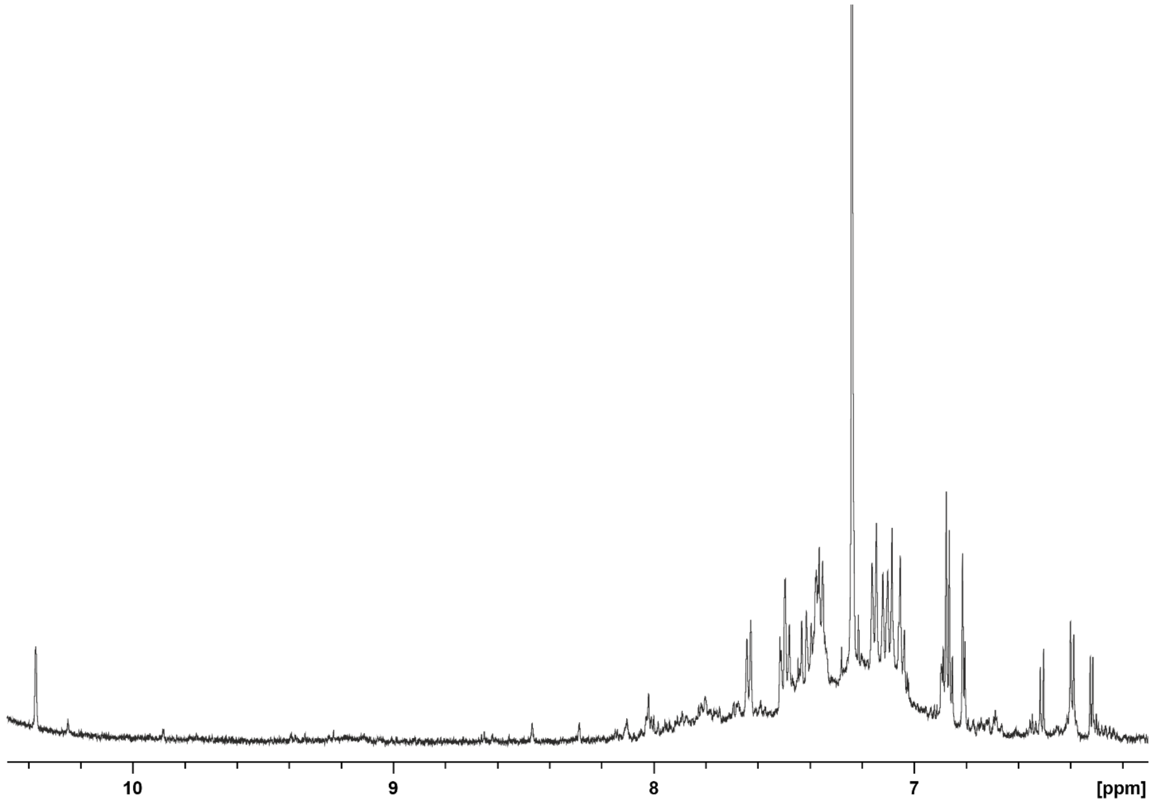

Figure S 104. Part of the ^{1}H NMR spectrum of the crude mixture after the reaction of **1-Se** with purified triethylamine (500 MHz, chloroform-*d*, 300 K).

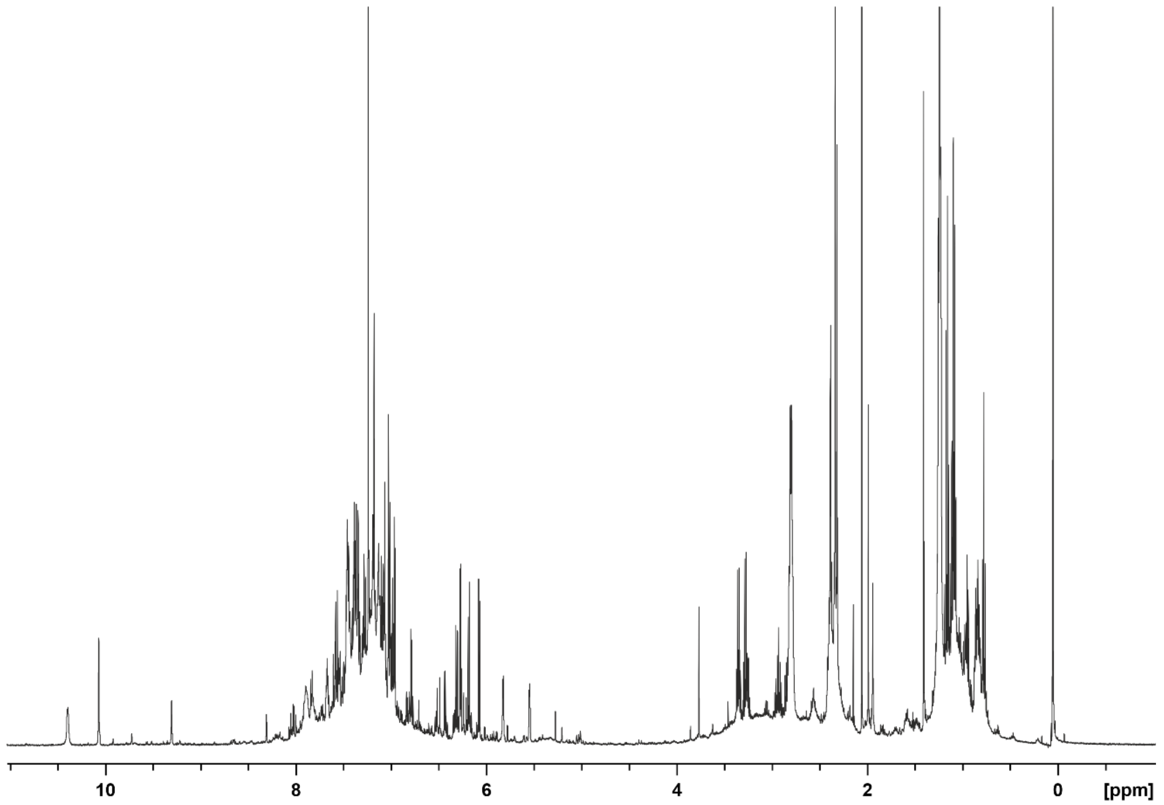

Figure S 105. The ^{1}H NMR spectrum of the crude mixture after the reaction of **1-Se** with commercially available diethylamine (500 MHz, chloroform-*d*, 300 K).

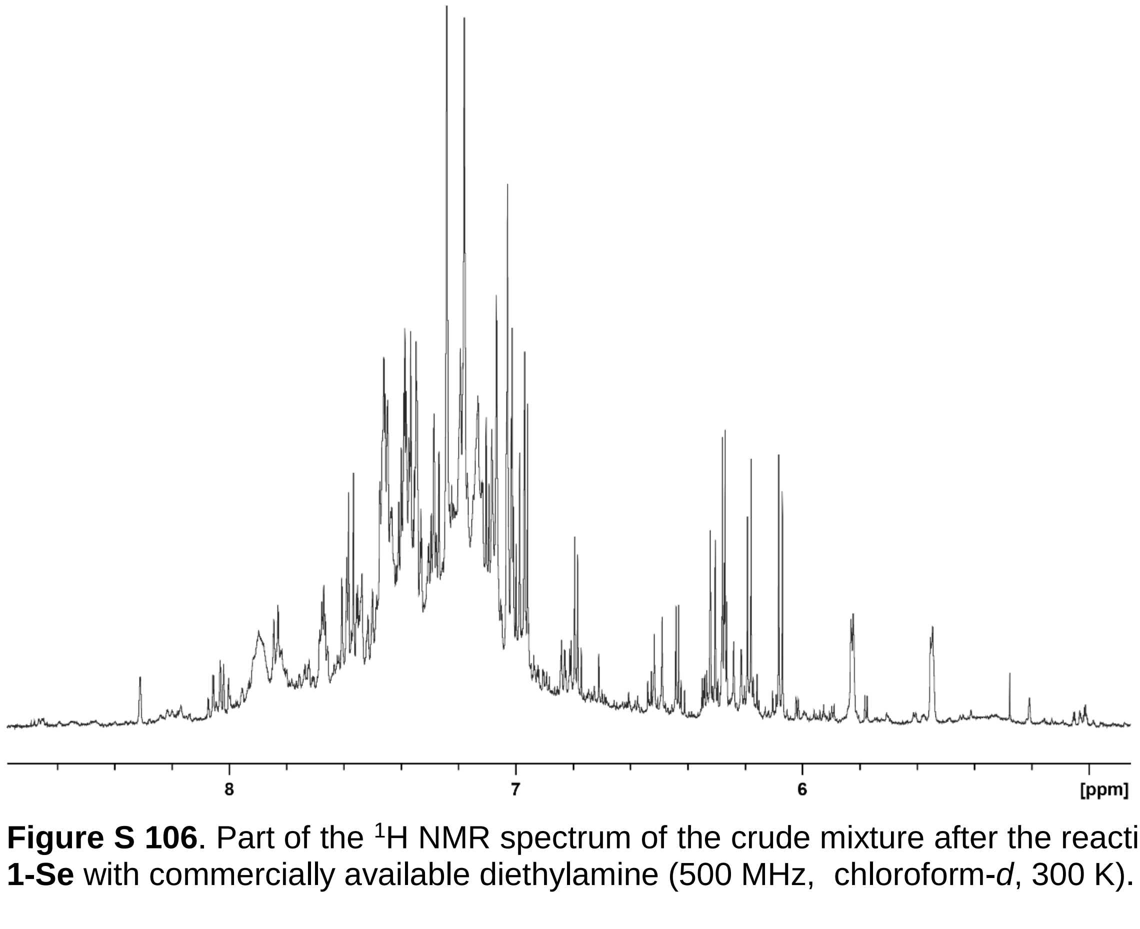

Figure S 106. Part of the ^{1}H NMR spectrum of the crude mixture after the reaction of **1-Se** with commercially available diethylamine (500 MHz, chloroform-*d*, 300 K).

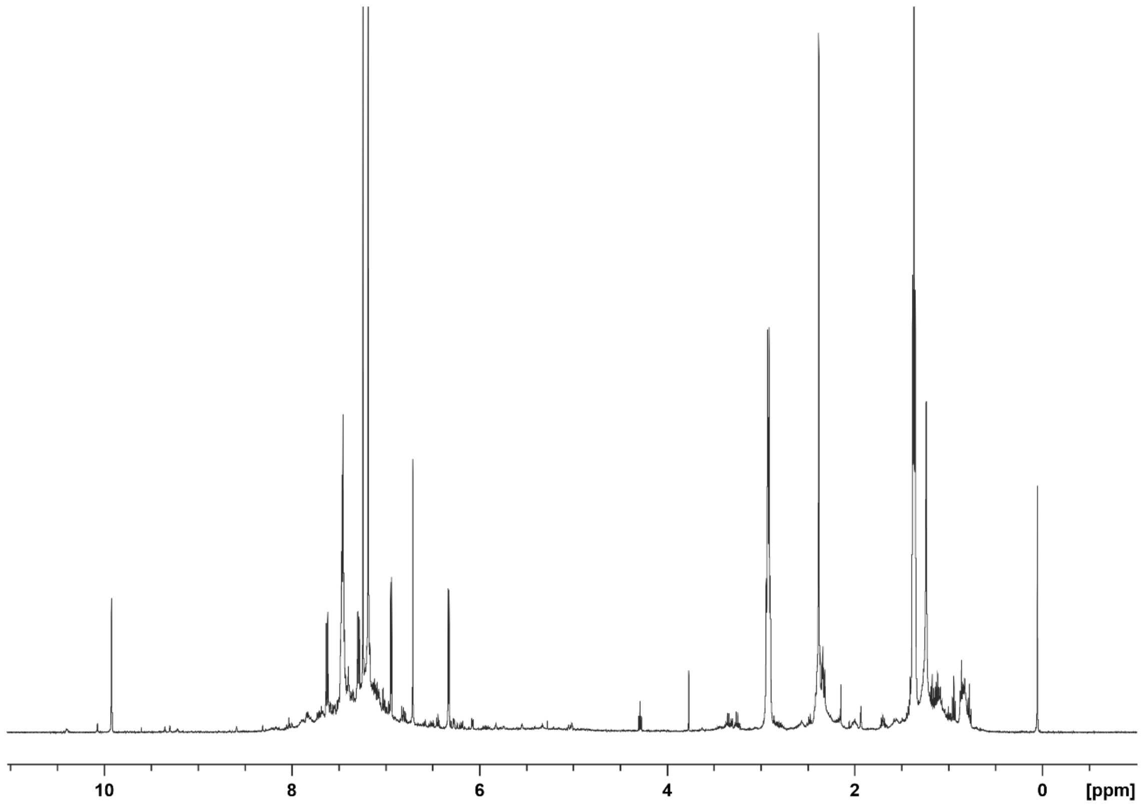

Figure S 107. The ^{1}H NMR spectrum of the crude mixture after the reaction of **1-Se** with purified diethylamine (500 MHz, chloroform-*d*, 300 K).

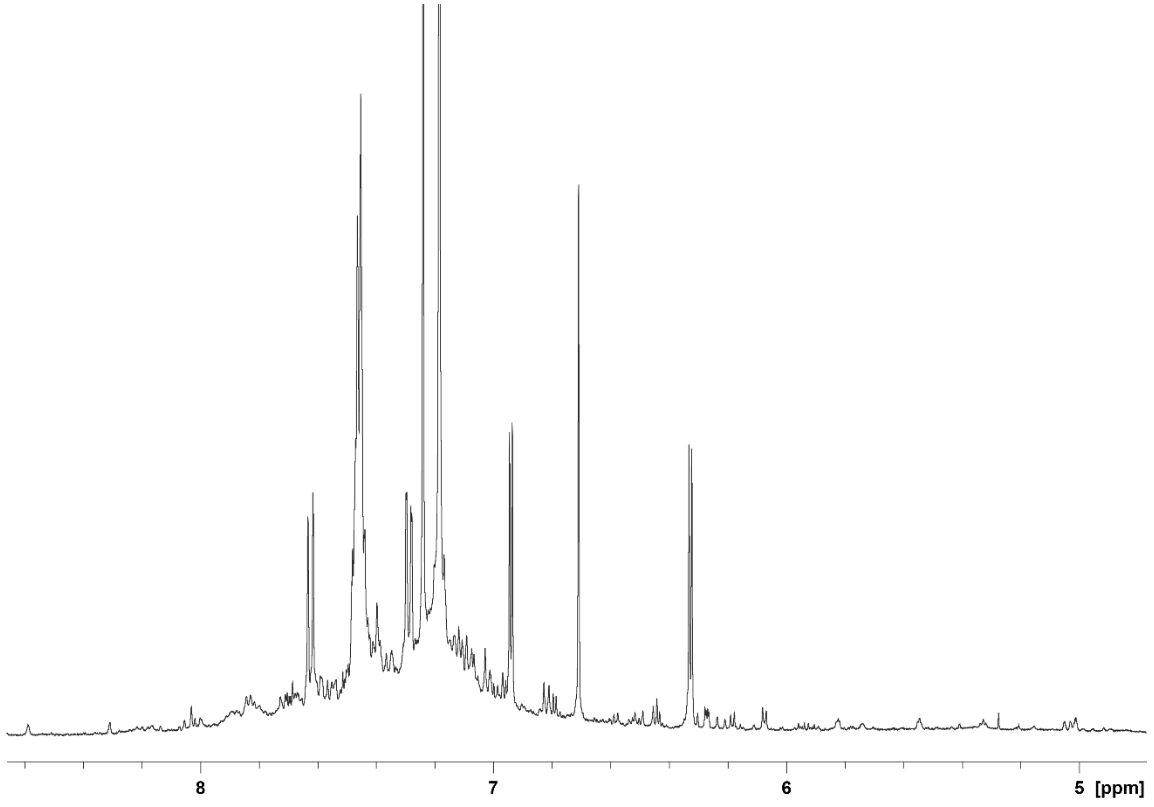

Figure S 108. Part of the ^{1}H NMR spectrum of the crude mixture after the reaction of **1-Se** with purified diethylamine (500 MHz, chloroform-*d*, 300 K).

High-resolution mass spectra

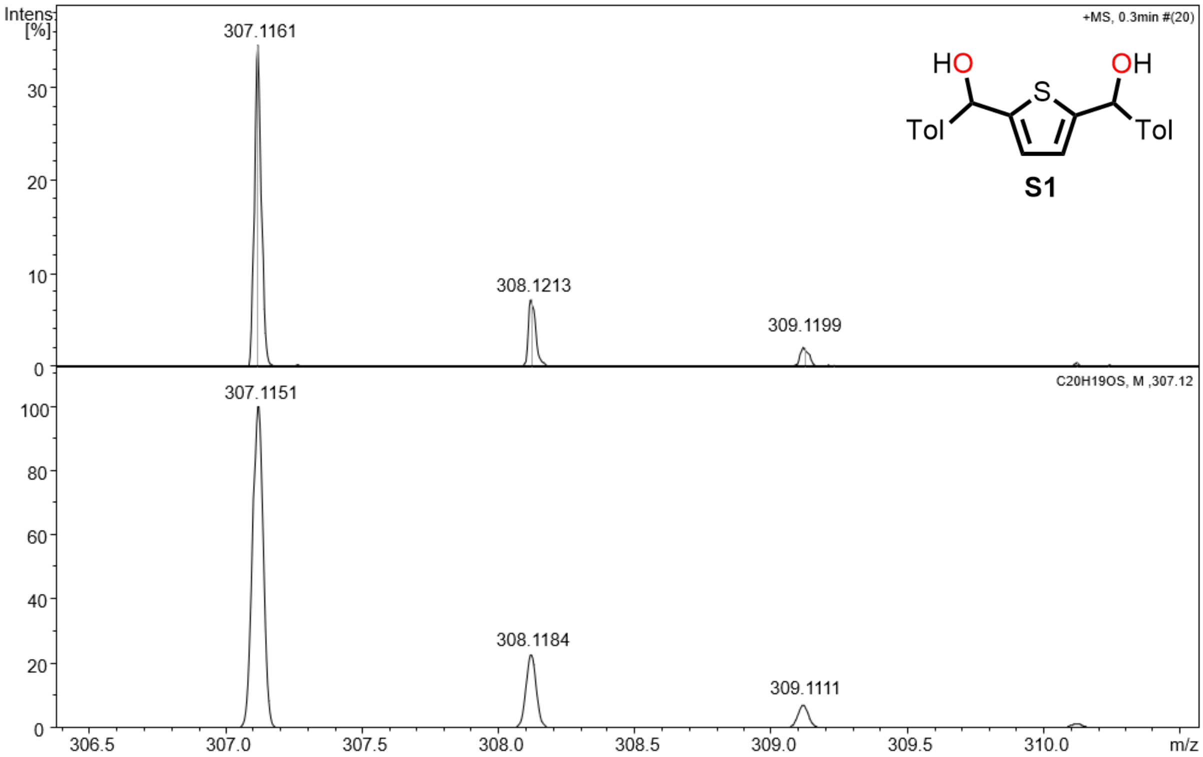

Figure S 109. The HR-MS spectrum of **S1** (ESI, TOF, $[M-OH]^+$) (top) and simulated pattern (bottom).

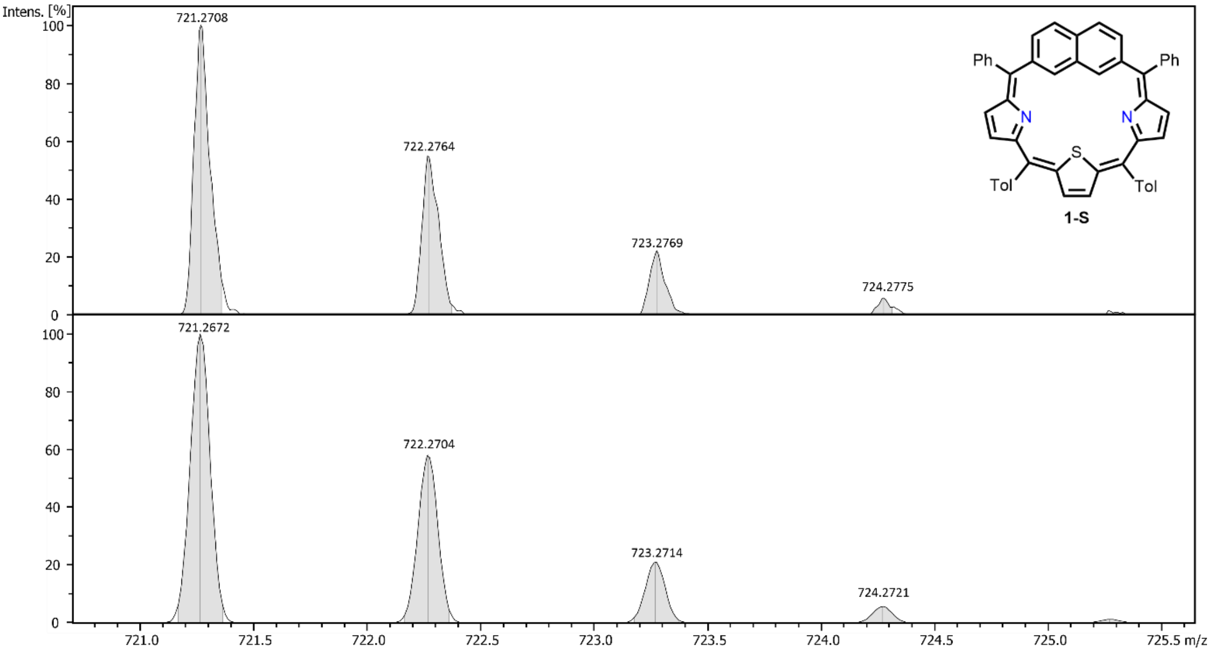

Figure S 110. The HR-MS spectrum of **1-S** (ESI, TOF, $[M+H]^+$) (top) and simulated pattern (bottom).

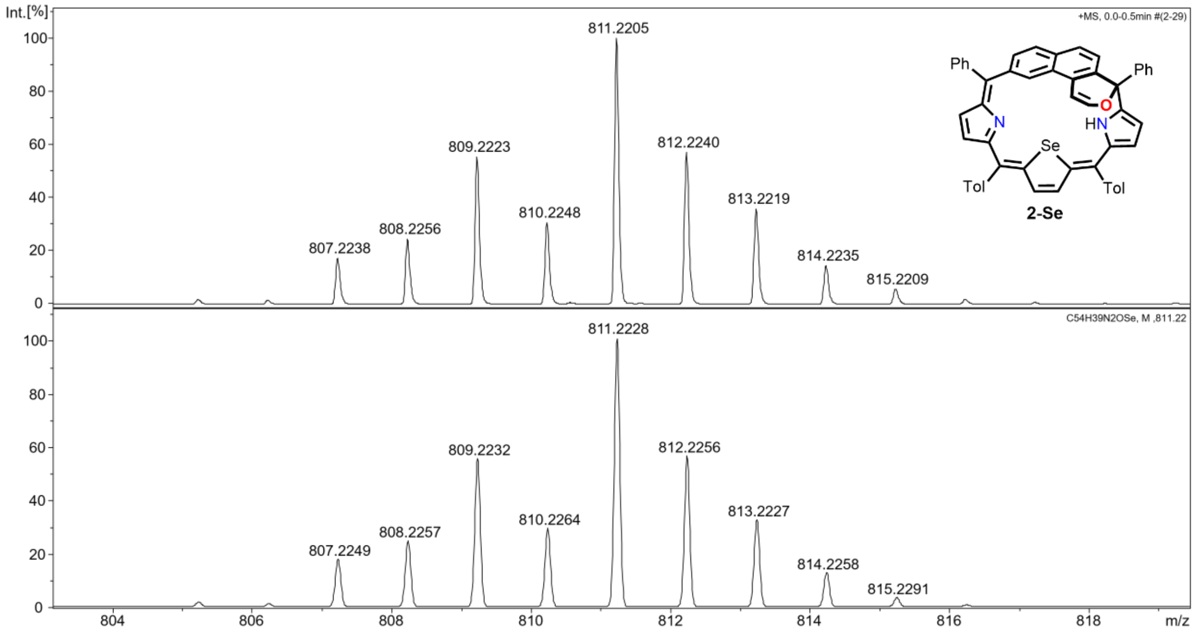

Figure S 111. The HR-MS spectrum of **2-Se** (ESI, TOF, $[M+H]^+$) (top) and simulated pattern (bottom).

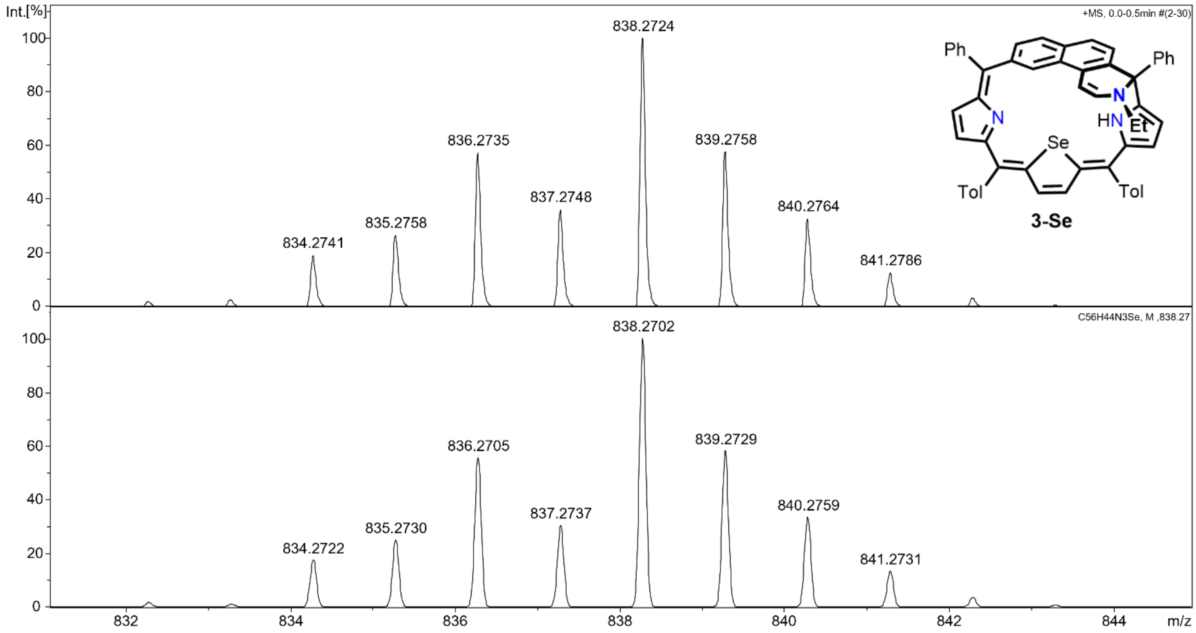

Figure S 112. The HR-MS spectrum of **3-Se** (ESI, TOF, $[M+H]^+$) (top) and simulated pattern (bottom).

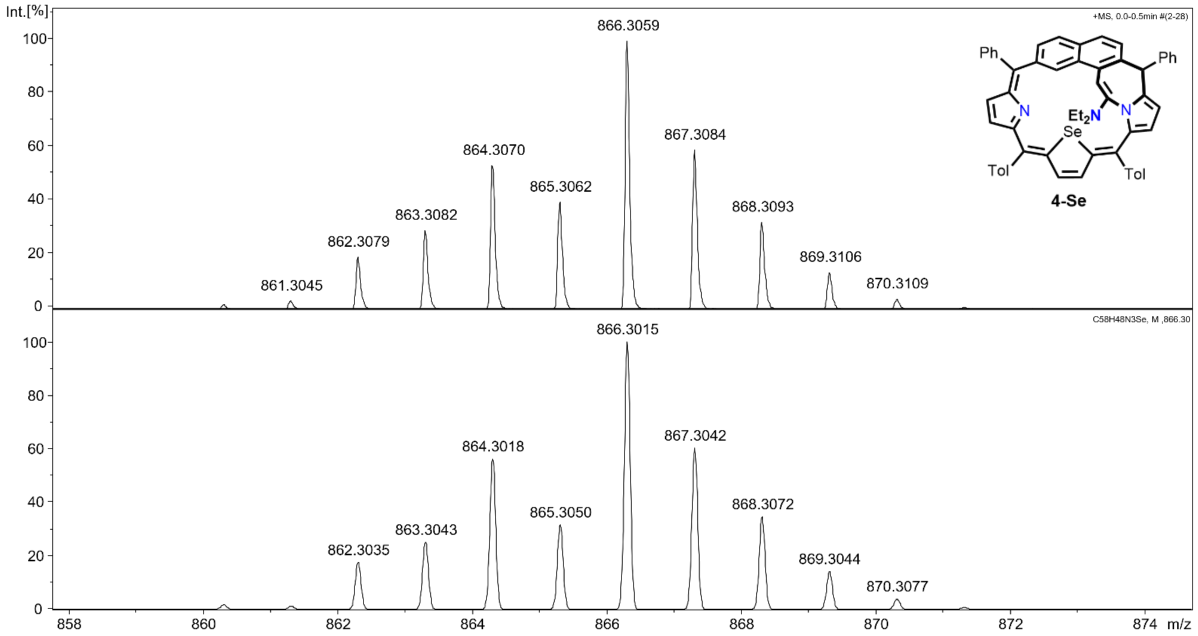

Figure S 113. The HR-MS spectrum of **4-Se** (ESI, TOF, $[M+H]^+$) (top) and simulated pattern (bottom).

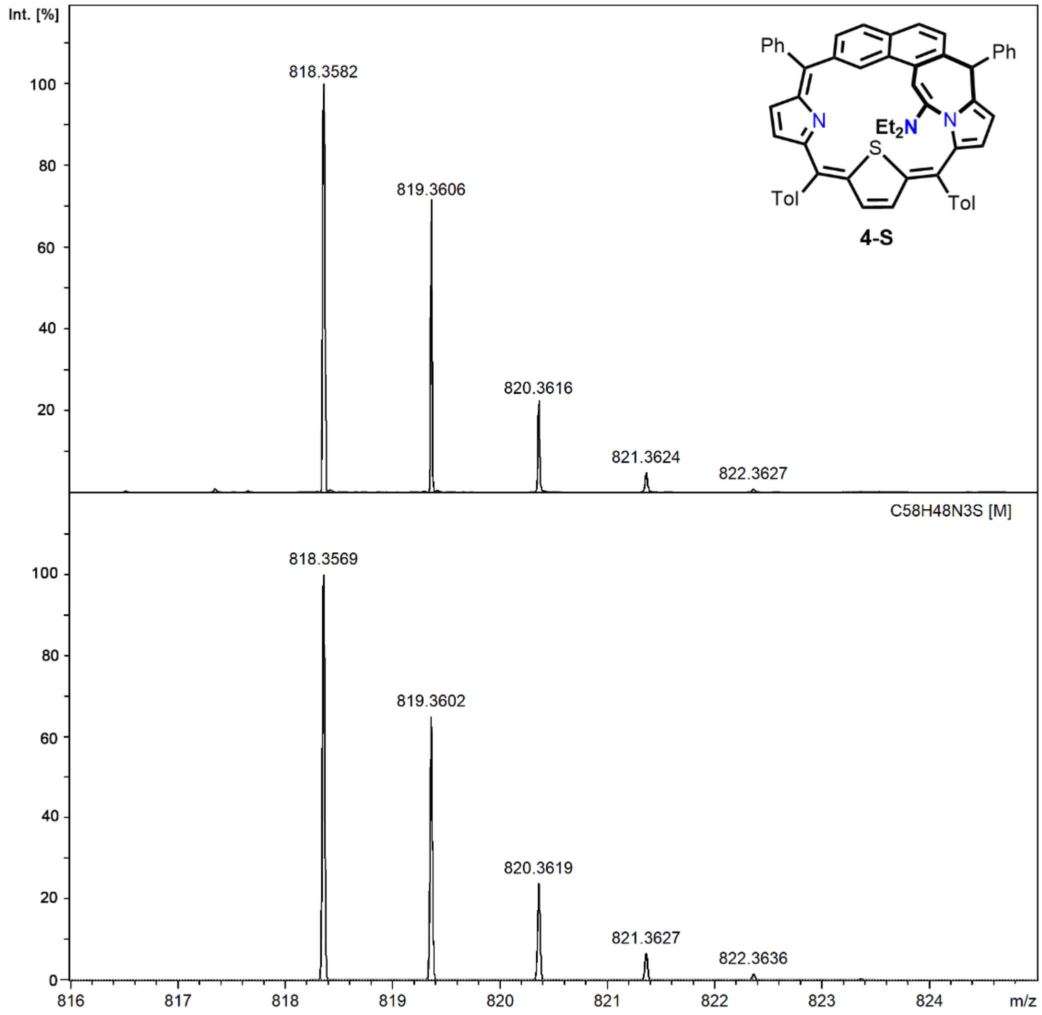

Figure S 114. The HR-MS spectrum of **4-S** (ESI, TOF, $[M+H]^+$) (top) and simulated pattern (bottom).

UV-Vis absorption spectra

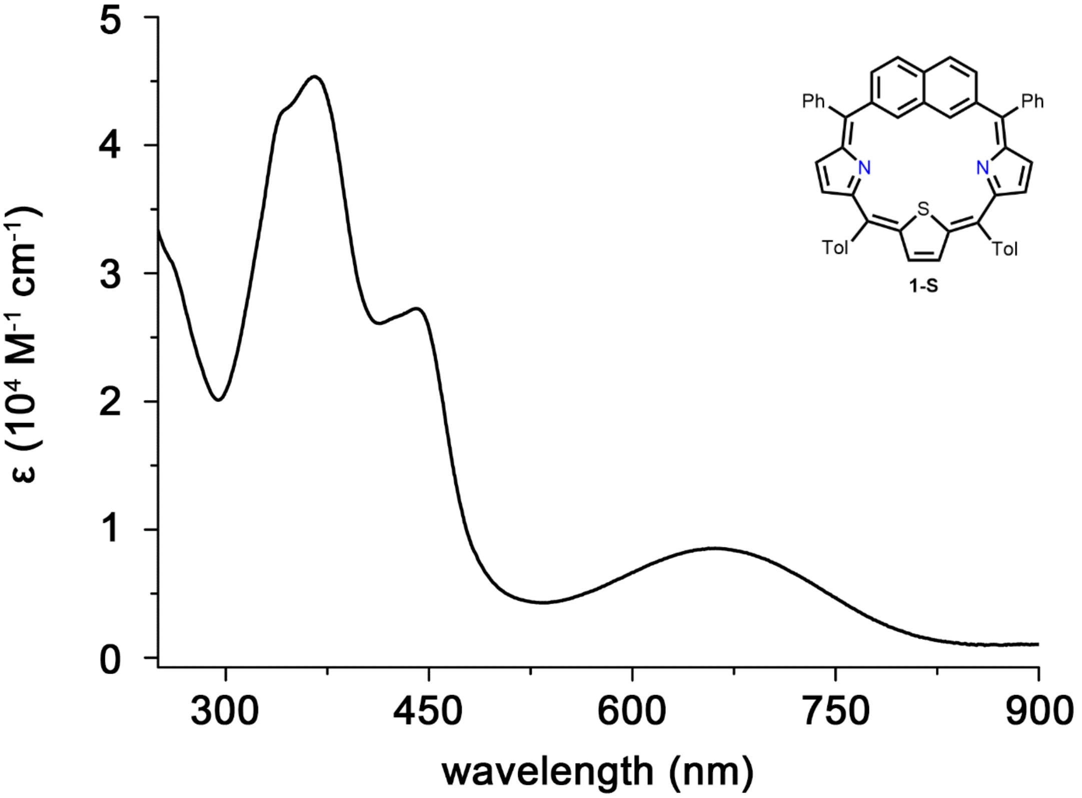

Figure S 115. The UV-Vis absorption spectrum of **1-S** (dichloromethane, 298 K).

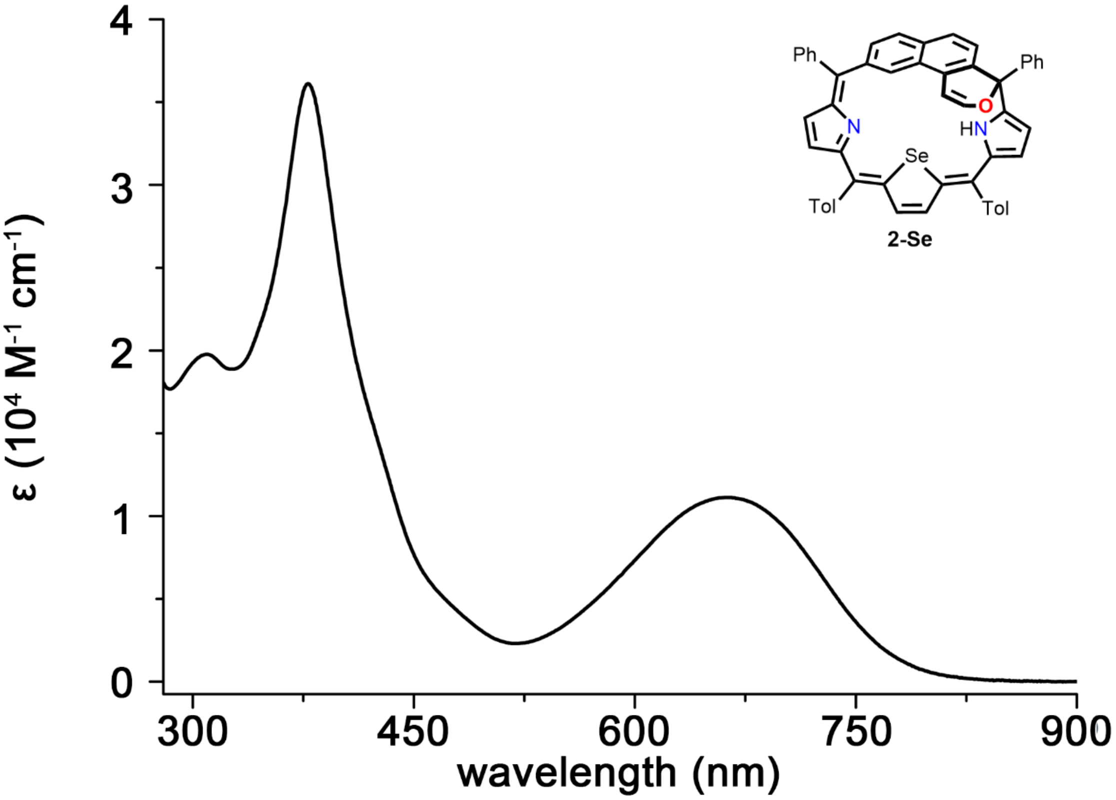

Figure S 116. The UV-Vis absorption spectrum of **2-Se** (chloroform, 298 K).

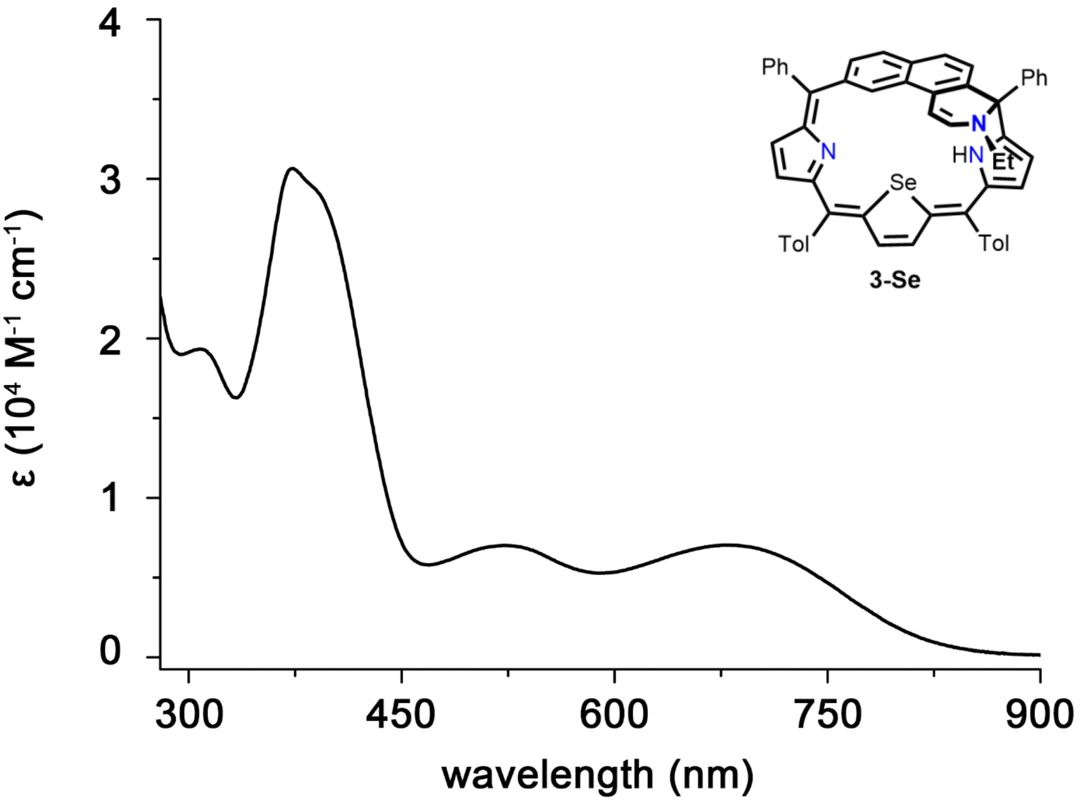

Figure S 117. The UV-Vis absorption spectrum of **3-Se** (dichloromethane, 298 K).

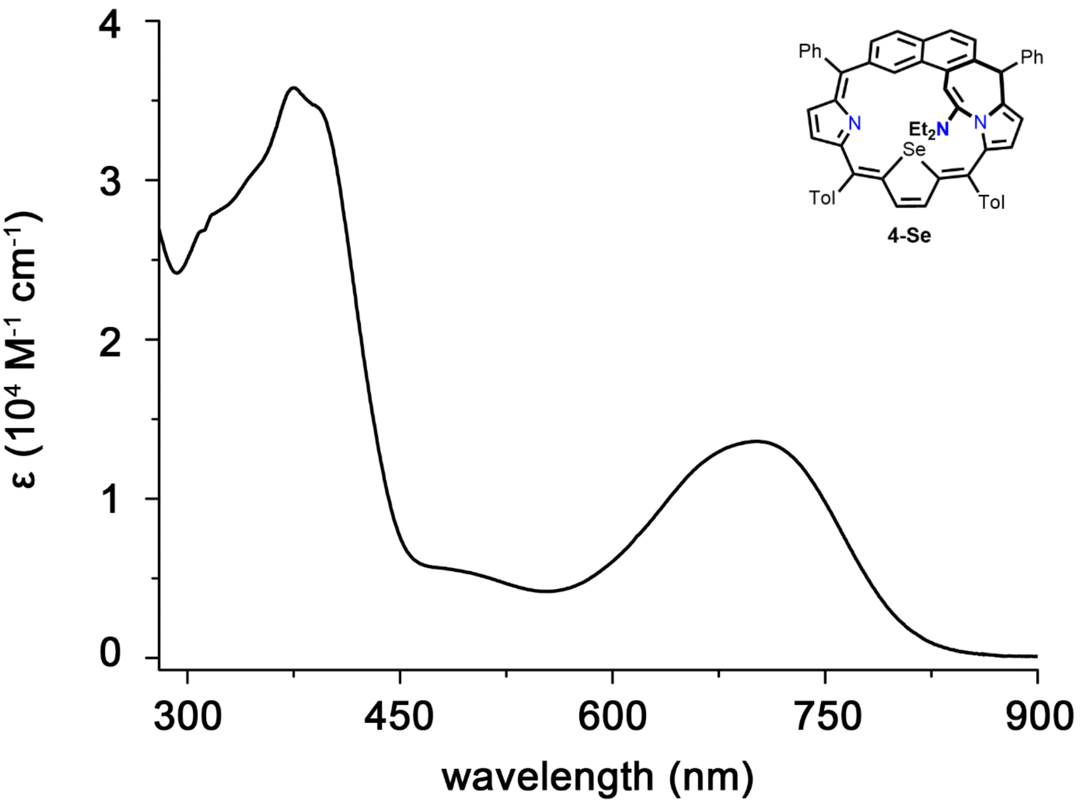

Figure S 118. The UV-Vis absorption spectrum of **4-Se** (chloroform, 298 K).

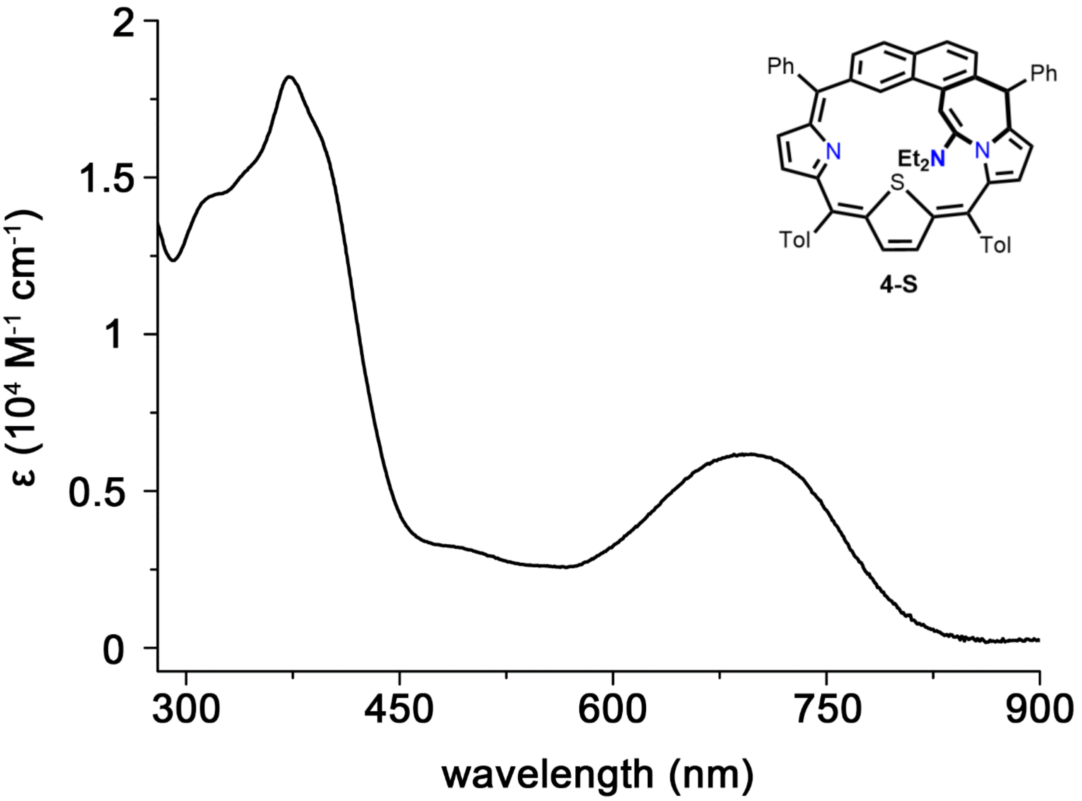

Figure S 119. The UV-Vis absorption spectrum of **4-S** (dichloromethane, 298 K).

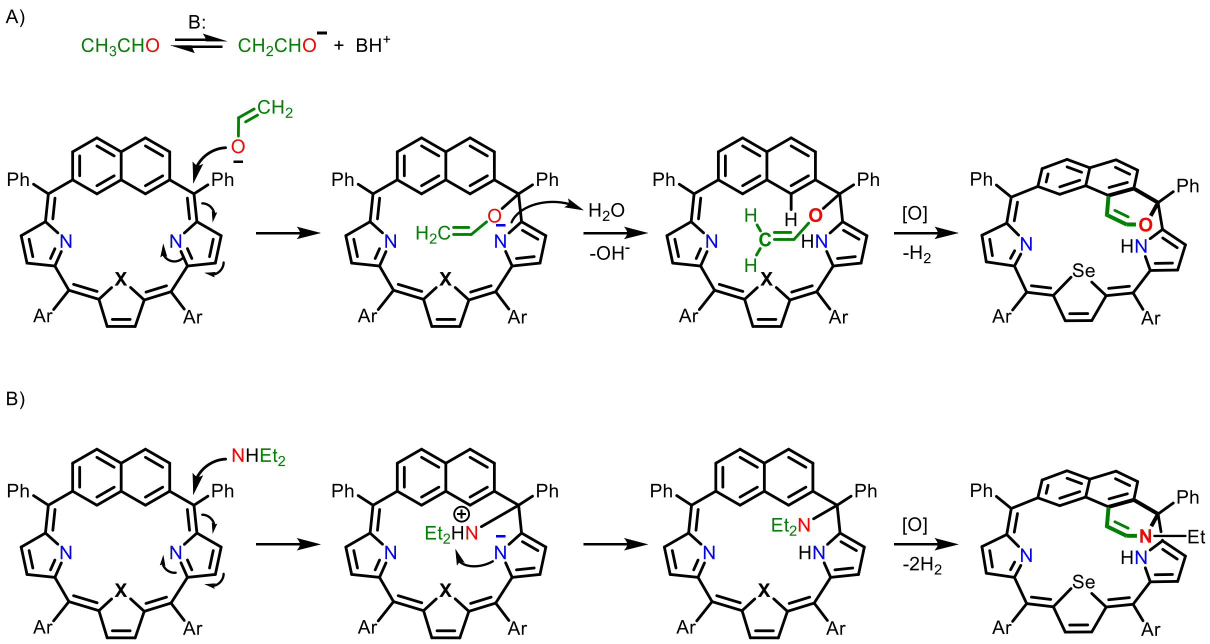

Scheme S1. A plausible mechanism of A) **2-Se** and B) **3-Se** formation.

References

(1) CrysAlis PRO. CrysAlisPro: Rigaku Oxford Diffraction 1.171.41.80a.
(2) Sheldrick, G. M. A Short History of *SHELX*. *Acta Crystallogr. A* **2008**, *64* (1), 112–122.
(3) Sheldrick, G. M. Crystal Structure Refinement with *SHELXL*. *Acta Crystallogr. Sect. C Struct. Chem.* **2015**, *71* (1), 3–8.
(4) Armarego, W. L. F.; Chai, C. L. L. Purification of Laboratory Chemicals, 6th ed.; Elsevier/BH: Oxford, 2009.
(5) Swift, E. The Densities of Some Aliphatic Amines. *J. Am. Chem. Soc.* **1942**, *64* (1), 115–116.
(6) Inman, M.; Carbone, A.; Moody, C. J. Two-Step Route to Indoles and Analogues from Haloarenes: A Variation on the Fischer Indole Synthesis. *J. Org. Chem.* **2012**, *77* (3), 1217–1232.
(7) You, Y.; Gibson, S. L.; Hilf, R.; Ohulchanskyy, T. Y.; Detty, M. R. Core-Modified Porphyrins. Part 4: Steric Effects on Photophysical and Biological Properties in Vitro. *Bioorg. Med. Chem.* **2005**, *13* (6), 2235–2251.
(8) Církva, V.; Jakubík, P.; Strašák, T.; Hrbáč, J.; Sýkora, J.; Císařová, I.; Vacek, J.; Žádný, J.; Storch, J. Preparation and Physicochemical Properties of [6]Helicenes Fluorinated at Terminal Rings. *J. Org. Chem.* **2019**, *84* (4), 1980–1993.
(9) Szyszko, B.; Pacholska-Dudziak, E.; Latos-Grażyński, L. Incorporation of the 1,5-Naphthalene Subunit into Heteroporphyrin Structure: Toward Helical Aceneporphyrinoids. *J. Org. Chem.* **2013**, *78* (10), 5090–5095.
(10) Szyszko, B.; Matviyishyn, M.; Hirka, S.; Pacholska-Dudziak, E.; Białońska, A.; Latos-Grazyński, L. 28-Hetero-2,7-Naphthiporphyrins: Horizontal Expansion of the m-Benziporphyrin Macrocycle. *Org. Lett.* **2019**, *21* (17), 7009–7014.
